# Rapid Outgassing of Hydrophilic TiO_2_ Electrodes Achieves Long-Term Stability of Anion Exchange Membrane Water Electrolyzers

**DOI:** 10.1007/s40820-025-01696-2

**Published:** 2025-03-13

**Authors:** Shajahan Shaik, Jeonghyeon Kim, Mrinal Kanti Kabiraz, Faraz Aziz, Joon Yong Park, Bhargavi Rani Anne, Mengfan Li, Hongwen Huang, Ki Min Nam, Daeseong Jo, Sang-Il Choi

**Affiliations:** 1 Department of Chemistry and Green-Nano Materials Research Center, Kyungpook National University, Daegu, 41566 South Korea; 2 Department of Mechanical Engineering, Kyungpook National University, Daegu, 41566 South Korea; 3 Department of Chemistry and Chemistry Institute for Functional Materials, Pusan National University, Busan, 46241 South Korea; 4 Department of Metallurgical and Materials Engineering, National Institute of Technology, Raipur, 492010 India; 5 College of Materials Science and Engineering, Hunan University, Changsha, 410082 Hunan People’s Republic of China

**Keywords:** TiO_2_ nanotubes, NiFe, Super-hydrophilic electrode, Oxygen evolution reaction, Anion-exchange membrane water electrolyzer

## Abstract

The state-of-the-art anion-exchange membrane water electrolyzers (AEMWEs) require highly stable electrodes for prolonged operation. The stability of the electrode is closely linked to the effective evacuation of H_2_ or O_2_ gas generated from electrode surface during the electrolysis. In this study, we prepared a super-hydrophilic electrode by depositing porous nickel–iron nanoparticles on annealed TiO_2_ nanotubes (NiFe/ATNT) for rapid outgassing of such nonpolar gases. The super-hydrophilic NiFe/ATNT electrode exhibited an overpotential of 235 mV at 10 mA cm^−2^ for oxygen evolution reaction in 1.0 M KOH solution, and was utilized as the anode in the AEMWE to achieve a current density of 1.67 A cm^−2^ at 1.80 V. In addition, the AEMWE with NiFe/ATNT electrode, which enables effective outgassing, showed record stability for 1500 h at 0.50 A cm^−2^ under harsh temperature conditions of 80 ± 3 °C.

**Supplementary Information:**

The online version contains supplementary material available at 10.1007/s40820-025-01696-2.

## Highlights

- A super-hydrophilic electrode was successfully developed by depositing porous NiFe nanoparticles onto annealed TiO_2_ nanotubes (NiFe/ATNT), facilitating rapid outgassing of nonpolar gases.
- The NiFe/ATNT electrode demonstrated an overpotential of 235 mV at 10 mA cm^−2^ for the oxygen evolution reaction in 1.0 M KOH and served as the anode in the anion exchange membrane water electrolyzer (AEMWE), achieving a current density of 1.67 A cm^−2^ at 1.80 V.
- The AEMWE utilizing the NiFe/ATNT electrode exhibited remarkable stability, maintaining operation for 1500 h at 0.50 A cm^−2^ under challenging thermal conditions of 80 ± 3 °C.

**Supplementary Information:**

The online version contains supplementary material available at 10.1007/s40820-025-01696-2.

## Graphical abstract

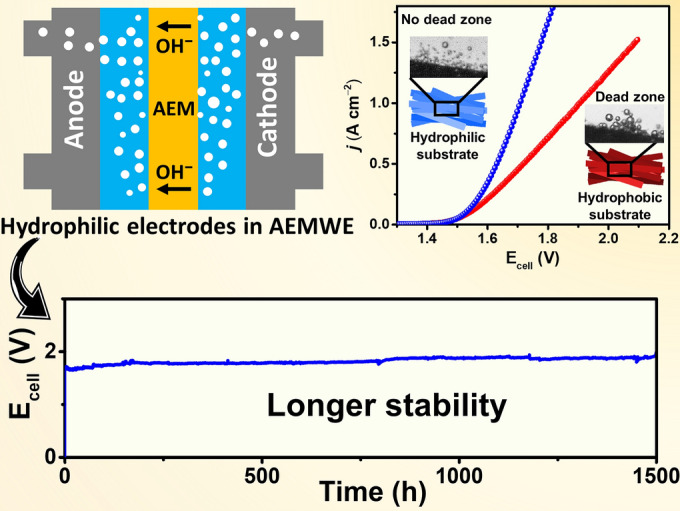

## Introduction

Water electrolyzer is a well-established device that utilizes renewable energy sources to produce green H_2_ [1–7]. Conventional devices include alkaline water electrolyzer (AWE) and proton-exchange membrane water electrolyzer (PEMWE), but AWE suffers from low current density and use of about 40% concentrated KOH solutions, while PEMWE has barriers to megawatt-scale production of H_2_ due to the high cost of electrocatalysts and anti-corrosive components [8, 9]. In recent years, researchers have developed an anion-exchange membrane water electrolyzer (AEMWE) to balance performance and cost [1, 3, 10, 11]. However, achieving high efficiency of hydrogen evolution reaction at the cathode and oxygen evolution reaction (OER) at the anode remain challenges, thus the development of cost-saving electrocatalysts has been extensively investigated [3, 12–20].

Meanwhile, electrocatalyst development alone is limited to improving the performance of electrodes in water electrolyzers [21]. One of the major obstacles in electrode technology that reduces the electrolyzer performance is the accumulation of nonpolar gas bubbles, such as H_2_ and O_2_, on the electrode surface, which blocks the active sites of the catalysts [22–25]. To reduce the bubble adhesion to the electrode surface, electrocatalysts with super-hydrophilic or super-aerophobic surface have been utilized [3, 24–26]. For instance, Wang and coworkers utilized a super-hydrophilic Fe–Ni–P–S catalyst loaded on nickel foam (NF) support as the OER electrode in AEMWE to accelerate the release of O_2_ bubbles from the electrode surface [24]. However, since the generally utilized electrode materials, such as NF, titanium felt (TF), and carbon paper, were hydrophobic, gas bubbles accumulated on the electrode, resulting in particle detachment/agglomeration during the reaction [1]. Therefore, electrode development strategies that combine the advantages of hydrophilicity and effective gas evacuation are essential to advance the long-term operation of electrolyzers. To achieve this, recent researchers have investigated on hydrophilic electrodes with distinct structures, like nanotubes, rather than flat surfaces [27, 28]. The curvature and vertical orientation of nanotubes can reduce the bubble contact area with the electrode, promoting smaller bubble formation and quicker detachment.

This work aims to present super-hydrophilic electrode surface structures that allow O_2_ bubbles to escape efficiently, and to improve OER performances by including electrode materials with high surface area, and enhanced stability. To offer all these advantages, we prepared an annealed TiO_2_ nanotubes (ATNT) with a super-hydrophilic and rough surface structure as a catalyst support by anodizing and annealing of TF [27, 28]. Then, NiFe nanoparticles (NPs), a well-known OER catalyst material, were electrodeposited onto the ATNT (NiFe/ATNT) to elucidate the fascinating properties of super-hydrophilic support. In the NiFe/ATNT ǁ Pt/C/ATNT configuration of the AEMWE cell, as-prepared electrode outperformed IrO_2_ and NiFe catalysts on hydrophobic TF substrates. In addition, the NiFe/ATNT ǁ Pt/C/ATNT demonstrated record stability for 1500 h at 0.50 A cm^−2^ under high temperatures of 80 ± 3 °C, highlighting the potential for commercialization of AEMWE by applying enhanced hydrophilic electrodes.

## Experimental Section

### Preparation of ATNTs

As-purchased TF was cleaned with acetone under ultrasonic treatment for 15 min. Then, rinsed the TF several times with deionized (DI) water and dried in the oven at 60 °C for 20 min. Subsequently, the anodizing process was carried out in a solution containing 95 vol% ethylene glycol, 5 vol% DI water, and 0.75 wt% of NH_4_F using a direct current power source under a constant voltage of 30 V between the anode and the cathode for 6 h. TF was used as the anode, while the aluminum foil was used as the cathode. The distance between the two electrode was ~ 3 cm. After the anodization process, resultant amorphous TiO_2_ nanotube (TNT) electrodes were cleaned with DI water and air-dried. The synthesized TNT was annealed at 500 °C for 5 h in air to transform the amorphous structure to anatase phase.

### Deposition of NiFe-Catalyst on Different Supporting Electrodes

The NiFe/ATNT electrode was prepared using the electrodeposition synthesis process in electrolyte solution (100 mL of DI water) containing NiSO_4_·6H_2_O (2.64 g), FeSO_4_·7H_2_O (0.65 g), Na_3_C_6_H_5_O_7_·2H_2_O (14.70 g), NH_4_Cl (2.67 g), and NaBr (2.04 g). ATNT substrate and pure Ni plate (99%) were used as cathode and anode, respectively. Both electrodes are immersed vertically in the electrolyte solution. The continuous magnetic stirring with 300 rpm was applied to the solution throughout the deposition process for homogeneous deposition. The deposition was performed at a constant current density of 100 mA cm^−2^ and a constant bath temperature of 40 °C. The pH value of the solution was maintained to be ~ 7.0. During the synthesis process, the bath composition and deposition time were varied to control the Ni-to-Fe molar ratios and catalyst loading on ATNT, respectively (Tables S1 and S2). After the electrodeposition process, catalyst-deposited electrodes were cleaned with ethanol and DI water several times to remove the remaining electrolyte solution and dried in an oven at 60 °C for 30 min. As per inductively coupled plasma-atomic emission spectroscopy (ICP-AES) results, the molar ratio of Ni:Fe was determined to be 4:1. To compare the electrocatalytic performance of the NiFe/ATNT catalyst electrode with other supporting electrodes, NiFe/TNT and NiFe/TF catalyst electrodes were also prepared using the same molar ratio (4:1) (Table S3) and conditions.

### Electrochemical Measurements

All the electrochemical measurements were performed at room temperature using a standard three-electrode cell connected to a BioLogic VSP potentiostat with 10 A booster. For improved accuracy in electrochemical measurements, each electrode was measured three times under the same operation conditions. Pt mesh (1 cm^2^) and Hg/HgO were used as counter and reference electrodes for all the electrochemical measurements. On the other hand, the working electrode varied for each case: TF, TNT, ATNT, Ni/ATNT, NiFe/TF, NiFe/TNT, and NiFe/ATNT. Commercial IrO_2_ and Ni powder have been used as OER benchmark catalysts for the comparison. To prepare the commercial IrO_2_ on glassy carbon (GC) electrode (IrO_2_/GC), catalyst ink was prepared by mixing 2.5 mg of catalyst powder, 10 µL of 5 wt% Nafion, 1.0 mL of DI water, and 0.25 mL of isopropyl alcohol, and then the corresponding mixture was homogeneously mixed by ultra-sonication for 30 min. Before the catalyst ink loading, the L-shaped GC electrode was mirror-polished with 0.05 μm alumina powder and washed appropriately with DI water. Then, the prepared catalyst ink was loaded on GC (0.196 cm^2^) with a catalyst loading of 20.4 µg cm^−2^. A similar catalyst ink preparation method was followed to prepare the commercial Ni powder on the NF electrode using 5 mg of Ni catalyst powder. Before loading the catalyst ink, the NF was cleaned with acetone under ultrasonic treatment for 15 min. It was then immersed in a 0.1 mol L⁻^1^ HCl solution at room temperature for 30 min to remove inorganic impurities, followed by rinsing with DI water and drying at 60 °C for 20 min. The prepared catalyst ink was then sprayed onto the NF (1 cm^2^) using a spray gun with a catalyst loading of 5 mg cm^−2^. All applied potential was converted to a reversible hydrogen electrode (*V*_RHE_). At first, the working electrode was activated by cyclic voltammetry (CV) at a scan rate of 100 mV s^−1^ for 20 cycles in between 0.08 to 1.0 *V*_RHE_ in an Ar-saturated 1.0 M KOH solution. The final CV was recorded at a scan rate of 50 mV s^−1^ for 2 cycles in an Ar-saturated 1.0 M KOH solution. Afterward, linear sweep voltammetry (LSV) for OER was performed at a scan rate of 5 mV s^−1^ in the potential range of 1.0 to 1.80 V_RHE_ in an O_2_-saturated 1.0 M KOH solution. Except for CV, all electrochemical investigations were conducted under O_2_-saturated conditions to affirm the O_2_/H_2_O equilibrium at 1.23 V_RHE_. The chronopotentiometric stability test for prepared electrodes was conducted for 100 h at a current density of 10 mA cm^−2^ in an O_2_-saturated 1.0 M KOH solution. See the Supporting Information for more details about electrochemical measurements.

### Preparation of Electrodes for AEMWE

To prepare the electrodes, catalyst-coated substrate (CCS) method was followed. The NiFe on ATNT (3 × 3 cm^2^) was prepared by the electrodeposition process as reported in the experimental section. The Pt/C/ATNT electrode was prepared using a spray coating method. Catalyst ink was prepared by mixing 15.62 mg of commercial Pt/C (20 wt%) catalyst powder, 200 µL of the respective commercially available ionomer for each membrane, 0.2 mL of DI water, and 0.8 mL of isopropyl alcohol. The mixture was then homogeneously sonicated for 60 min. Finally, the prepared catalyst ink was uniformly sprayed onto the ATNT substrate (2.5 × 2.5 cm^2^) using a spray gun. Each electrode was separately hot-pressed at 60 °C and 4 MPa for 10 s to ensure proper catalyst adhesion to the support before being incorporated into the membrane electrode assembly (MEA). In case of the Fumasep membrane, a hot pressing machine was used to press the membrane with electrodes.

### Fabrication of MEA

The MEA was carefully prepared while assembling the electrolyzer. Optimized NiFe/ATNT and commercial catalyst Pt/C/ATNT electrodes were used as the anode and cathode, respectively. A pre-treated membrane (see the Supporting Information) was placed between the cathode and anode like a sandwich. Then, Pt-coated Ti mesh was positioned on the backside of both the anode and cathode to improve the electrical conductivity between the bipolar plates and the electrodes. After assembling all the components, the electrolyzer was manually tightened using a wrench.

### Electrochemical Measurements of Assembled AEMWE

After assembling the electrolyzer, the electrochemical measurements were conducted using 1.0 M KOH solution at different temperatures (25, 40, 50, 60, and 80 ± 3 °C), different electrolyte flow rates on the anode side (1, 2, 3, and 5 mL min^−1^), and with different AEMs. Each experiment was conducted at least three times to ensure data accuracy. The electrochemical impedance spectroscopy (EIS) of the AEMWE cell was measured at 1.40 and 1.60 V. A long-term stability test was performed for 1500 h in an aqueous 1.0 M KOH solution at a current density of 0.50 A cm^−2^ at 80 ± 3 °C. The dynamic operational durability of the AEMWE system was evaluated using square-wave cycling tests under varying voltage conditions. The system was subjected to 100 cycles between 0.10 to 1.60 V, and 50 cycles between 0.10 and 1.80 V in 1.0 M KOH, low-alkaline (0.1 M KOH), and DI water electrolytes at 80 ± 3 °C. During each cycle, the cell voltage was held at 0.10 and 1.60 V, and subsequently at 0.10 to 1.80 V, for 1 min each. To compare the performance, an AEMWE cell was also prepared using commercial catalysts such as 20 wt% Pt/C/ATNT (loading amount of 2.5 mg cm^−2^) as a cathode and IrO_2_/ATNT (loading amount of 3 mg cm^−2^) as an anode and examined under the same conditions. More details can be found in Supporting Information.

### Photoelectrochemical (PEC) Measurements

The PEC measurements were conducted under simulated solar light irradiation [29, 30]. The working electrode, with an actual geometric area of 1 cm^2^, was exposed to an electrolyte solution. The intensity of the solar simulator (150 W xenon lamp, ABET technologies) was calibrated to AM 1.5G (1 SUN, 100 mW cm^−2^). The anodic photocurrent value and external bias vs. the reference electrode (Hg/HgO) were controlled and monitored using a CHI 630 potentiostat (Austin, TX). The PEC measurements were performed in aqueous solutions of 0.1 M KOH with a scan rate of 20 mV s^−1^.

## Results and Discussion

### Synthesis and Characterization of ATNT-Based Electrodes

**Fig. 1:**
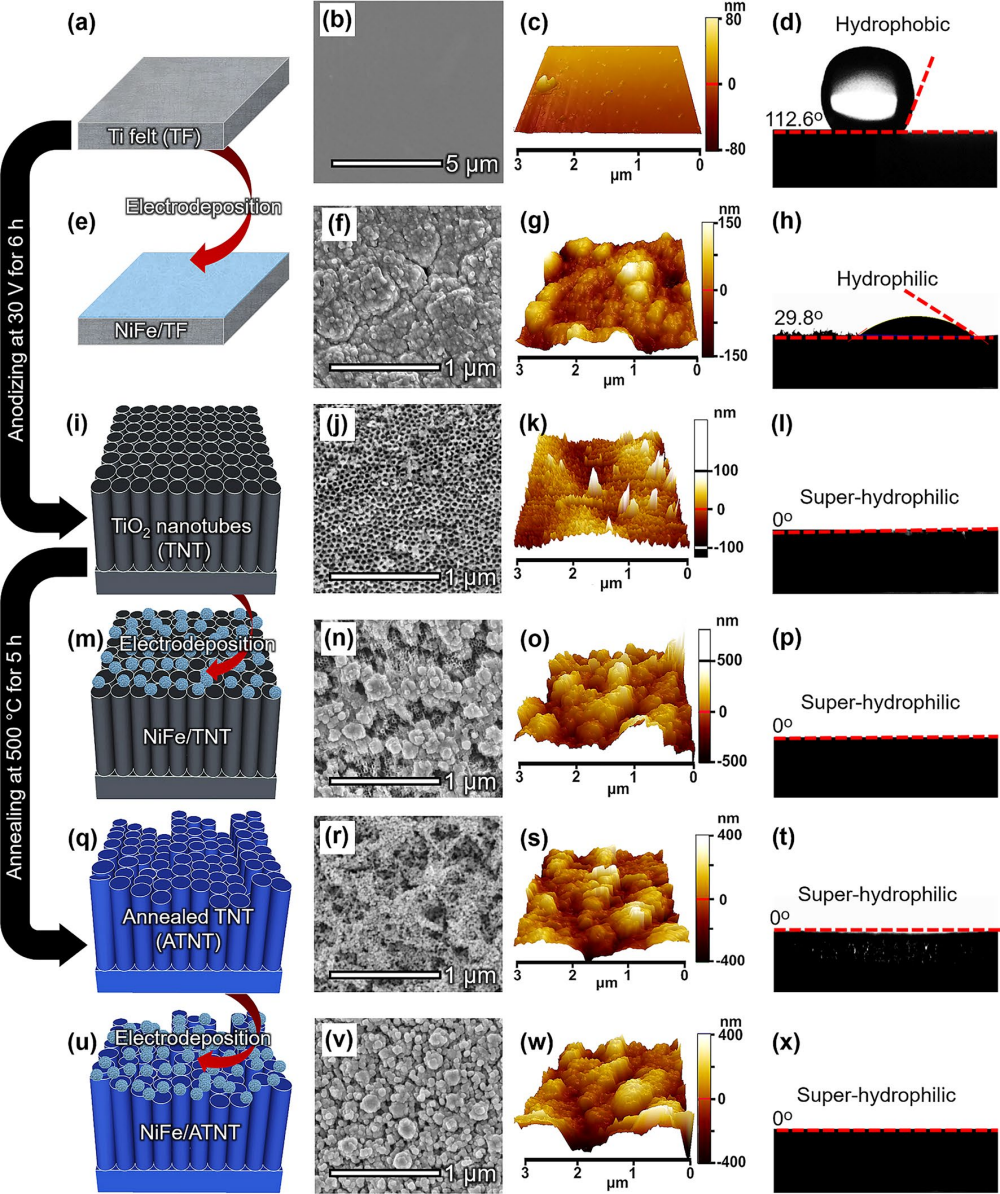
Schematic illustration and structure analysis of NiFe NPs on different Ti-based substrates. The schematic illustrations of the preparation process, corresponding FESEM images, AFM images, and contact angles of a water droplet for various Ti-based substrates: **a–d** TF, **e–h** NiFe/TF, **i–l** TNT, **m–p** NiFe/TNT, **q–t** ATNT, and **u–x** NiFe/ATNT

We prepared various Ti-based substrates for the OER electrode by anodizing and annealing treatments on TF (Fig. 1). Pristine TF with a smooth surface was shown in field emission scanning electron microscopy (FESEM) (Fig. 1b) and atomic force microscopy (AFM) images (Fig. 1c) with an average surface roughness (the root-mean-square value of the image pixel height, *R*_q_) of 18 nm over the surface area of 3 μm^2^. Synchrotron X-ray diffraction (SXRD) demonstrated the hexagonal-close-packed Ti (Ti_hcp_) metal crystal structure (Fig. S1a). After anodization of TF (see Experimental Section) [27, 28], TiO_2_ nanotubes were formed on the TF surface (TNT) (Fig. 1j), increasing the *R*_q_ value of 22 nm (Fig. 1k). Since the TNT was amorphous, it showed similar SXRD patterns to the pristine TF (Fig. S1b) [27]. In addition, the TNT exhibited low conductivity, so an annealing treatment was applied to convert the amorphous phase into a crystalline anatase phase of TNT (ATNT) [28]. The phase transformation of TNT to ATNT also led to three-dimensional hierarchical porous structure formation, combining both small and large pores, with a highly increased *R*_q_ value of 117 nm (Fig. 1r, s). The SXRD analysis of the ATNT showed distinct anatase-TiO_2_ (101) and (200) peaks at 25.3° and 48.1°, respectively (Fig. S1c). X-ray adsorption fine structure (XAFS) analysis was conducted to confirm the surface structural transformation of TF during the treatments. The X-ray absorption near edge structure (XANES) showed clear pre-edge attributed to a dipole 1*s* to 3*d* transition of metallic Ti for TF, TNT, and ATNT similar to Ti foil (Fig. S2a). However, TNT and ATNT also showed quadrupole pre-edges owing to the formation of TiO_2_ [31]. Fourier transform-extended X-ray adsorption fine structure (FT-EXAFS) spectra of TNT and ATNT presented two distinct peaks of Ti–O (1.5 Å) and extended Ti–Ti (2.6 Å) bonds indicating TiO_2_ formation while the TF presented a single Ti–Ti (2.5 Å) peak (Fig. S2b). The extended Ti–Ti bond of TNT was slightly shorter than that of ATNT, indicating the amorphous structure of TNT [32].

**Fig. 2:**
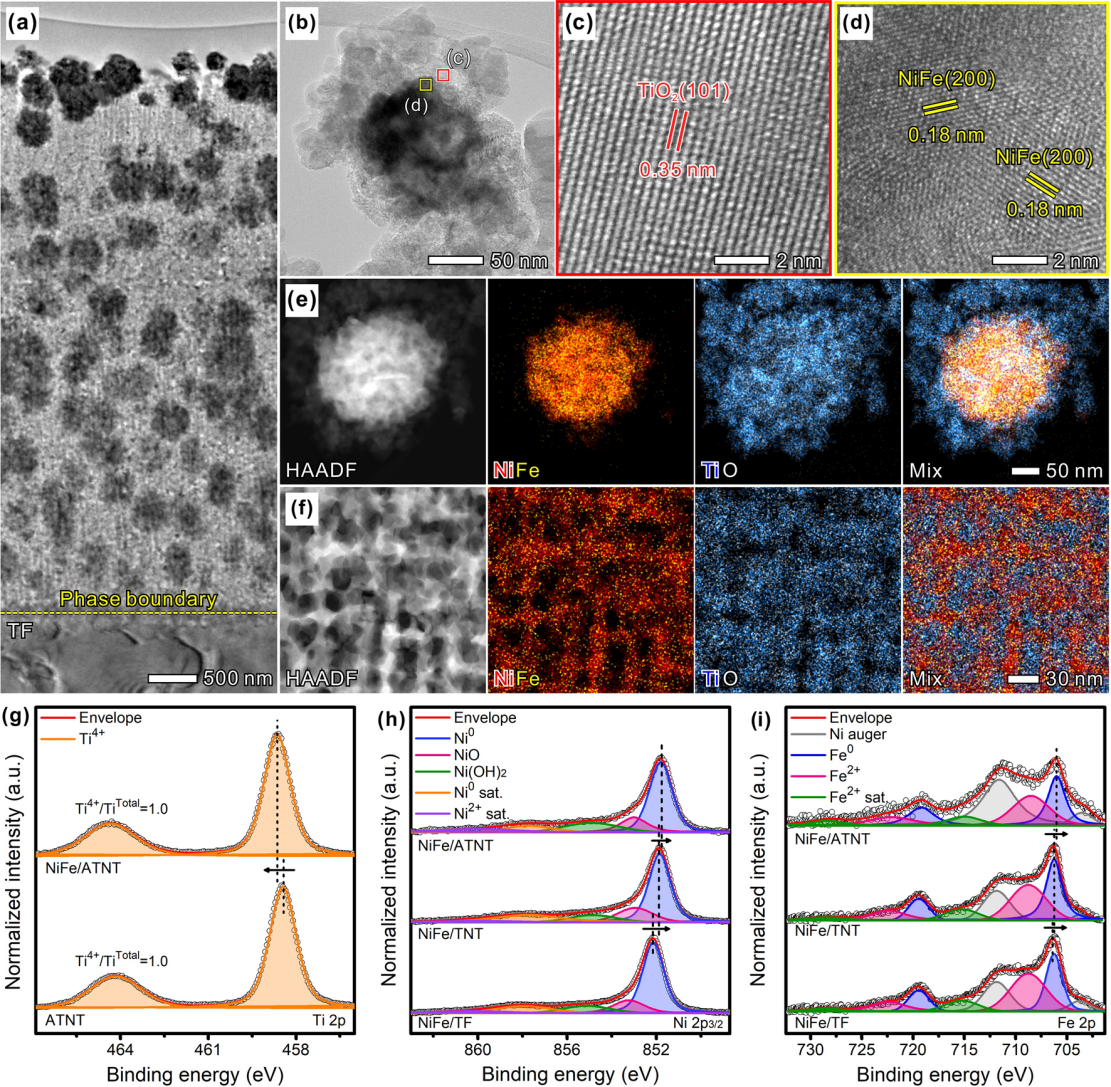
Material characterization of the prepared electrodes. **a** Cross-sectional HRTEM image of FIB cut NiFe/ATNT. **b** HRTEM images of NiFe NPs on ATNT. **c, d** Magnified viewing taken from the red and yellow boxes in **b**. HAADF-STEM image and EDS elemental mapping of a single NiFe nanoparticle of **e** NiFe/ATNT and **f** FIB cut NiFe/ATNT. **g** XPS spectra on Ti 2*p* region for ATNT and NiFe/ATNT. XPS spectra for NiFe/ATNT, NiFe/TNT, and NiFe/TF on **h** Ni 2*p*_3/2_ and **i** Fe 2*p* regions

To demonstrate the different surface types of Ti-based substrates, NiFe alloy, which was one of the promising catalytic materials for OER, was electrochemically deposited on them to obtain NiFe/TF, NiFe/TNT, and NiFe/ATNT. The NiFe/TF electrode showed a thick surface coating with agglomerated NiFe NPs (Fig. 1f), while both the NiFe/TNT (Fig. 1n) and NiFe/ATNT (Fig. 1v) displayed less agglomeration of NiFe NPs. The *R*_q_ values were measured to be 43, 111, and 120 nm for NiFe/TF (Fig. 1g), NiFe/TNT (Fig. 1o), and NiFe/ATNT (Fig. 1w), respectively, indicating that the higher *R*_q_ value of the ATNT provides more nucleation sites for the formation of smaller sized and higher dispersed NiFe NPs than the other electrodes. The presence of the NiFe peaks at 44.0° and 51.5° in SXRD confirmed the formation of NiFe NPs on these electrodes (Fig. S1). The distribution of NiFe NPs within TF, TNT, and ATNT was also confirmed by cross-sectional FESEM images before and after NiFe deposition (Fig. S3). The FESEM images of NiFe/TF and NiFe/TNT showed that NiFe NPs attached on the surface of TF and TNT. However, NiFe NPs were formed not only on the surface but also on the inside of ATNT due to the high roughness and porous structure of ATNT. The cross-sectional transmission electron microscopy image of NiFe/ATNT was further examined to show the well-dispersed NiFe NPs within the ATNT (Fig. 2a). The high-resolution transmission electron microscopy (HRTEM) images of NiFe/ATNT revealed the lattice distances of 0.35 and 0.18 nm corresponding to the anatase TiO_2_ (101) and NiFe (200), respectively (Fig. 2b–d) [33–35]. The high-angle annular dark field scanning TEM (HAADF-STEM) image and energy-dispersive X-ray spectroscopy (EDS) elemental mapping of NiFe/ATNT revealed the formation of NiFe alloy on TiO_2_ substrate without any doping of Ni or Fe atoms in TiO_2_ (Fig. 2e). Furthermore, HAADF-STEM and EDS mapping images prepared by focused ion beam (FIB) cutting showed internal nanoporous structure of NiFe NP (Fig. 2f). Consequently, well-dispersed NiFe NPs were successfully synthesized from surface to inner side of ATNT substrate, due to the porous and highly rough structure of ATNT which may offer improved OER performance by efficient mass transfer of electrolyte/gas [24, 36–38].

X-ray photoelectron spectroscopy (XPS) was used to examine the chemical structure of the prepared electrodes. The Ti 2*p* XPS spectrum of pristine TF showed Ti^0^ peaks and minor Ti^x+^ peaks, which are from the naturally oxidized Ti on the surface, while the TNT and ATNT exposed dominant Ti^4+^ and minor Ti^2+^ peaks (Figs. 2g and S4). After NiFe deposition, no Ti 2*p* peaks were observed due to the existence of thick NiFe layer on the flat TF surface (Fig. S4a). In the cases of TNT and ATNT, the oxidation state of Ti increased after NiFe deposition. Specifically, the Ti^4+^/Ti^Total^ ratio increased from 0.91 of TNT to 0.98 of NiFe/TNT (Fig. S4b). In addition, the peak position of Ti^4+^ for NiFe/ATNT was blue-shifted to that for ATNT. These are the results of electron transfer from TiO_2_ to Ni/Fe due to the lower work function value of TiO_2_ (4.12 eV) than Ni (5.15 eV) and Fe (4.33 eV) [39, 40]. In this regard, the Ni 2*p*_3/2_ and Fe 2*p* XPS spectra were red-shifted for NiFe/TNT and NiFe/ATNT compared with NiFe/TF, with NiFe/ATNT showing more shift than NiFe/TNT (Fig. 2h, i). This larger peak shift could be attributed to the higher *R*_q_ value of ATNT than TF and TNT, which helped to increase interaction sites between the NiFe NPs and the ATNT during electrodeposition. The HRTEM image at the interfacial region of NiFe and TiO_2_ showed atomic defects, supporting the presence of chemical interaction between NiFe and TiO_2_, which could prevent catalyst degradation (e.g., particle aggregation and detachment) and enhance electrical resistance (Fig. S5). The XAFS analysis also exhibited consistent phenomena well matched with XPS results. The XANES spectra of the Ni K-edge showed that the white line location of the electrodes was red-shifted in the order of NiFe/ATNT < NiFe/TNT < NiFe/TF < Ni foil, indicating more reduced states of Ni in NiFe/ATNT (Fig. S6a). Because of the strong interaction between TiO_2_ and NiFe nanocrystals, electron transfer from TiO_2_ to Ni/Fe creates an excess electron state on the metal surface. The excessive electron state will be rearranged and accumulated near the surface of metal atoms, resulting in the negatively charged state of Ni/Fe [41]. The Fe K-edge XANES spectra demonstrated a similar trend to the Ni K-edge (Fig. S6c). The FT-EXAFS profiles of Ni K-edge and Fe K-edge for the NiFe/TF, NiFe/TNT, and NiFe/ATNT exhibited metallic structures (Fig. S6b, d). The difference between the Fe K-edge peak patterns of NiFe crystal and Fe foil was attributed to the differences in crystal structure between them [42].

The hydrophilicity of the prepared electrodes was analyzed by the wettability test. The pristine TF is hydrophobic, as indicated by the contact angle of 112.6° to the water droplet (Fig. 1d). However, due to the hydrophilic NiFe deposition, the NiFe/TF decreased the contact angle to 29.8° (Fig. 1h) [24, 25]. On the other hand, the TNT and ATNT exhibited hydrophilic properties without forming a measurable contact angle due to the formation of polar TiO_2_ on the surfaces (Fig. 1l, t). In addition, the hydrophilic electrodes may enhance the capillary effect, allowing water to be rapidly attracted and spread across the surface. These hydrophilic behaviors were maintained after the deposition of the NiFe onto the TNT and ATNT (Fig. 1p, x). A similar trend was also observed with commercial powder catalysts, where IrO_2_, Pt/C, and Ni powder on ATNT and TNT supports exhibited super-hydrophilic properties compared to those on TF substrates (Fig. S7). The hydrophilic nature of these surfaces allows unimpeded access of the electrolyte to the catalyst during electrochemical reactions, promoting the reaction kinetics and thus enhancing electrocatalytic performances [24, 25, 38].

The super-aerophobic properties of the electrodes were further evaluated using underwater contact angle measurements. The pristine TF was aerophobic with a contact angle of 92.2° (Fig. S8a). However, due to the super-aerophobic NiFe coating, the contact angle of NiFe/TF increased to 133.1° (Fig. S8d). TNT and ATNT displayed even greater super-aerophobic properties (Fig. S8b, c), with contact angles of 145.4° and 155.2°, respectively, which were further enhanced after NiFe deposition (Fig. S8e, f). NiFe/ATNT showed a higher contact angle (157.3°) compared to NiFe/TNT (151.5°), likely due to higher surface roughness. A similar pattern was observed with commercial powder catalysts, where electrodes like IrO_2_/ATNT, Pt/C/ATNT, and Ni powder/ATNT exhibited superior super-aerophobic properties compared to their counterparts on TNT and TF substrates (Fig. S9). These findings suggest that ATNT support provides high surface super-aerophobicity and optimal wetting, enabling efficient bubble release and exposure of catalytic sites, which enhances electrocatalytic performance.

### Electrochemical Measurements

**Fig. 3:**
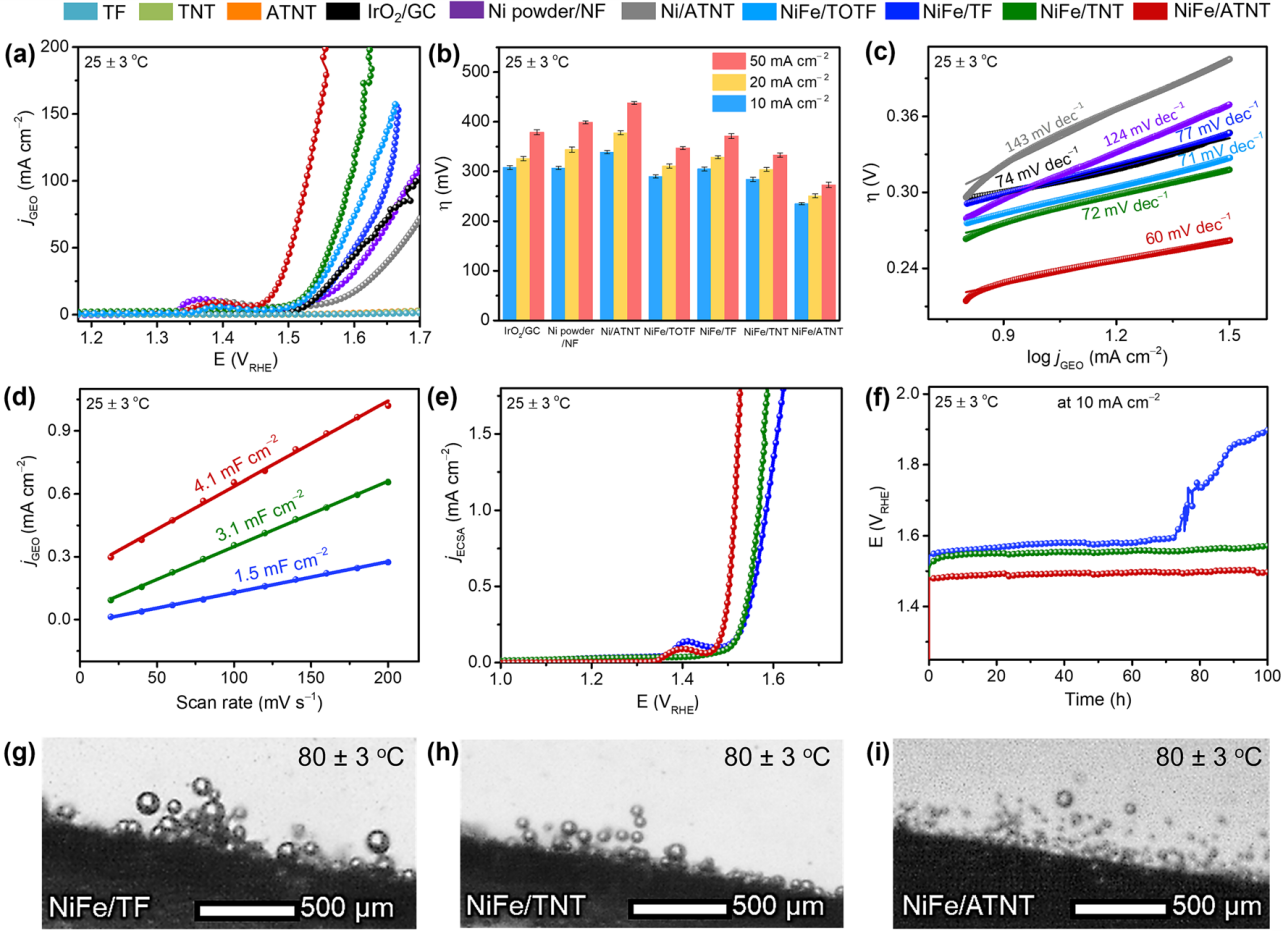
OER catalytic performances of as-prepared electrodes: **a** 100% *iR*-corrected OER polarization curves, **b** overpotential values, **c** OER Tafel plots, **d** Plots of double layer charge current vs. scan rate, **e** ECSA-normalized OER polarization curves, and **f** OER stability test. Images capturing gas bubbles releasing from **g** NiFe/TF, **h** NiFe/TNT, and **i** NiFe/ATNT electrodes during half-cell test at 80 ± 3 °C with a high-definition camera

Electrochemical measurements of NiFe NPs formed on different Ti-based substrates were conducted in 1.0 M KOH solution using a three-electrode system. The OER polarization curves were obtained via LSV at a scan rate of 5 mV s^−1^ with 100% *iR-*correction (Fig. 3a). The evaluation of OER performance for NiFe/ATNT catalysts with different Ni-to-Fe molar ratios and catalyst loadings revealed that a ratio of 4:1 and a loading of 5 mg cm⁻^2^ were found to be optimal conditions (Figs. S10-S12, Tables S1, and S2). To provide a basis for comparison, the OER performances of bare TF, TNT, ATNT, Ni/ATNT, commercial Ni powder/NF, and commercial IrO_2_/GC, were investigated under the same conditions. Furthermore, we prepared NiFe deposited on a calcined TF electrode forming TiO_2_ layer on the surface (NiFe/TOTF) (see Supporting Information for details, Fig. S13) to emphasize the necessity of the ATNT preparation. To achieve a current density of 10 mA cm^−2^, the NiFe/ATNT demonstrated the overpotential of 235 mV, which was 73, 72, 70, 49, 104, and 55 mV lower than the IrO_2_/GC, Ni powder/NF, NiFe/TF, NiFe/TNT, Ni/ATNT, and NiFe/TOTF, respectively. In addition, Fig. 3b showed the comparison of overpotential values at current densities of 10, 20, and 50 mA cm^−2^, indicating that the NiFe/ATNT showed the best OER activity among all the electrodes. The reaction kinetics of the prepared electrodes were determined by the Tafel slope derived from the steady-state polarization curve, as shown in Fig. 3c. The Tafel slope values of IrO_2_/GC, Ni powder/NF, Ni/ATNT, NiFe/TF, NiFe/TNT, NiFe/ATNT, and NiFe/TOTF were measured to be 74, 124, 143, 77, 72, 60, and 71 mV dec^−1^, respectively, suggesting the fastest charge-transfer kinetics of NiFe/ATNT [12].

The Nyquist plots of prepared electrodes showed that NiFe/ATNT exhibited lower charge transfer resistance (smaller arc diameter) than NiFe catalysts on TF and TNT, indicating performance improvement due to relatively faster electron transfer (Fig. S14) [43, 44]. In addition, PEC characterization was conducted under solar irradiation in 0.1 M KOH for the prepared electrodes before and after NiFe deposition, indicating the negligible semiconducting properties of the TiO_2_ (Fig. S15) [45].

The electrochemical active surface area (ECSA) of the electrocatalysts was estimated by measuring double layer capacitance (*C*_dl_) from CV curves recorded at different scan rates in the non-faradic region with a potential range of 0.52–0.62 *V*_RHE_ (Fig. S16). Since the *C*_dl_ is proportional to the ECSA of the electrode [46], the higher the ECSA, the more active sites on the electrode surface, leading to a faster charge-transfer rate and stronger interaction between the electrolyte and electrode during the OER [12, 46–51]. As shown in Fig. 3d, *C*_dl_ was measured to be 4.1 mF cm^−2^ for the NiFe/ATNT, which is higher than those of NiFe/TF (1.5 mF cm^−2^), and NiFe/TNT (3.1 mF cm^−2^). Therefore, the increased ECSA of the NiFe/ATNT can be attributed to the utilization of porous NiFe NPs [36], and enhanced surface roughness [48]. In addition, the normalized LSV plot by the ECSA (Fig. 3e) showed that NiFe/ATNT exhibits superior intrinsic activity compared to NiFe/TF and NiFe/TNT, indicating that the higher activity can be attributed to the hydrophilic properties of ATNT [52].

To evaluate the long-term stability of the prepared catalysts, a chronopotentiometric test at a constant current density of 10 mA cm^−2^ in 1.0 M KOH solution was conducted for 100 h (Fig. 3f). The results revealed that the NiFe/TF maintained its performance for up to 72 h. On the other hand, the NiFe/TNT and NiFe/ATNT performed for 100 h without a significant increase in overpotentials. The stable OER performances of NiFe/TNT and NiFe/ATNT can be attributed to the hydrophilicity effect of the electrodes [53, 54]. To evaluate the impact of hydrophilic electrodes on catalyst stability, we performed TEM, XRD, XPS and FESEM analyses on the electrodes after 100 h of OER testing. The TEM and XRD results of NiFe/ATNT exhibited very similar physical properties before and after 100 h of OER test (Fig. S17). By the way, the XPS results for the Ni 2*p*_3/2_ and Fe 2*p* regions predominantly showed the + 3 oxidation states, indicating the formation of the OER-active NiFeOOH phase on the surface during the stability test (Fig. S18a, b). In addition, as shown in Fig. S18c, quantitative XPS analysis showed that NiFe/ATNT and NiFe/TNT had minimal chemical degradation with 3.0% and 3.4% Fe/Ni ratio decreases, respectively, compared to their fresh conditions. In contrast, NiFe/TF exhibited a notable 5.4% lower Fe/Ni ratio than its pristine one. This Fe dissolution might be attributed to the dead zone formation on the hydrophobic TF electrode, which surfaces intensifies local overpotential, accelerate Fe dissolution, and shorten the catalyst lifespan [22, 55]. Post-FESEM analysis revealed that NiFe/TF demonstrated considerable structural damage (Fig. S19a, d), NiFe/TNT showed moderate changes (Fig. S19b, e), whereas NiFe/ATNT exhibited almost no morphological changes (Fig. S19c, f). These observations suggest that the super-hydrophilic ATNT substrate, with its rough, three-dimensional hierarchical porous structure, is more effective in reducing bubble accumulation, enhancing gas evacuation, and minimizing structural and chemical damages, thereby improving long-term stability. In contrast, hydrophobic TF surfaces cause bubble accumulation, which promotes Fe dissolution under OER conditions, leading to increased structural and chemical damages and ultimately reducing the lifespan of the catalyst.

The influence of the hydrophilic behavior on the gas (O_2_) bubble release of the electrodes during electrochemical measurements was investigated by utilizing a high-definition camera (Fig. S20) to capture and analyze the bubble release patterns on the prepared electrodes during the OER. We conducted this experiment in a half-cell configuration at 80 ± 3 °C. When examining NiFe/TF electrodes, the O_2_ bubbles formed on the electrode surface adhered strongly and connected to adjacent bubbles to form a network of bubbles (Fig. 3g). Consequently, these bubbles grew in size and formed poor mass transfer zones, often known as “dead zones,” on the electrode surface [53]. As illustrated in earlier studies, dead zones restrict the influx of reactants into the electrode, causing an inadequate supply of reactants in these regions and subsequently resulting in a diminished reaction, as observed in this case with the OER. The sizes of released bubbles were noticeably reduced on the surface of the NiFe/TNT compared to those observed on the NiFe/TF (Fig. 3h). Although the NiFe/TNT was hydrophilic, its flat surface prevented the bubbles from detaching quickly, forming dead zones. For the NiFe/ATNT, we observed that the generated O_2_ bubbles were much smaller than those of the other two electrodes and quickly escaped from the electrode surface, limiting the creation of dead zones on the electrode surface (Fig. 3i). Additionally, live observations of gas bubbles release pattern from the prepared electrodes during the half-cell test further confirmed these results, as detailed in Video S1. These results showed that the ATNT substrate with its high surface roughness, hydrophilicity, and hierarchical porous structure enhances the surface area-to-volume ratio, providing numerous bubble nucleation sites and reducing bubble contact with the electrode. Additionally, the smaller NiFe NPs on the ATNT substrate further increased nucleation sites due to the high surface area of the electrode. The synergistic effects promoted the rapid formation and detachment of smaller bubbles, reduction of dead zones, exposure of more active sites, and enhancement of electrochemical performance at high current densities [24, 38, 48, 54]. The average diameters of the O_2_ bubbles released from the electrode surfaces were measured to be 124 ± 13, 75 ± 6, and 23 ± 8 µm for NiFe/TF, NiFe/TNT, and NiFe/ATNT, respectively (Fig. S21).

### AEMWE Performance

**Fig. 4:**
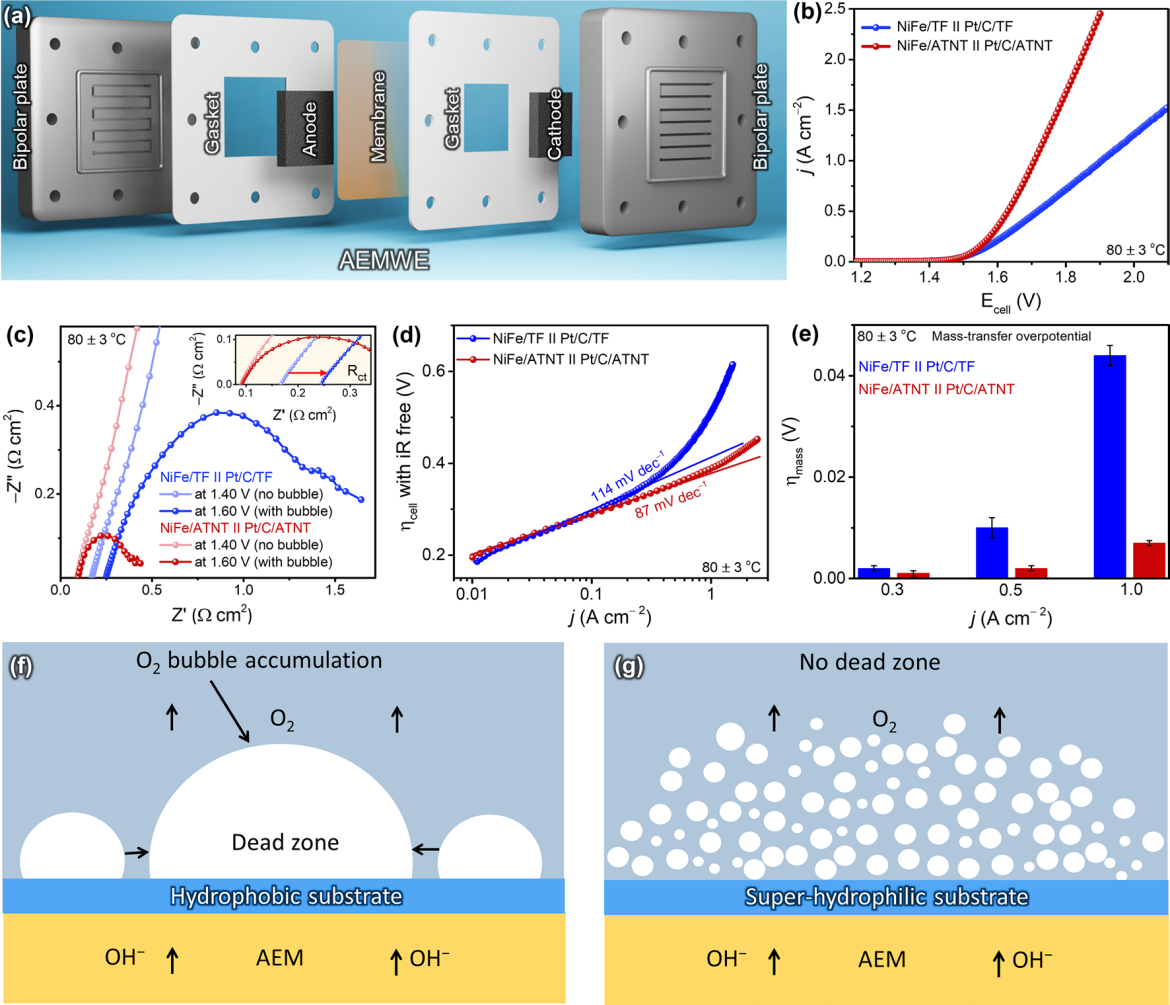
The AEMWE performance of as-prepared NiFe/ATNT electrodes. **a** Schematic image of AEMWE configuration. **b** LSV curves for the AEMWE with different setups: NiFe/TF ‖ Pt/C/TF and NiFe/ATNT ‖ Pt/C/ATNT with a flow rate of 3 mL min^−1^ at a scan rate of 5 mV s^−1^ and at 80 ± 3 °C. **c** EIS analysis of NiFe/TF ‖ Pt/C/TF and NiFe/ATNT ‖ Pt/C/ATNT setups: a comparative study at 1.40 V (no bubble generation) and 1.60 V (with bubble generation) at 80 ± 3 °C. **d** 100% *iR*-corrected Tafel plots derived from LSV curves. **e** Mass-transfer overpotential (η_mass_) of AEMWE with different setups: NiFe/TF ‖ Pt/C/TF and NiFe/ATNT ‖ Pt/C/ATNT at various current densities. Schematics images of **f** O_2_ bubble accumulation on hydrophobic NiFe/TF and **g** O_2_ bubble evacuation on hydrophilic NiFe/ATNT during electrolysis

To evaluate the potential applicability of NiFe/ATNT, water electrolysis experiments were performed using a custom-made single-cell electrolyzer with a surface area of 5 cm^2^ (Figs. 4a and S22a). The electrical resistivity of as-prepared electrodes was priorly tested by the four-point probe resistivity method to convince the AEMWE suitability (Table S4). After anodization, TNT (1.37 mΩ cm) and ATNT (1.35 mΩ cm)-based electrodes exhibited slightly increased electrical resistivity than TF (0.54 mΩ cm), but the values remained still metallic range (10^−8^ to10^−6^ Ω m). After the deposition of the NiFe catalyst onto the respective substrates, the resistivity values were changed to 0.50 mΩ cm for NiFe/TF, 1.18 mΩ cm for NiFe/TNT, and 1.22 mΩ cm for NiFe/ATNT. The slightly lower resistivity of NiFe/TNT than NiFe/ATNT can be attributed to the larger size of NiFe NPs, which enhances electrical conductivity [56]. These results indicate that the thin TiO_2_ layer had a negligible impact on the overall electrical resistance of the electrodes. The NiFe/ATNT was used as the anode and a commercial Pt/C sprayed on the ATNT was used as the cathode. While most reported electrolyzer setups employed a 40 wt% of Pt/C on their cathodes, we used 20 wt% of Pt/C to reduce the cost. Furthermore, the spraying uniformity of Pt/C on Ti-based substrates was evaluated using laser-induced breakdown spectroscopy (LIBS). The LIBS mapping revealed that the deviation in LIBS intensity was about 7%, indicating spatial uniformity of the Pt amount was controlled to > 90% level (Fig. S23). Since the choice of a membrane is important as it directly impacts the efficiency, durability, and overall performance of the AEMWE [1, 3], we conducted an initial analysis using three commercial membranes such as PiperION, Fumasep FAA-3–50, and Sustainion® X37-50 grade RT (Fig. S22b). The assembled electrolyzer (NiFe/ATNT ǁ Pt/C/ATNT) was initially circulated with a 1.0 M KOH solution with a constant flow rate of 3 mL min^−1^ at an operating temperature of 60 ± 3 °C. The NiFe/ATNT ǁ Pt/C/ATNT with PiperION demonstrated the best performance (1.28 A cm^−2^) among Sustainion® X37-50 grade RT (0.86 A cm^−2^) and Fumasep FAA-3–50 (0.88 A cm^−2^) at 1.80 V (Fig. S24a). The assessment of AEMWE performance under different electrolyte flow rates and operating temperatures revealed that the optimal conditions for the NiFe/ATNT ǁ Pt/C/ATNT setup were a flow rate of 3 mL min^−1^ and an operating temperature of 80 ± 3 °C (Fig. S24b, c and see Supporting Information) [3, 57, 58]. At this optimal condition, the three membranes were tested again for performance comparison. Since the ionic conductivity of Fumasep FAA-3–50 (25–60 mS cm⁻^1^) is lower than that of PiperION (80–150 mS cm⁻^1^), NiFe/ATNT ‖ Pt/C/ATNT setup with Fumasep FAA-3–50 exhibited reduced performance (Fig. S24d) [3]. In contrast, the Sustainion® X37-50 grade RT membrane failed to operate at 80 ± 3 °C, possibly due to its limited thermal and alkaline stability [57, 58]. The superior performance of PiperION is attributed to its advanced polymer backbone and high-density functional groups, which enable stable and efficient hydroxide ion transport even at elevated temperatures.

Furthermore, to distinguish the impacts of super-hydrophilic and hydrophobic substrates on electrolyzer performance under optimal conditions, the performance of the NiFe/ATNT setup was compared with that of the NiFe/TF setup. As shown in Fig. 4b, the NiFe/TF ǁ Pt/C/TF configuration showed lower performance, with a current density of 0.71 A cm^−2^ at 1.80 V and 80 ± 3 °C. This was more than two times less than the NiFe/ATNT ǁ Pt/C/ATNT under the same conditions. To understand the impact of hydrophilicity and gas bubble accumulation on this improvement, comparative EIS analysis was conducted on these AEMWE setups at two voltage conditions: 1.40 V (no bubble) and 1.60 V (with bubble). As shown in Fig. 4c, at 1.40 V, the AEMWE with NiFe/ATNT exhibited lower system resistance (0.089 Ω cm^2^) than NiFe/TF (0.167 Ω cm^2^). Under the more challenging condition of 1.60 V, the system resistance for the AEMWE with NiFe/ATNT slightly increased to 0.092 Ω cm^2^, while the NiFe/TF configuration showed a significant increase to 0.244 Ω cm^2^. According to Ohm’s law, the lower internal resistance of AEMWE with ATNT electrodes enables a higher current density compared to AEMWE with TF, likely due to fewer dead zones, as depicted in the earlier section. On the other hand, the TF-supported AEMWE showed higher internal resistance due to the accumulation of gas bubbles, creating dead zones on the electrode surface. In addition, the AEMWE with the NiFe/ATNT electrode exhibited a lower Tafel slope (87 mV dec^−1^) from 100% *iR*-corrected LSV graphs compared to the NiFe/TF electrode (114 mV dec^−1^) (Fig. 4d). The NiFe/TF electrode also showed a significant deviation from the extrapolated Tafel line at higher current densities, while the NiFe/ATNT electrode exhibited only minimal deviation. To understand this, the cell overpotential was divided into mass-transfer overpotential (η_mass_), ohmic overpotential (η_ohm_), and kinetic overpotential (η_kin_) (see Supporting Information). At a low current density of 0.30 A cm^−2^, η_kin_ dominated the overall polarization (Fig. S25). The total overpotential for the NiFe/ATNT electrode was consistently lower than for the NiFe/TF electrode across all current densities. At higher currents (0.50 and 1.00 A cm^−2^), the NiFe/TF electrode showed a significant η_mass_ contribution, while the NiFe/ATNT electrode had a much lower η_mass_ (Fig. 4e). The small η_mass_ value of the NiFe/ATNT electrode is due to the fast gas bubble release, minimized dead zones, and improved active site exposure/reactant transport compared to NiFe/TF at high current densities compared to NiFe/TF electrode (Fig. 4f, g).

To determine whether the performance improvement is due to the substrate effect or the NiFe NPs, AEMWE experiments were also conducted using commercial catalysts, such as IrO_2_ and Ni powder, as the anode catalysts on both super-hydrophilic ATNT and hydrophobic TF substrates. In this analysis, LSV curves revealed that the IrO_2_/TF configuration had a lower current density of 0.73 A cm^−2^ at 1.80 V and 80 ± 3 °C, approximately 0.15 A cm^−2^ less than the IrO_2_/ATNT configuration (Fig. S26a). Upon comparing the EIS analysis at 1.40 and 1.60 V (Fig. S26b), IrO_2_/ATNT ǁ Pt/C/ATNT configuration surprisingly demonstrated lower system resistance (0.121 Ω cm^2^) compared to the IrO_2_/TF configuration (IrO_2_/TF ǁ Pt/C/TF) (0.152 Ω cm^2^) at 1.40 V. Moreover, at 1.60 V, the IrO_2_/ATNT configuration experienced a slight increase in resistance to 0.137 Ω cm^2^, while the IrO_2_/TF configuration showed a significant increase to 0.179 Ω cm^2^. A similar trend was also observed with commercial Ni powder as an anode catalyst on super-hydrophilic ATNT and hydrophobic TF substrates (Fig. S26c, d). We also performed additional tests using commercial Ni powder as the cathode catalyst on ATNT and TF, by replacing Pt/C. The results showed that the NiFe/ATNT ‖ Ni powder/ATNT setup achieved a current density of 0.77 A cm⁻^2^ at 1.80 V and 80 ± 3 °C (Fig. S26e). This setup outperformed the hydrophobic TF-based configuration (NiFe/TF ‖ Ni powder/TF) with the same catalysts. EIS analysis at 1.40 and 1.60 V (Fig. S26f) further demonstrated the advantages of the NiFe/ATNT ‖ Ni powder/ATNT setup, which had a lower system resistance of 0.153 Ω cm^2^ at 1.40 V compared to 0.170 Ω cm^2^ for the NiFe/TF ‖ Ni powder/TF configuration. At 1.60 V, the resistance of the Ni powder/ATNT-based setup increased slightly to 0.179 Ω cm^2^, whereas the Ni powder/TF-based setup showed a significant rise to 0.427 Ω cm^2^. These experiment results indicated that the super-hydrophilic ATNT substrate more effectively eliminates the formation of dead zones and substantially reduces electrolyzer resistance, enhancing overall performance.

**Fig. 5:**
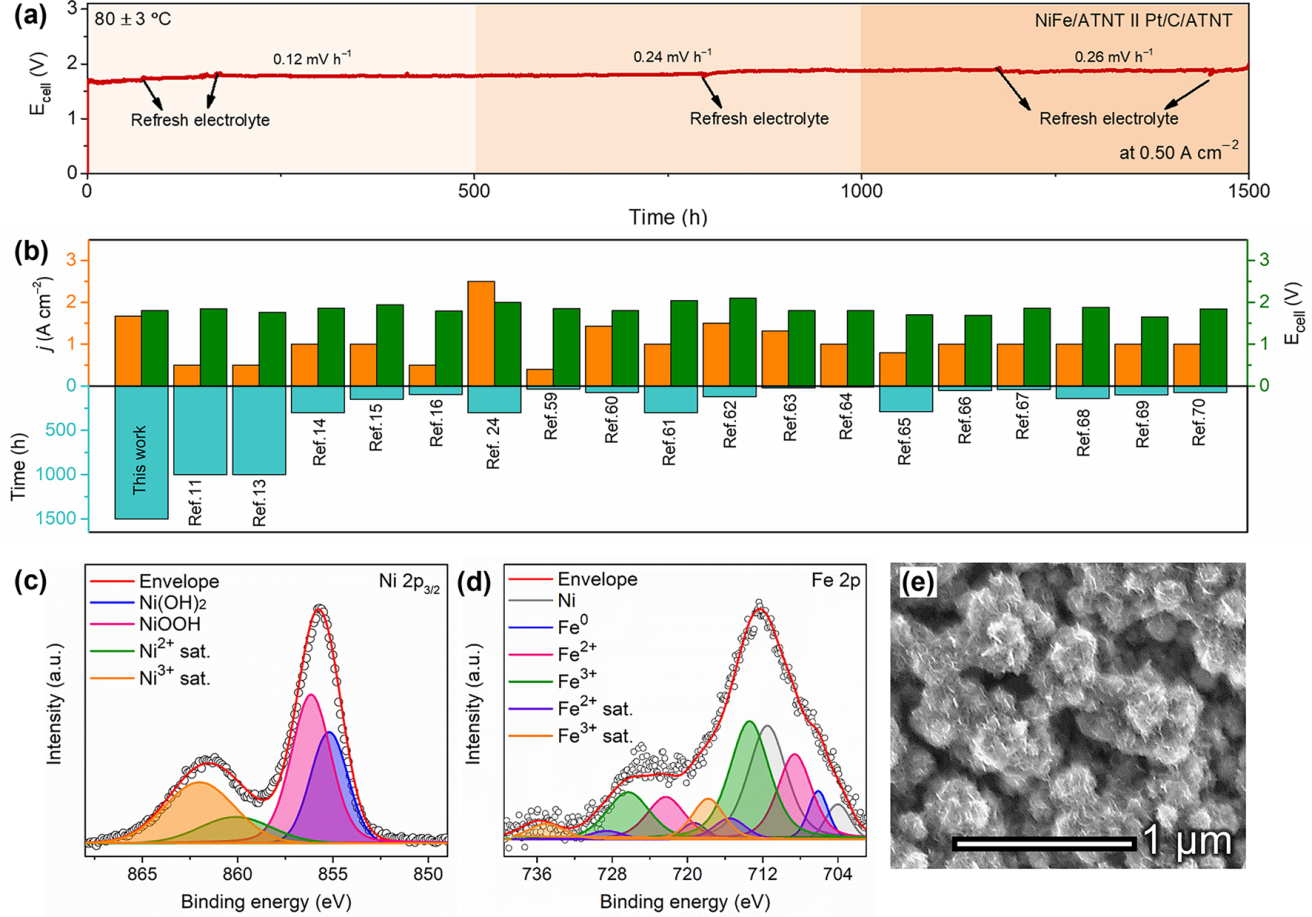
Long-term stability assessment of the prepared AEMWE. **a** Long-term stability of AEMWE at a constant current density of 0.50 A cm^−2^ for 1500 h at 80 ± 3 °C. **b** Comparative assessment of the current density (*j*), E_cell_, and stability of the prepared AEMWE in comparison with previously reported AEMWEs. XPS spectra on **c** Ni 2*p*_3/2_ and **d** Fe 2*p* regions, and **e** FESEM image of NiFe/ATNT after stability test

The long-term stability of the NiFe/ATNT ǁ Pt/C/ATNT was tested at a constant current density of 0.50 A cm^−2^ at 80 ± 3 °C for 1500 h (Fig. 5a). By comparing with other AEMWE using non-noble metals as anode, the performance and stability of the NiFe/ATNT ǁ Pt/C/ATNT surpassed prior results (Fig. 5b; Table S5) [11, 13–16, 24, 59–70] We used XPS and microscopic analyses to examine the chemical and morphological changes of the NiFe/ATNT after AEMWE stability test. The XPS peaks on Ti 2*p* region revealed that the ATNT substrate maintained a stable TiO_2_ phase under the anodic condition of AEMWE (Fig. S27). However, Ni 2*p*_3/2_ and Fe 2*p* regions mainly showed 3 + oxidation state, indicating the formation of OER active NiFeOOH phase during the stability test (Fig. 5c, d) [71, 72]. HRTEM and FESEM images of NiFe/ATNT after the AEMWE stability test also revealed that NiFe NPs fully transformed to NiFeOOH nanosheets (Figs. 5e and S28), well matched with XPS results [36, 72]. The electrolyzer showed an overall degradation rate of approximately 0.20 mV h⁻^1^ over the 1500 h stability test (Fig. 5a). The first 500 h exhibited a degradation rate of 0.12 mV h⁻^1^, which then increased to 0.24 mV h⁻^1^ during the next 500 h, and further rose to 0.26 mV h⁻^1^ in the final 500 h. These findings suggest that, although the NiFe/ATNT catalyst formed the active NiFeOOH phase during the stability test, gradual morphological changes in the catalyst surface likely resulted in a slow loss of contact between the membrane and the catalyst layer, contributing to the degradation of the electrolyzer over time. EIS analysis conducted at 1.60 V showed a slight increase in the resistance value of the electrolyzer from 0.092 to 0.126 Ω cm^2^ during the stability testing (Fig. S29). These post-test characterizations confirmed that the NiFe/ATNT is highly stable under harsh conditions. To evaluate the efficiency of the AEMWE system with the NiFe/ATNT ‖ Pt/C/ATNT setup, we conducted a cell efficiency test using 1.0 M KOH as the electrolyte at 0.50 A cm⁻^2^ for 60 s at 80 ± 3 °C, with a flow rate of 3 mL min⁻^1^ (Fig. S30). The results showed a cell efficiency of 79.47%, indicating the hydrophilic ATNT substrates as efficient supports for electrolyzer applications. We further calculated the energy consumption of the NiFe/ATNT ‖ Pt/C/ATNT setup under the same operational conditions using Eq. S7, yielding a value of 4.05 kWh Nm⁻^3^. For comparison, commercial electrolyzers typically consume 4.5—7.5 kWh Nm⁻^3^ for AWE and 5.8—7.5 kWh Nm⁻^3^ for PEMWE [73]. The lower energy consumption of the NiFe/ATNT ‖ Pt/C/ATNT setup indicates its superior performance than existing technologies. The energy efficiency of this setup based on energy consumption was found to be 87.4% with KOH using Eq. S8. These results demonstrate that our system is close to achieving the International Renewable Energy Agency‘s (IRENA) target of reducing energy consumption to below 4 kWh Nm⁻^3^ or 42 kWh kg⁻^1^ by 2050 [2].

Bubble transport in an electrolyzer is more complex than in a half-cell system due to the zero-gap configuration. To investigate this, we employed an advanced transparent electrolyzer to observe bubble behavior in both the NiFe/ATNT and NiFe/TF electrode setups (Fig. S31a; Video S2). At a constant current density of 0.2 A cm^−2^, the NiFe/TF ‖ Pt/C/TF setup (hydrophobic) exhibited large bubble formation and slow bubble release (Fig. S31b). In contrast, the NiFe/ATNT ‖ Pt/C/ATNT setup (super-hydrophilic) rapidly generated numerous small gas bubbles, facilitating efficient bubble evacuation from the electrolyzer (Fig. S31c). These findings indicated that the ATNT substrate facilitates gas bubble release and reduces dead zones compared to the TF substrate, and thus extends the lifespan of electrolyzer.

The pure water AEMWE performance was also evaluated with the NiFe/ATNT ‖ Pt/C/ATNT configuration, using DI water as the electrolyte under the same operating conditions as KOH. The results showed that the NiFe/ATNT ‖ Pt/C/ATNT system achieved a current density of 0.69 A cm⁻^2^ at 1.80 V (Fig. S32a) and exhibited good stability, operating continuously for 100 h at 0.2 A cm⁻^2^ with a degradation rate of 0.92 mV h⁻^1^ (Fig. S32b). The cell efficiency of the NiFe/ATNT ‖ Pt/C/ATNT setup was evaluated using DI water as the electrolyte at 0.50 A cm⁻^2^ for 60 s, with a flow rate of 3 mL min⁻^1^ and a temperature of 80 ± 3 °C, yielding an efficiency of 63.78% (Fig. S32c, d). We further evaluated the energy consumption of the NiFe/ATNT ‖ Pt/C/ATNT setup under the same conditions, obtaining a value of 5.03 kWh Nm⁻^3^. The corresponding energy consumption efficiency with DI water was determined to be 70.3%.

The dynamic operational durability of the AEMWE system with NiFe/ATNT ‖ Pt/C/ATNT configuration was evaluated by simulating start-stop water electrolysis operations in 1.0 M KOH, 0.1 M KOH, and DI water under different voltage conditions. The system underwent 100 square-wave cycles between 0.10 and 1.60 V and 50 cycles between 0.10 and 1.80 V at 80 ± 3 °C (Fig. S33a), with each voltage held for 1 min per cycle. In 1.0 M KOH, the current density exhibited only a minimal change of approximately 0.08 mA cm^−2^ per cycle compared to the first cycle (Fig. S33b). Even under more demanding conditions of 50 square-wave cycles between 0.10 and 1.80 V, the current density decreased by only 0.22 mA cm^−2^ per cycle (Fig. S33c, d). In 0.1 M KOH, the system showed good stability, with current density losses of 0.15 mA cm^−2^ per cycle over 100 cycles (0.10—1.60 V) and 0.34 mA cm^−2^ per cycle over 50 cycles (0.10—1.80 V) at 80 ± 3 °C. Under DI water conditions, the system also demonstrated good stability, with a current density loss of 0.27 mA cm^−2^ per cycle over 100 cycles (0.10—1.60 V) and 0.46 mA cm^−2^ per cycle over 50 cycles (0.10—1.80 V) at 80 ± 3 °C. The observed increase in current density loss in DI water is likely due to the lower ionic conductivity than alkaline electrolytes. These findings demonstrate the excellent stability of the NiFe/ATNT ‖ Pt/C/ATNT system across different electrolytes and dynamic cycling conditions.

## Conclusions

We have developed a super-hydrophilic and highly roughened ATNT as a catalyst substrate through anodizing and annealing treatment on TF. Subsequently, NiFe NPs were successfully electrodeposited onto the ATNT substrate with controlled surface morphology and composition. Electrocatalytic analysis revealed that the super-hydrophilic nature of the NiFe/ATNT enabled certain advantageous characteristics, such as OER overpotential of 235 mV at 10 mA cm^−2^, a smaller value of Tafel slope (60 mV dec^−1^), lower charge-transfer resistance, increased ECSA, and advanced stability compared to hydrophobic electrodes in this study. Under optimized conditions, AEMWE with NiFe/ATNT configuration showed more than twofold increase in current density (1.67 A cm^−2^ at 1.80 V) compared to the commercial IrO_2_/TF (0.73 A cm^−2^) and the NiFe/TF (0.71 A cm^−2^) on hydrophobic substrate configurations. In addition, AEMWE with NiFe/ATNT performed record stability over 1500 h at 0.50 A cm^−2^ under harsh condition of 80 ± 3 °C. The results are attributed to the distinctive properties of NiFe/ATNT, including super-hydrophilicity combined with effective gas evacuation, porous NiFe NPs structure, and modified electronic structure. We believe that this study opens up new opportunities for researchers developing AEMWE systems by providing insights into the interplay between surface properties and electrochemical performance.

## Supplementary Information

Below is the link to the electronic supplementary material.

Supplementary file1 (MP4 767 KB)

Supplementary file2 (MP4 1313 KB)

Supplementary file3 (DOCX 31517 KB)

## References

[1] C. Hu, Y. Wang, Y.M. Lee, Ether-free alkaline polyelectrolytes for water electrolyzers: recent advances and perspectives. Angew. Chem. Int. Ed. **64**, e202418324 (2025). 10.1002/anie.202418324 10.1002/anie.202418324 39485307

[2] S. Shaik, J. Kundu, Y. Yuan, W. Chung, D. Han et al., Recent progress and perspective in pure water-fed anion exchange membrane water electrolyzers. Adv. Energy Mater. **14**, 2470148 (2024). 10.1002/aenm.202470148

[3] Q. Xu, L. Zhang, J. Zhang, J. Wang, Y. Hu et al., Anion exchange membrane water electrolyzer: electrode design, lab-scaled testing system and performance evaluation. EnergyChem **4**, 100087 (2022). 10.1016/j.enchem.2022.100087

[4] J. Ding, Z. Peng, Z. Wang, C. Zeng, Y. Feng et al., Phosphorus–tungsten dual-doping boosts acidic overall seawater splitting performance over RuO_*x*_ nanocrystals. J. Mater. Chem. A **12**, 28023–28031 (2024). 10.1039/D4TA05277C

[5] W. Liu, W. Liu, T. Hou, J. Ding, Z. Wang et al., Coupling Co–Ni phosphides for energy-saving alkaline seawater splitting. Nano Res. **17**, 4797–4806 (2024). 10.1007/s12274-024-6433-8

[6] D. Zha, R. Wang, S. Tian, Z.-J. Jiang, Z. Xu et al., Defect engineering and carbon supporting to achieve Ni-doped CoP_3_ with high catalytic activities for overall water splitting. Nano-Micro Lett. **16**, 250 (2024). 10.1007/s40820-024-01471-9 10.1007/s40820-024-01471-9 PMC11258119 39023812

[7] Y. Liu, P. Vijayakumar, Q. Liu, T. Sakthivel, F. Chen et al., Shining light on anion-mixed nanocatalysts for efficient water electrolysis: fundamentals, progress, and perspectives. Nano-Micro Lett. **14**, 43 (2022). 10.1007/s40820-021-00785-2 10.1007/s40820-021-00785-2 PMC8724338 34981288

[8] X. Gao, Y. Chen, Y. Wang, L. Zhao, X. Zhao et al., Next-generation green hydrogen: progress and perspective from electricity, catalyst to electrolyte in electrocatalytic water splitting. Nano-Micro Lett. **16**, 237 (2024). 10.1007/s40820-024-01424-2 10.1007/s40820-024-01424-2 PMC11226619 38967856

[9] X. Gao, S. Dai, Y. Teng, Q. Wang, Z. Zhang et al., Ultra-efficient and cost-effective platinum nanomembrane electrocatalyst for sustainable hydrogen production. Nano-Micro Lett. **16**, 108 (2024). 10.1007/s40820-024-01324-5 10.1007/s40820-024-01324-5 PMC10844191 38315294

[10] X. Wang, W. Pi, S. Hu, H. Bao, N. Yao et al., Boosting oxygen evolution reaction performance on NiFe-based catalysts through d-orbital hybridization. Nano-Micro Lett. **17**, 11 (2024). 10.1007/s40820-024-01528-9 10.1007/s40820-024-01528-9 PMC11427650 39325091

[11] X. Jiang, V. Kyriakou, B. Wang, S. Deng, S. Costil et al., Hierarchical microporous Ni-based electrodes enable “two birds with one stone” in highly efficient and robust anion exchange membrane water electrolysis (AEMWE). Chem. Eng. J. **486**, 150180 (2024). 10.1016/j.cej.2024.150180

[12] M.K. Kabiraz, J. Kim, H.J. Lee, S. Park, Y.W. Lee et al., Nickel nanoplates enclosed by (111) facets as durable oxygen evolution catalysts in anion exchange membrane water electrolyzers. Adv. Funct. Mater. **34**, 2406175 (2024). 10.1002/adfm.202406175

[13] Z. Liang, D. Shen, Y. Wei, F. Sun, Y. Xie et al., Modulating the electronic structure of cobalt-vanadium bimetal catalysts for high-stable anion exchange membrane water electrolyzer. Adv. Mater. **36**, e2408634 (2024). 10.1002/adma.202408634 39148167 10.1002/adma.202408634

[14] Y. Shi, L. Song, Y. Liu, T. Wang, C. Li et al., Dual cocatalytic sites synergize NiFe layered double hydroxide to boost oxygen evolution reaction in anion exchange membrane water electrolyzer. Adv. Energy Mater. **14**, 2402046 (2024). 10.1002/aenm.202402046

[15] F. Malaj, A. Tampucci, D. Lentini, L. Brogi, E. Berretti et al., One-pot synthesis of FeNi_3_/FeNiO_*x*_ nanoparticles for PGM-free anion exchange membrane water electrolysis. Electrochim. Acta **507**, 145109 (2024). 10.1016/j.electacta.2024.145109

[16] L. Wu, M. Ning, X. Xing, Y. Wang, F. Zhang et al., Boosting oxygen evolution reaction of (Fe, Ni)OOH *via* defect engineering for anion exchange membrane water electrolysis under industrial conditions. Adv. Mater. **35**, e2306097 (2023). 10.1002/adma.202306097 37607336 10.1002/adma.202306097

[17] G. Luo, M. Song, Q. Zhang, L. An, T. Shen et al., Advances of synergistic electrocatalysis between single atoms and nanoparticles/clusters. Nano-Micro Lett. **16**, 241 (2024). 10.1007/s40820-024-01463-9 10.1007/s40820-024-01463-9 PMC11233490 38980634

[18] N. Han, W. Zhang, W. Guo, H. Pan, B. Jiang et al., Designing oxide catalysts for oxygen electrocatalysis: insights from mechanism to application. Nano-Micro Lett. **15**, 185 (2023). 10.1007/s40820-023-01152-z 10.1007/s40820-023-01152-z PMC10387042 37515746

[19] C. Wang, Q. Zhang, B. Yan, B. You, J. Zheng et al., Facet engineering of advanced electrocatalysts toward hydrogen/oxygen evolution reactions. Nano-Micro Lett. **15**, 52 (2023). 10.1007/s40820-023-01024-6 10.1007/s40820-023-01024-6 PMC9935811 36795218

[20] P. Wang, Y. Luo, G. Zhang, Z. Chen, H. Ranganathan et al., Interface engineering of Ni_x_S_y_@MnO_x_H_y_ nanorods to efficiently enhance overall-water-splitting activity and stability. Nano-Micro Lett. **14**, 120 (2022). 10.1007/s40820-022-00860-2 10.1007/s40820-022-00860-2 PMC9065220 35505126

[21] Y. Li, Y. Li, H. Sun, L. Gao, X. Jin et al., Current status and perspectives of dual-atom catalysts towards sustainable energy utilization. Nano-Micro Lett. **16**, 139 (2024). 10.1007/s40820-024-01347-y 10.1007/s40820-024-01347-y PMC10904713 38421549

[22] A. Angulo, P. van der Linde, H. Gardeniers, M. Modestino, D.F. Rivas, Influence of bubbles on the energy conversion efficiency of electrochemical reactors. Joule **4**(3), 555–579 (2020). 10.1016/j.joule.2020.01.005

[23] F. Razmjooei, T. Morawietz, E. Taghizadeh, E. Hadjixenophontos, L. Mues et al., Increasing the performance of an anion-exchange membrane electrolyzer operating in pure water with a nickel-based microporous layer. Joule **5**, 1776–1799 (2021). 10.1016/j.joule.2021.05.006

[24] L. Wan, Z. Xu, P. Wang, P.-F. Liu, Q. Xu et al., Dual regulation both intrinsic activity and mass transport for self-supported electrodes using in anion exchange membrane water electrolysis. Chem. Eng. J. **431**, 133942 (2022). 10.1016/j.cej.2021.133942

[25] K. Dastafkan, S. Wang, S. Song, Q. Meyer, Q. Zhang et al., *operando* monitoring of gas bubble evolution in water electrolysis by single high-frequency impedance. EES. Catal. **1**, 998–1008 (2023). 10.1039/d3ey00182b

[26] Z. Chen, S. Yun, L. Wu, J. Zhang, X. Shi et al., Waste-derived catalysts for water electrolysis: circular economy-driven sustainable green hydrogen energy. Nano-Micro Lett. **15**, 4 (2022). 10.1007/s40820-022-00974-7 10.1007/s40820-022-00974-7 PMC9715911 36454315

[27] M. Bellini, E. Berretti, M. Innocenti, G. Magherini, M.V. Pagliaro et al., 3D titania nanotube array support for water electrolysis palladium catalysts. Electrochim. Acta **383**, 138338 (2021). 10.1016/j.electacta.2021.138338

[28] J. Wang, J. Tian, W. Wang, Z. Zhao, L. Li et al., Enhanced photocatalytic activity of graphene/TiO_2_ nanotubes composites prepared by wet transfer method. Fuller. Nanotub. Carbon Nanostruct. **30**, 495–502 (2022). 10.1080/1536383X.2021.1960510

[29] Y. Xiao, Z. Wang, M. Li, Q. Liu, X. Liu et al., Efficient charge separation in Ag/PCN/UPDI ternary heterojunction for optimized photothermal-photocatalytic performance *via* tandem electric fields. Small **20**, e2306692 (2024). 10.1002/smll.202306692 38773907 10.1002/smll.202306692

[30] F. He, Y. Liu, X. Yang, Y. Chen, C.-C. Yang et al., Accelerating oxygen electrocatalysis kinetics on metal-organic frameworks *via* bond length optimization. Nano-Micro Lett. **16**, 175 (2024). 10.1007/s40820-024-01382-9 10.1007/s40820-024-01382-9 PMC11031554 38639824

[31] A. Zabilska, A.H. Clark, B.M. Moskowitz, I.E. Wachs, Y. Kakiuchi et al., Redox dynamics of active VO_*x*_ sites promoted by TiO_*x*_ during oxidative dehydrogenation of ethanol detected by *operando* quick XAS. JACS Au **2**, 762–776 (2022). 10.1021/jacsau.2c00027 35388376 10.1021/jacsau.2c00027 PMC8977985

[32] G. Rossi, M. Calizzi, V. Di Cintio, S. Magkos, L. Amidani et al., Local structure of V dopants in TiO_2_ nanoparticles: X-ray absorption spectroscopy, including ab-initio and full potential simulations. J. Phys. Chem. C **120**, 7457–7466 (2016). 10.1021/acs.jpcc.5b12045

[33] J. He, W. Liu, Z. Hu, X. Wang, J. Liu et al., Well-dispersed CsPbBr_3_@TiO_2_ heterostructure nanocrystals from asymmetric to symmetric. Small **20**, e2406783 (2024). 10.1002/smll.202406783 39206610 10.1002/smll.202406783

[34] S.H. Ahn, A. Manthiram, Single Ni atoms and clusters embedded in N-doped carbon “tubes on fibers” matrix with bifunctional activity for water splitting at high current densities. Small **16**, 2002511 (2020). 10.1002/smll.202002511 10.1002/smll.202002511 33439543

[35] D. Peng, C. Hu, X. Luo, J. Huang, Y. Ding et al., Electrochemical reconstruction of NiFe/NiFeOOH superparamagnetic core/catalytic shell heterostructure for magnetic heating enhancement of oxygen evolution reaction. Small **19**, e2205665 (2023). 10.1002/smll.202205665 36404111 10.1002/smll.202205665

[36] R. Li, L. Gao, Z. Dou, L. Cui, Obtaining the high valence of Ni/Fe sites in a heterostructure induced by implanting the NiFe-DTO MOF as a highly active OER catalyst. ACS Sustain. Chem. Eng. **12**, 17761–17769 (2024). 10.1021/acssuschemeng.4c06643

[37] M. Bele, P. Jovanovič, Ž Marinko, S. Drev, V.S. Šelih et al., Increasing the oxygen-evolution reaction performance of nanotubular titanium oxynitride-supported Ir nanoparticles by a strong metal–support interaction. ACS Catal. **10**, 13688–13700 (2020). 10.1021/acscatal.0c03688

[38] D. Kim, X. Qin, B. Yan, Y. Piao, Sprout-shaped Mo-doped CoP with maximized hydrophilicity and gas bubble release for high-performance water splitting catalyst. Chem. Eng. J. **408**, 127331 (2021). 10.1016/j.cej.2020.127331

[39] H.B. Michaelson, The work function of the elements and its periodicity. J. Appl. Phys. **48**, 4729–4733 (1977). 10.1063/1.323539

[40] V. Mansfeldova, M. Zlamalova, H. Tarabkova, P. Janda, M. Vorokhta et al., Work function of TiO_2_ (anatase, rutile, and brookite) single crystals: effects of the environment. J. Phys. Chem. C **125**, 1902–1912 (2021). 10.1021/acs.jpcc.0c10519

[41] E. Hua, S. Choi, S. Ren, S. Kim, G. Ali et al., Negatively charged platinum nanoparticles on dititanium oxide electride for ultra-durable electrocatalytic oxygen reduction. Energy Environ. Sci. **16**, 4464–4473 (2023). 10.1039/D3EE01211E

[42] S.H. Baker, A.M. Asaduzzaman, M. Roy, S.J. Gurman, C. Binns et al., Atomic structure and magnetic moments in cluster-assembled nanocomposite Fe/Cu films. Phys. Rev. B **78**, 014422 (2008). 10.1103/physrevb.78.014422

[43] Y. Lin, L. Zhao, L. Wang, Y. Gong, Ruthenium-doped NiFe-based metal–organic framework nanoparticles as highly efficient catalysts for the oxygen evolution reaction. Dalton Trans. **50**, 4280–4287 (2021). 10.1039/D0DT04133E 33688870 10.1039/d0dt04133e

[44] B.-A. Mei, J. Lau, T. Lin, S.H. Tolbert, B.S. Dunn et al., Physical interpretations of electrochemical impedance spectroscopy of redox active electrodes for electrical energy storage. J. Phys. Chem. C **122**, 24499–24511 (2018). 10.1021/acs.jpcc.8b05241

[45] S. Seenivasan, H. Moon, D.-H. Kim, Multilayer strategy for photoelectrochemical hydrogen generation: new electrode architecture that alleviates multiple bottlenecks. Nano-Micro Lett. **14**, 78 (2022). 10.1007/s40820-022-00822-8 10.1007/s40820-022-00822-8 PMC8956779 35334000

[46] Y. Hu, C. Wang, Electrodeposition of nickel–cobalt alloys from metal chloride-l-serine deep eutectic solvent for the hydrogen evolution reaction. J. Mater. Chem. A **12**, 16769–16779 (2024). 10.1039/D4TA02433H

[47] R.K. Dharman, H. Im, M.K. Kabiraz, J. Kim, K.P. Shejale et al., Stable 1T-MoS_2_ by facile phase transition synthesis for efficient electrocatalytic oxygen evolution reaction. Small Meth. **8**, 2301251 (2024). 10.1002/smtd.202301251 10.1002/smtd.202301251 38308408

[48] Y.J. Park, J. Lee, Y.S. Park, J. Yang, M.J. Jang et al., Electrodeposition of high-surface-area IrO_2_ films on Ti felt as an efficient catalyst for the oxygen evolution reaction. Front. Chem. **8**, 593272 (2020). 10.3389/fchem.2020.593272 33195098 10.3389/fchem.2020.593272 PMC7645052

[49] S.S. Jeon, P.W. Kang, M. Klingenhof, H. Lee, F. Dionigi et al., Active surface area and intrinsic catalytic oxygen evolution reactivity of NiFe LDH at reactive electrode potentials using capacitances. ACS Catal. **13**, 1186–1196 (2023). 10.1021/acscatal.2c04452

[50] B. Kim, M.K. Kabiraz, J. Lee, C. Choi, H. Baik et al., Vertical-crystalline Fe-doped β-Ni oxyhydroxides for highly active and stable oxygen evolution reaction. Matter **4**, 3585–3604 (2021). 10.1016/j.matt.2021.09.003

[51] H. Sun, C.W. Tung, Y. Qiu, W. Zhang, Q. Wang et al., Atomic metal-support interaction enables reconstruction-free dual-site electrocatalyst. J. Am. Chem. Soc. **144**, 1174–1186 (2022). 10.1021/jacs.1c08890 34935380 10.1021/jacs.1c08890

[52] G. Qian, J. Chen, T. Yu, J. Liu, L. Luo et al., Three-phase heterojunction NiMo-based nano-needle for water splitting at industrial alkaline condition. Nano-Micro Lett. **14**, 20 (2021). 10.1007/s40820-021-00744-x 10.1007/s40820-021-00744-x PMC8660933 34882293

[53] L. Pan, J. Sun, H. Qi, M. Han, Q. Dai et al., Dead-zone-compensated design as general method of flow field optimization for redox flow batteries. Proc. Natl. Acad. Sci. U.S.A. **120**, e2305572120 (2023). 10.1073/pnas.2305572120 37669368 10.1073/pnas.2305572120 PMC10500283

[54] R. Andaveh, G. Barati Darband, M. Maleki, A. Sabour Rouhaghdam, Superaerophobic/superhydrophilic surfaces as advanced electrocatalysts for the hydrogen evolution reaction: a comprehensive review. J. Mater. Chem. A **10**, 5147–5173 (2022). 10.1039/D1TA10519A

[55] A.R. Zeradjanin, P. Narangoda, I. Spanos, J. Masa, R. Schlögl, How to minimise destabilising effect of gas bubbles on water splitting electrocatalysts? Curr. Opin. Electrochem. **30**, 100797 (2021). 10.1016/j.coelec.2021.100797

[56] I. Bakonyi, Accounting for the resistivity contribution of grain boundaries in metals: critical analysis of reported experimental and theoretical data for Ni and Cu. Eur. Phys. J. Plus **136**, 410 (2021). 10.1140/epjp/s13360-021-01303-4

[57] G.H.A. Wijaya, K.S. Im, S.Y. Nam, Advancements in commercial anion exchange membranes: a review of membrane properties in water electrolysis applications. Desalin. Water Treat. **320**, 100605 (2024). 10.1016/j.dwt.2024.100605

[58] G.A. Lindquist, S.Z. Oener, R. Krivina, A.R. Motz, A. Keane et al., Performance and durability of pure-water-fed anion exchange membrane electrolyzers using baseline materials and operation. ACS Appl. Mater. Interfaces **13**, 51917–51924 (2021). 10.1021/acsami.1c06053 34374278 10.1021/acsami.1c06053

[59] D. Chanda, K. Kannan, J. Gautam, M.M. Meshesha, S.G. Jang et al., Effect of the interfacial electronic coupling of nickel-iron sulfide nanosheets with layer Ti_3_C_2_ MXenes as efficient bifunctional electrocatalysts for anion-exchange membrane water electrolysis. Appl. Catal. B Environ. **321**, 122039 (2023). 10.1016/j.apcatb.2022.122039

[60] A. Martinez-Lazaro, A. Caprì, I. Gatto, J. Ledesma-García, N. Rey-Raap et al., NiFe_2_O_4_ hierarchical nanoparticles as electrocatalyst for anion exchange membrane water electrolysis. J. Power Sources **556**, 232417 (2023). 10.1016/j.jpowsour.2022.232417

[61] X. Lin, X. Li, L. Shi, F. Ye, F. Liu et al., *In situ* electrochemical restructuring B-doped metal-organic frameworks as efficient OER electrocatalysts for stable anion exchange membrane water electrolysis. Small **20**, e2308517 (2024). 10.1002/smll.202308517 38155580 10.1002/smll.202308517

[62] S. Li, T. Liu, W. Zhang, M. Wang, H. Zhang et al., Highly efficient anion exchange membrane water electrolyzers *via* chromium-doped amorphous electrocatalysts. Nat. Commun. **15**, 3416 (2024). 10.1038/s41467-024-47736-0 38649713 10.1038/s41467-024-47736-0 PMC11035637

[63] J. Lee, H. Jung, Y.S. Park, S. Woo, N. Kwon et al., Corrosion-engineered bimetallic oxide electrode as anode for high-efficiency anion exchange membrane water electrolyzer. Chem. Eng. J. **420**, 127670 (2021). 10.1016/j.cej.2020.127670

[64] Y.S. Park, M.J. Jang, J. Jeong, S.M. Park, X. Wang et al., Hierarchical chestnut-burr like structure of copper cobalt oxide electrocatalyst directly grown on Ni foam for anion exchange membrane water electrolysis. ACS Sustain. Chem. Eng. **8**, 2344–2349 (2020). 10.1021/acssuschemeng.9b06767

[65] S. Kang, K. Ham, J. Lee, Moderate oxophilic CoFe in carbon nanofiber for the oxygen evolution reaction in anion exchange membrane water electrolysis. Electrochim. Acta **353**, 136521 (2020). 10.1016/j.electacta.2020.136521

[66] S.S. Jeon, J. Lim, P.W. Kang, J.W. Lee, G. Kang et al., Design principles of NiFe-layered double hydroxide anode catalysts for anion exchange membrane water electrolyzers. ACS Appl. Mater. Interfaces **13**, 37179–37186 (2021). 10.1021/acsami.1c09606 34251792 10.1021/acsami.1c09606

[67] S.C. Karthikeyan, S. Ramakrishnan, S. Prabhakaran, M.R. Subramaniam, M. Mamlouk et al., Low-cost self-reconstructed high entropy oxide as an ultra-durable OER electrocatalyst for anion exchange membrane water electrolyzer. Small **20**, 2402241 (2024). 10.1002/smll.202402241 10.1002/smll.202402241 39082423

[68] F.-L. Wang, N. Xu, C.-J. Yu, J.-Y. Xie, B. Dong et al., Porous heterojunction of Ni_2_P/Ni_7_S_6_ with high crystalline phase and superior conductivity for industrial anion exchange membrane water electrolysis. Appl. Catal. B Environ. **330**, 122633 (2023). 10.1016/j.apcatb.2023.122633

[69] G. Ding, H. Lee, Z. Li, J. Du, L. Wang et al., Highly efficient and durable anion exchange membrane water electrolyzer enabled by a Fe–Ni_3_S_2_ anode catalyst. Adv. Energy Sustain. Res. **4**, 2200130 (2023). 10.1002/aesr.202200130

[70] A. Meena, P. Thangavel, D.S. Jeong, A.N. Singh, A. Jana et al., Crystalline-amorphous interface of mesoporous Ni_2_P@ FePO_x_H_y_ for oxygen evolution at high current density in alkaline-anion-exchange-membrane water-electrolyzer. Appl. Catal. B Environ. **306**, 121127 (2022). 10.1016/j.apcatb.2022.121127

[71] Q. Kang, D. Lai, W. Tang, Q. Lu, F. Gao, Intrinsic activity modulation and structural design of NiFe alloy catalysts for an efficient oxygen evolution reaction. Chem. Sci. **12**, 3818–3835 (2021). 10.1039/d0sc06716d 34163652 10.1039/d0sc06716d PMC8179442

[72] C. Lei, K. Yang, G. Wang, G. Wang, J. Lu et al., Impact of catalyst reconstruction on the durability of anion exchange membrane water electrolysis. ACS Sustain. Chem. Eng. **10**, 16725–16733 (2022). 10.1021/acssuschemeng.2c04855

[73] M. El-Shafie, Hydrogen production by water electrolysis technologies: a review. Results Eng. **20**, 101426 (2023). 10.1016/j.rineng.2023.101426

