# Supplementary material for: Rapid Outgassing of Hydrophilic TiO2 Electrodes Achieves Long-Term Stability of Anion Exchange Membrane Water Electrolyzers
Source: Nanomicro Lett. 2025 Mar 13;17:186. doi: 10.1007/s40820-025-01696-2 (PMC11906940; doi:10.1007/s40820-025-01696-2)
Supplement: Supplementary file 3 — Supplementary file3 (DOCX 31517 KB) [file 40820_2025_1696_MOESM3_ESM.docx]

Supporting Information for

**Rapid Outgassing of Hydrophilic TiO_2_ Electrodes Achieves Long-Term Stability of Anion Exchange Membrane Water Electrolyzers**

Shajahan Shaik^1,6^, Jeonghyeon Kim^1,6^, Mrinal Kanti Kabiraz^1^, Faraz Aziz^2^, Joon Yong Park^3^, Bhargavi Rani Anne^4^, Mengfan Li^5^, Hongwen Huang^5^, Ki Min Nam^3^, Daeseong Jo^2^, and Sang-Il Choi^1,^*

^1^Department of Chemistry and Green-Nano Materials Research Center, Kyungpook National University, Daegu 41566, South Korea

^2^Department of Mechanical Engineering, Kyungpook National University, Daegu 41566, South Korea

^3^Department of Chemistry and Chemistry Institute for Functional Materials, Pusan National University, Busan 46241, South Korea

^4^Department of Metallurgical and Materials Engineering, National Institute of Technology, Raipur 492010, India

^5^College of Materials Science and Engineering, Hunan University, Changsha, Hunan 410082, P. R. China

^6^ Shajahan Shaik and Jeonghyeon Kim contributed equally to this work.

*Corresponding author. E-mail: [sichoi@knu.ac.kr](mailto:sichoi@knu.ac.kr) (Sang-Il Choi)

**S1 Supplementary Experimental Section**

**S1.1 Materials**

Nickel(II) sulfate hexahydrate (NiSO_4_·6H_2_O, ≥95%), iron(II) sulfate heptahydrate (FeSO_4_·7H_2_O, ≥95%), trisodium citrate dihydrate (Na_3_C_6_H_5_O_7_·2H_2_O, ≥95%), sodium bromide (NaBr, ≥95%), ammonium chloride (NH_4_Cl, ≥95%), ammonium fluoride (NH_4_F, ≥95%), ethylene glycol (C_2_H_6_O_2_, ≥95%), 5 wt% Nafion resin solution, and isopropyl alcohol (>99.5%) were purchased from Sigma Aldrich. Potassium hydroxide (KOH, 99.98%), commercial Pt/C (20 wt%), and commercial IrO_2_ (99%) were purchased from Alfa Aesar. Commercial Ni nanopowder (≥99.9%, 40 nm) was purchased from US Research Nanomaterials, Inc. Ethanol and acetone were purchased from Duksan. PiperION anion exchange dispersion (5 wt%) and Fumion dispersion (FAA-3-SOLUT-10) were purchased from Fuel Cell Store. Sustainion® XB-7 5% in ethanol was purchased from Dioxide Material. Platinized titanium screen mesh was purchased from Fuel Cell Store. Titanium felt (TF) was purchased from Bekaert.

**S1.2 Electrodeposition of NiFe Nanoparticles onto the TiO_2_ Layer Formed TF (NiFe/TOTF)**

The TF was first cleaned in acetone with ultrasonic treatment for 15 min, followed by multiple rinses with DI water. It was then dried in an oven at 60 °C for 20 min. Next, the TF was annealed in air at 600 °C for 15 h, forming a TOTF. NiFe was subsequently deposited onto the TOTF with a 4:1 molar ratio of Ni:Fe. After electrodeposition, the catalyst-coated electrode was thoroughly cleaned with ethanol and DI water and dried in an oven at 60 °C for 30 min.

**S1.3 Characterization**

Surface morphology of the developed catalysts was examined by field emission scanning electron microscopy (FESEM, Hitachi, S-4800) at the Korea Basic Science Institute (KBSI, Dague). To prepare the high-resolution transmission electron microscopy (HRTEM) samples, we employed a dual-beam focused ion beam (FIB) system (Hitachi, NX5000). TEM and high-angle annular dark field scanning TEM (HAADF-STEM) studies were performed in 300 kV Tecnai G2 F30 S-Twin and FEI Double Cs-corrected Titan ThemisZ 30-300 S/TEM instrument with next-generation spherical aberration correction technology. The surface topography of the samples was investigated by atomic force microscopy (AFM, Park Systems, NX20) with non-contact mode. To study the chemical structures of the samples, X-ray photoelectron spectroscopy (XPS, ThermoFisher, Scientific ESCALAB 250 Xi) was measured at the National Nanofab Center (NNFC, Daejeon) with Al Kα X-ray (1486.6 eV) as a light source. Synchrotron X-ray diffraction (SXRD) studies with synchronized X-ray were conducted at 8D beamline (XRS POSCO, λ = 1.54 Å) with 3.0 GeV storage ring energy and 300 mA operating current at Pohang accelerator laboratory (PAL) in the Republic of Korea. X-ray adsorption fine structure (XAFS) was performed using 7D-XAFS and 10C-Wide XAFS beamlines with 3.0 GeV storage ring energy and 300 mA operating current at PAL. All XAFS signals were recorded with a fluorescence mode using a PIPS detector. The obtained spectra were processed using the ATHENA software in IFFEFIT package. A wettability test was performed on the surface of electrodes before and after the catalyst coating using a drop of DI water to study the hydrophilic property of the electrodes. The water contact angle during the test was determined by Contact angle measurer (SEO, Phoenix) at ambient temperature. To obtain better accuracy, five different positions of each electrode surface were taken and the average values were reported. The molar ratio/composition of catalysts was determined by ICP-AES (Perkin-Elmer, Optima 700DV). A high-definition camera (MIRO EX 4), capable of capturing 1000 frames per second, was employed to record the formation and releasing of gas bubbles during an in-situ OER half-cell test. The electrical resistivity of the electrode was determined by the four-point probe resistivity method (AIT, CMT2000N). The laser-induced breakdown spectroscopy (LIBS) analysis was performed in the J200 instrument with a 265 nm Nd:YAG nanosecond laser and 6-channel enhanced EC detector, covering a spectral window of 190-1040 nm (Applied Spectra).

**S1.4 Electrochemical Active Surface Area (ECSA) Measurements**

To determine the active surface area of each catalyst, the double layer capacitance (C_dl_) was measured in the non-faradic region (0.52-0.62 V vs reversible hydrogen electrode, V_RHE_) of the cyclic voltammetry (CV) with different scan rates of 20, 40, 60, 80, 100, 120, 140, 160, 180, and 200 mV s^−1^. Then, plotted the scan rate versus the double-layer charge current density at 0.57 V_RHE_ yields a linear slope equivalent to the C_dl_ value. ECSA is obtained by dividing C_dl_ by the specific capacitance of a reference electrode. Mostly, the specific capacitance (C_s_) of a flat surface is reported to be in the range of 20-60 μF cm^−2^. Therefore, we assumed C_s_ to be 40 μF cm^−2^ and used it for the calculation.

$ECSA= \frac{C_{dl}(mF)}{C_{s}(mF \mathrm{cm}^{-2})}$ (S1)

**S1.5 *iR*-Correction**

The applied potentials were 100% *iR-*corrected by measuring the ohmic resistance (R) from the high-frequency region of the Nyquist plot. This plot was obtained from electrochemical impedance spectroscopy (EIS) analysis performed at 1.52 V_RHE_, with an amplitude of 5 mV over a frequency range of 100 kHz to 0.1 Hz. The corrected potential was determined using the following equation.

$V_{corr}=V_{meas}-iR$ (S2)

Where, *V_corr_* represents the corrected voltage, *V_meas_* represents the measured voltage, and *i* is the current.

**S1.6 Pre-Treatment of PiperION Membrane**

The PiperION membranes were initially obtained in bromide form. To transform the membrane into the OH^−^ form, it was immersed in a solution of 1.0 M KOH at room temperature for 1 h. Subsequently, the solution was replaced with fresh 1.0 M KOH, and the membrane was allowed to soak for an additional 1 h at room temperature. Following the two soaking steps, the membrane was rinsed with DI water to achieve a pH of ~7. To prevent the membrane from reverting back to the bicarbonate form, precautions were taken to minimize its exposure to ambient air by conducting the membrane pre-treatment in a CO_2_-free dry box environment.

**S1.7 Pre-Treatment of Fumasep FAA-3-50 Membrane**

To activate the Fumasep FAA-3-50 membrane, it was soaked in an aqueous solution of 1.0 M KOH at 24 °C for 24 h to remove other unwanted additives. Following the soaking process, the membrane was cleaned several times with DI water.

**S1.8 Pre-Treatment of Sustainion**® **X37-50 grade RT Membrane**

The Sustainion® X37-50 grade RT membrane was activated and converted to hydroxide form by soaking the corresponding membrane in an aqueous solution of 1.0 M KOH at 24 °C for 48 h. Before the experiment, the soaked membrane was washed with DI water several times. CO_2_ exposure to ambient air was minimized by conducting the membrane pre-treatment in a CO_2_-free dry box environment.

**S1.9 Preparation of IrO_2_/ATNT and Ni powder/ATNT Electrodes for Anion-Exchange Membrane Water Electrolyzer (AEMWE)**

The IrO_2_/ATNT electrode was prepared by a spray coating method. Catalyst ink was prepared by mixing 27 mg of commercial IrO_2_ (99%) catalyst powder, 200 µL of the respective commercially available ionomer for each membrane, 0.2 mL of DI water, and 0.8 mL of isopropyl alcohol. The mixture was then homogeneously sonicated for 60 min. Finally, the prepared catalyst ink was uniformly sprayed onto the ATNT substrate (3 cm × 3 cm) using a spray gun, with a catalyst loading of 3 mg cm^−2^. Then it was hot-pressed at 60 °C and 4 MPa for 10 s to ensure proper catalyst adhesion to the support before being incorporated into the membrane electrode assembly (MEA). The Ni powder/ATNT electrode was prepared following the same procedure as the IrO₂/ATNT electrode, with a catalyst loading of 5 mg cm^−2^. This catalyst was used as the anode in one experiment and as the cathode in another, both with the same catalyst loading (5 mg cm^−2^).

**S1.10 Cell Overpotential Breakdown**

The ohmic resistance of the cell was measured using the electrochemical impedance spectroscopy (EIS) to determine cell voltage breakdown and overpotential subdivision. The ohmic overpotential (η_ohm_) of cell was then calculated using the formula:

$\eta_{ohm}=iR$ (S3)

We obtained a semi-logarithmic Tafel plot from the 100% *iR*-corrected polarization plot and determined the kinetic overpotential (η_kin_) from the extrapolated Tafel line. Note that *iR*-correction was not applied for the AEMWE tests, except for the Tafel plot. The mass-transfer overpotential (η_mass_) was measured from the remaining overpotential using following equation:

$\eta_{mass}=E -E_{0}- \eta_{kin} - \eta_{ohm}$ (S4)

where E represents the cell potential at a specific current density, and E_0_ is the theoretical potential for water electrolysis at 80 ± 3 °C.

**S1.11 Cell Efficiency Measurements**

The hydrogen volume generated by the AEMWE cell was supervised in real time, and the cell efficiency (ε_cell_) was determined using the following equation:

$\varepsilon_{cell}=\frac{E_{output}}{E_{input}}=\frac{V_{H_{2}}\times H_{C}}{E_{input}}\times100$ (S5)

Here, V_H2_ is the volume of hydrogen gas (m^3^) generated at 0.50 A cm⁻^2^ for 60 s, H_C_ denotes the calorific value of hydrogen (10.8 × 10^6^ J m⁻^3^), and E_input_ refers to the electrical energy input, calculated at current densities of 0.50 A cm⁻^3^ over a period of 60 s. The gas volume was determined as the average of three independent measurements. The E_input_ was calculated using the following equation:

$E_{input}=Voltage\times time\times current density\times surface area$ (S6)

**S1.12 Energy Consumption Efficiency Measurements**

The energy consumption (E_cons_) per standard cubic meter (Nm³) of hydrogen was calculated using the following formula:

$E_{cons}=\frac{E_{input}(J)}{V_{H2}(m^{3})}$ (S7)

To express E_cons_ in kilowatt-hours per normal cubic meter (kWh Nm⁻^3^), the calculated value of E_cons_ (in J m⁻^3^) was divided by 3.6 × 10^6^ J, which is equivalent to 1 kWh.

The energy efficiency of the system (ε_cons_) based on power consumption was determined using the formula:

$\varepsilon_{cons}=\frac{Theoretical energy required}{E_{cons}}\times100$ (S8)

The theoretical energy required for water electrolysis is considered to be 3.54 kWh Nm⁻^3^ of H_2_ under ideal conditions. E_cons_ is the value measured experimentally for the system.

**S2 Supplementary Results**

**S2.1 OER Performance of the NiFe/ATNT with Different Amounts of NiFe**

The OER performance of the NiFe/ATNT with different amounts of NiFe loading was measured (Fig. S11). The OER activity improved as the loading of NiFe NPs increased from 1 to 5 mg cm^−2^ and then decreased at 10 mg cm^−2^. FESEM images showed that the number and size of the NiFe NPs increased from 1 to 5 mg cm^−2^ of the loading amounts, with limited agglomeration on the ATNT surface (Fig. S12a-c). However, further increasing the NiFe NPs loading to 10 mg cm^−2^, particles exhibited aggregation or clustering on the ATNT (Fig. S12d), which in turn reduced the effective utilization of the active surface area of the electrode and thus reduced the OER performances [S1]. Moreover, higher catalyst loadings can densely pack OER active sites, limiting mass transport and impeding electrolyte penetration, ultimately reducing reaction rates and overall performance [S2].

**S2.2 Photoelectrochemical (PEC) Characterization**

The semiconducting properties of the TiO_2_ with high resistance are the general obstacle to utilize for electrode material. To determine the metallic or semiconductor properties of the TF, TNT, and ATNT, PEC characterization was carried out in 0.1 M KOH solution under simulated solar irradiation (100 mW cm^−2^). The PEC OER activity of the TF was found to be negligible (Fig. S15). The ATNT electrode containing TiO_2_ layers exhibited a low photocurrent of 0.03 mA cm^−2^ at 1.20 V_RHE_, suggesting the poor semiconducting property of ATNT. After electrodeposition of NiFe on the Ti-based substrates, all electrodes showed metallic properties with no PEC OER activity despite the presence of surface TiO_2_ layers. In addition, EIS analysis also revealed the metallic properties of the Ti-based substrate before and after electrodeposition of NiFe, indicating no loss of metallic property due to the creation of a very thin TiO_2_ layer on the surface (Fig. S14).

**S2.3 AEMWE Performance of the NiFe/ATNT-Based Setup with Various Flow Rates**

The optimal electrolyte flow rate efficiently separates the gas from the electrolyte to improve gas purity, avoids bubble accumulation on the electrode to expose more active sites, and aids in maintaining a constant temperature in the electrolyzer when operating at higher temperatures [S3, S4]. Therefore, we experimented with the NiFe/ATNT ǁ Pt/C/ATNT by controlling the electrolyte flow rate of 1, 2, 3, and 5 mL min^−1^ at the anode, and a constant flow rate of 3 mL min^−1^ at the cathode. As shown in Fig. S24b, increasing the flow rate from 1 to 3 mL min^−1^ resulted in an improvement in electrolyzer performance from 0.80 to 1.28 A cm^−2^ at 1.80 V and 60 ± 3 °C, attributed to improved reactant availability at the electrodes [S4]. Previous literature has shown that rapid removal of gas bubbles formed at the anode (O_2_) and cathode (H_2_) by increasing the electrolyte flow rate is vital to prevent the formation of dead zones at the electrodes [S4]. However, the flow rate of 5 mL min^−1^ led to a decline in the performance to 1.07 A cm^−2^. One of the possible reasons is the rapid depletion of OH^−^ ions from the electrode surface at higher flow rates, limiting the interaction of the catalyst with these ions. Considering the results, we determined a flow rate of 3 mL min^−1^ as the optimal condition for effective electrolyte circulation [S4]. Considering the results, we determined a flow rate of 3 mL min^−1^ as the optimal condition for effective electrolyte circulation.

**S2.4 AEMWE Performance of the NiFe/ATNT-Based Setup with Different Operating Temperatures**

In general, higher temperatures improve the electrolyzer performance, which is attributed to several factors, such as the enhanced ionic conductivity of AEM, improved electrode kinetics, and better mass transport [S4, S5]. In this work, we additionally consider the role of the hydrophilicity of the electrode at higher temperatures in enhancing the efficiency of the electrolyzer in terms of facilitating the separation of gas bubbles and ensuring the immediate availability of catalytic sites for subsequent reactions [S4]. This behavior contrasts with hydrophobic electrodes. The electrolyzer performance was improved by increasing the operating temperature from 25 to 80 ± 3 °C (Fig. S24c). Experiments above 80 ± 3 °C were not performed as higher temperatures can rapidly decompose the AEM. By optimizing the all-possible parameters, the NiFe/ATNT ǁ Pt/C/ATNT electrolyzer designed with PiperION membrane showed a current density of 1.67 A cm^−2^ at 1.80 V and 80 ± 3 °C under a flow rate of 3 mL min^−1^.

**Supplementary Figures and Tables**


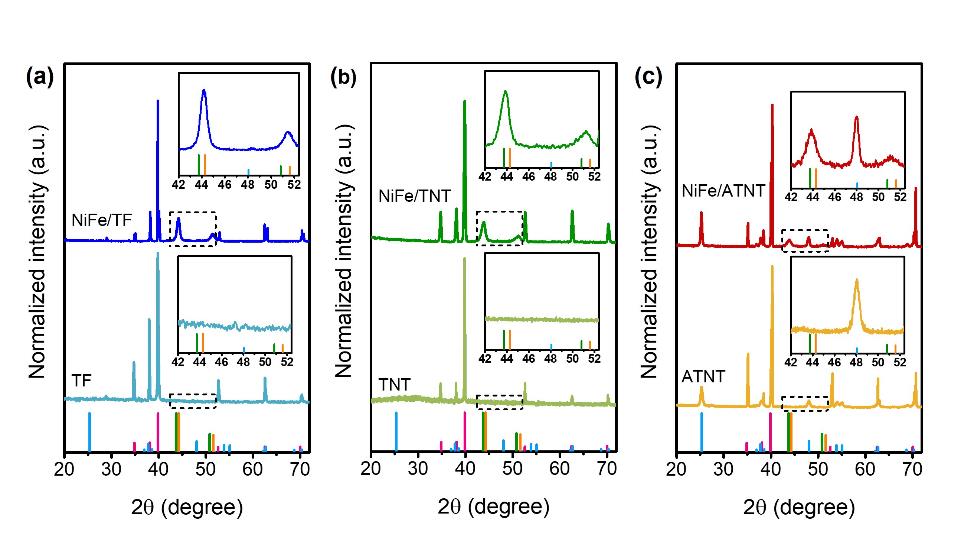


**Fig. S1** SXRD analysis. The SXRD patterns before and after NiFe electrodeposition on **a** TF, **b** TNT, and **c** ATNT substrates. References: pink bars: Ti (JCPDS# 00-044-1294), blue bars: TiO_2_ (JCPDS# 00-021-1272), orange bars: Ni (JCPDS# 01-071-4653), and green bars: Fe (JCPDS# 01-071-4407)


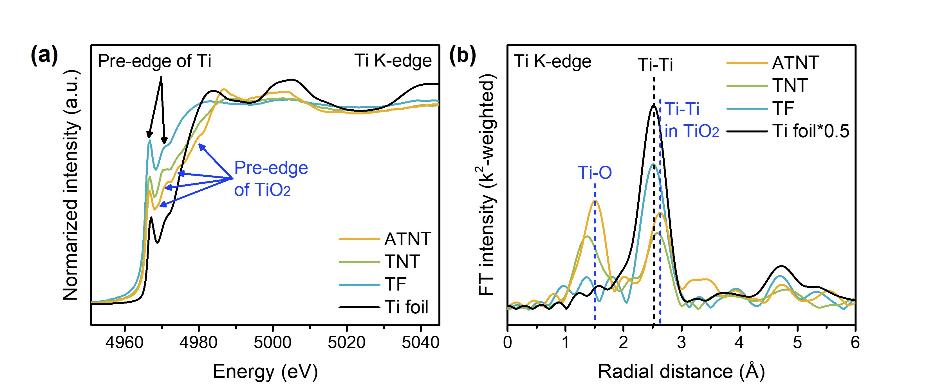


**Fig. S2** XAFS analysis for Ti-based substrates. **a** XANES and **b** FT-EXAFS spectra on Ti K-edge for Ti-based substrates


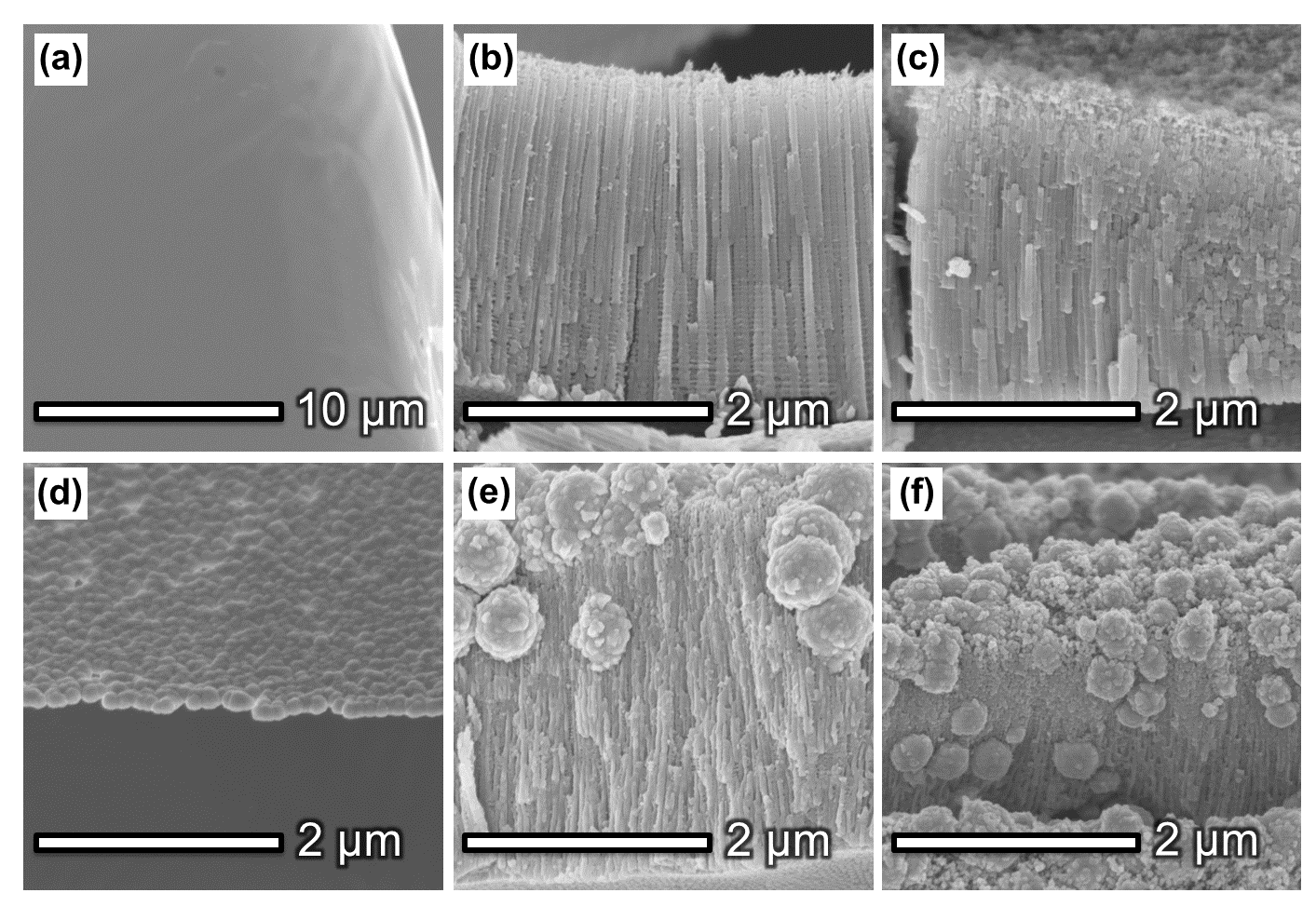


**Fig. S3** Cross-sectional FESEM images. FESEM images with cross-sectional viewing before and after electrodeposition of NiFe on **a**, **d** TF, **b**, **e** TNT, and **c**, **f** ATNT substrates


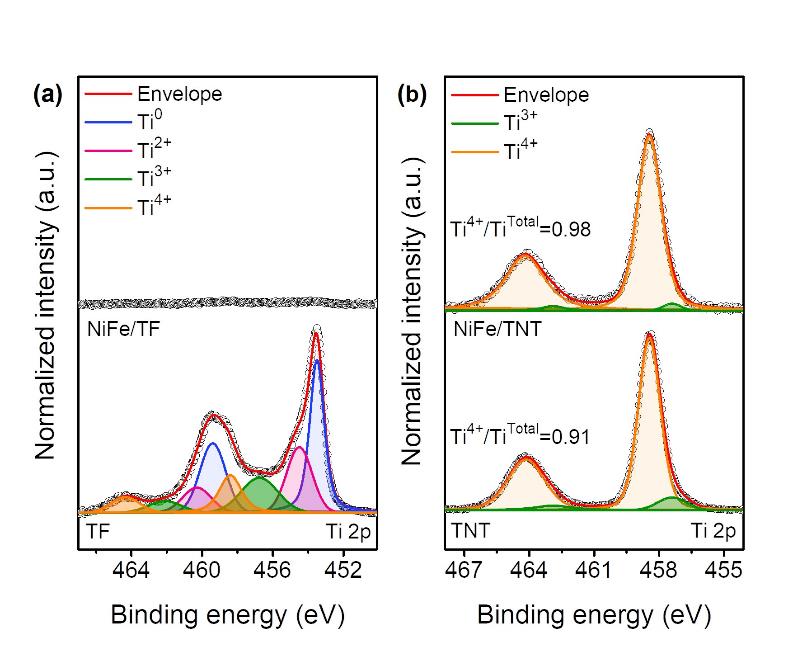


**Fig. S4** XPS results on Ti 2p region. The XPS spectra before and after NiFe electrodeposition on **a** TF and **b** TNT substrates


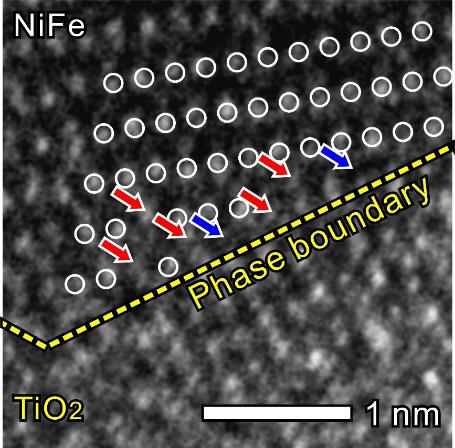


**Fig. S5** Atomic-resolution HRTEM image of the NiFe/ATNT representing atomic defects at the interfacial site between a NiFe nanoparticle and TiO_2_ substrate. (Red and blue arrows indicating atomic dislocation and Ni/Fe vacancy of NiFe nanoparticle, respectively)


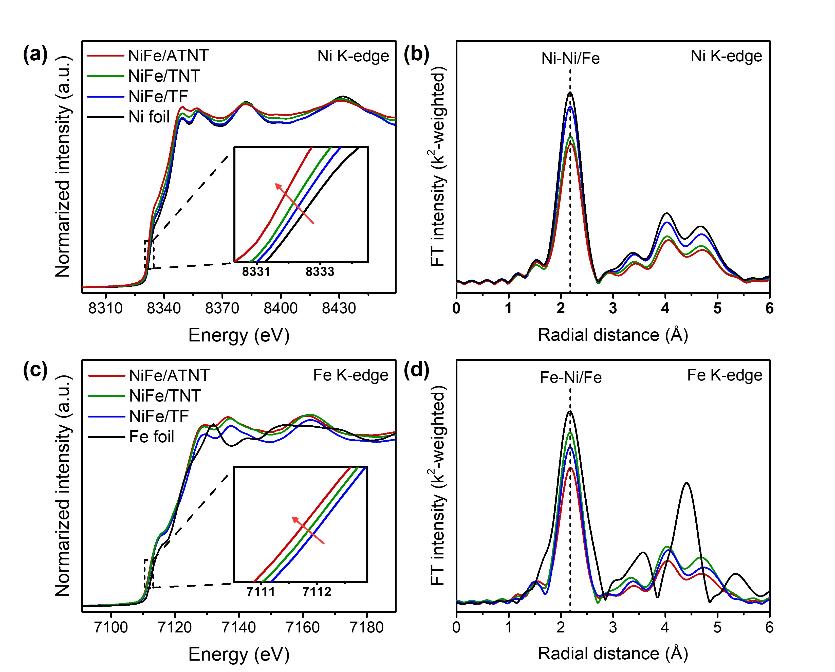


**Fig. S6** XAFS analysis for electrodeposited NiFe on Ti-based substrates. The XANES and FT-EXAFS profiles on **a**, **b** Ni K-edge and **c**, **d** Fe K-edge


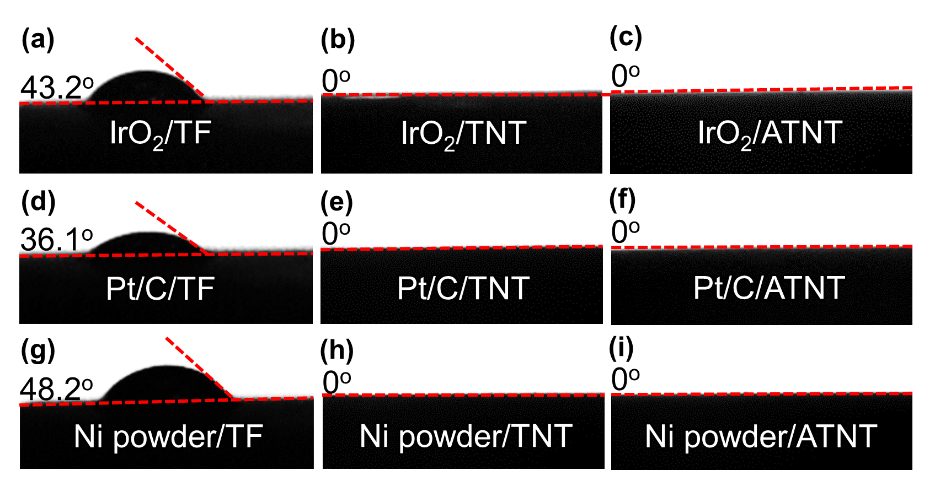


**Fig. S7** The contact angles of a water droplet on different Ti-based substrates (TF, TNT, and ATNT) for various commercial catalysts: **a-c** IrO_2_, **d-f** Pt/C, and **g-i** Ni powder


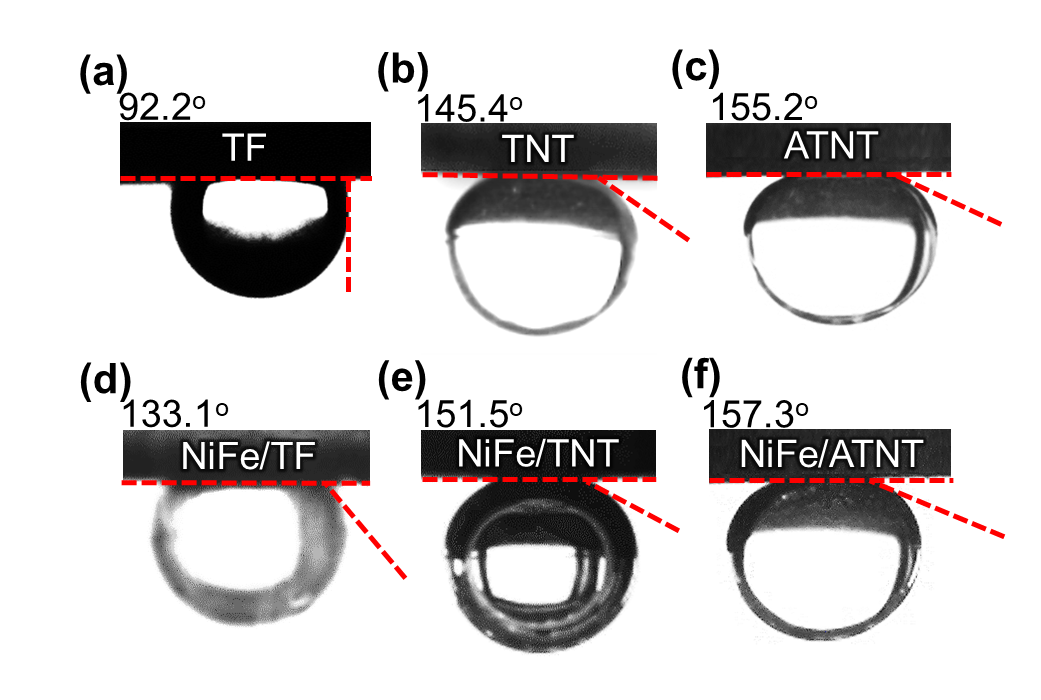


**Fig. S8** The contact angles of a gas bubble on various Ti-based substrates (TF, TNT, and ATNT) before and after NiFe electrodeposition: **a**, **d** TF, **b**, **e** TNT, and **c**, **f** ATNT


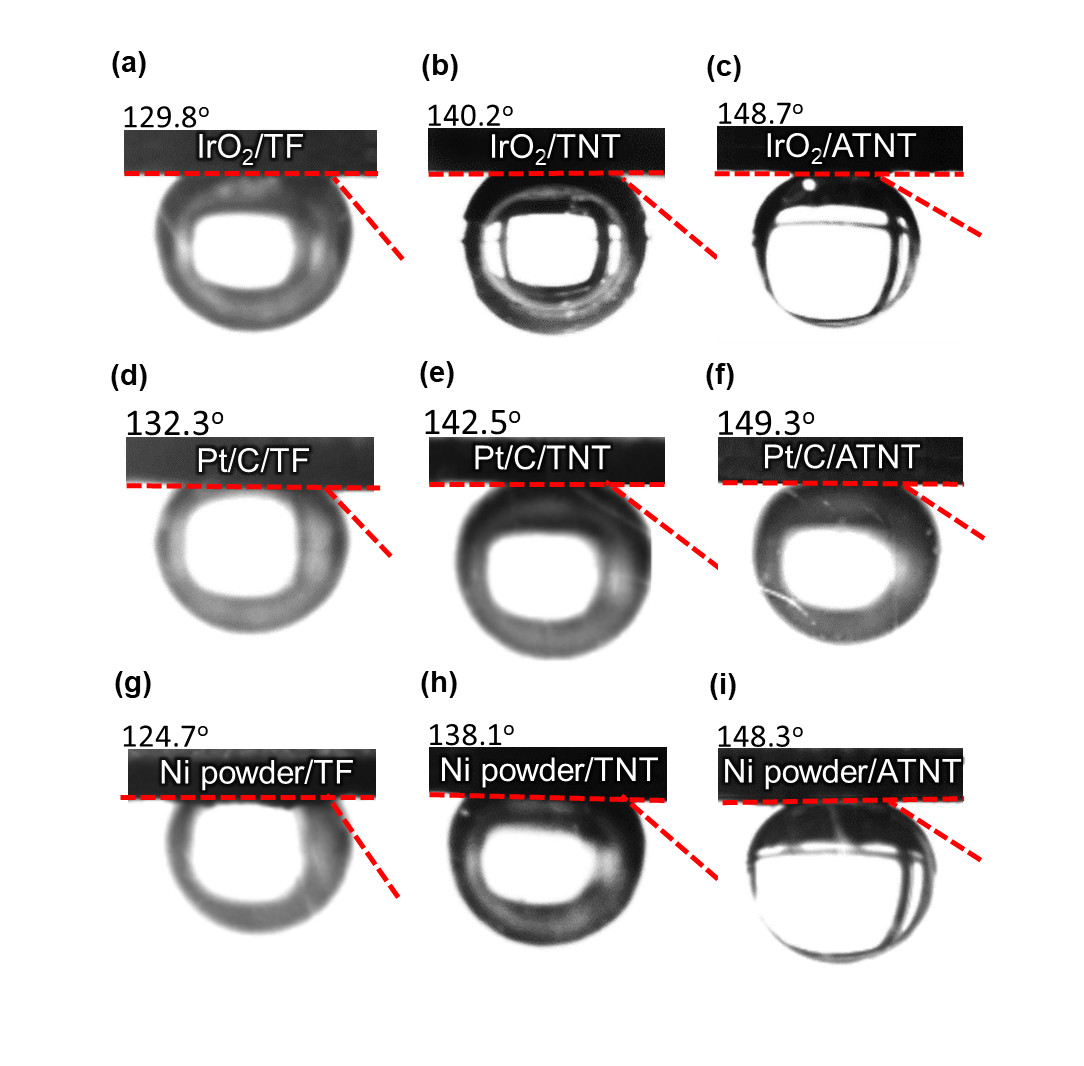


**Fig. S9** The contact angles of a gas bubble on different Ti-based substrates (TF, TNT, and ATNT) for various commercial catalysts: **a**-**c** IrO_2_, **d**-**f** Pt/C, and **g**-**i** Ni powder


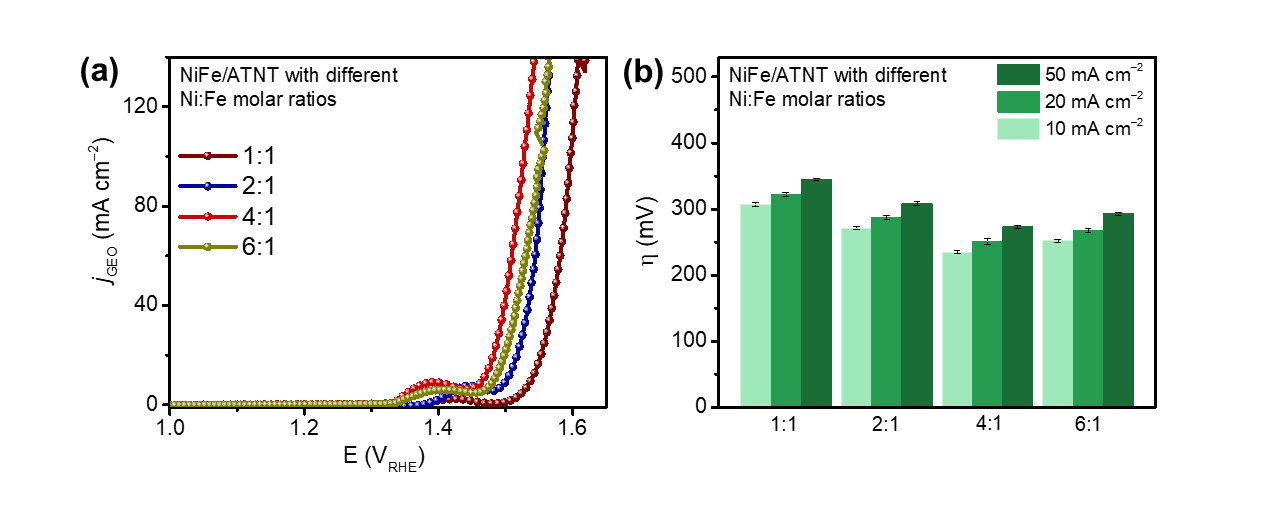


**Fig. S10** OER activities of NiFe/ATNT with different Ni:Fe molar ratios. **a** 100% *iR*-corrected OER polarization curves and **b** comparison of overpotential values at different current densities


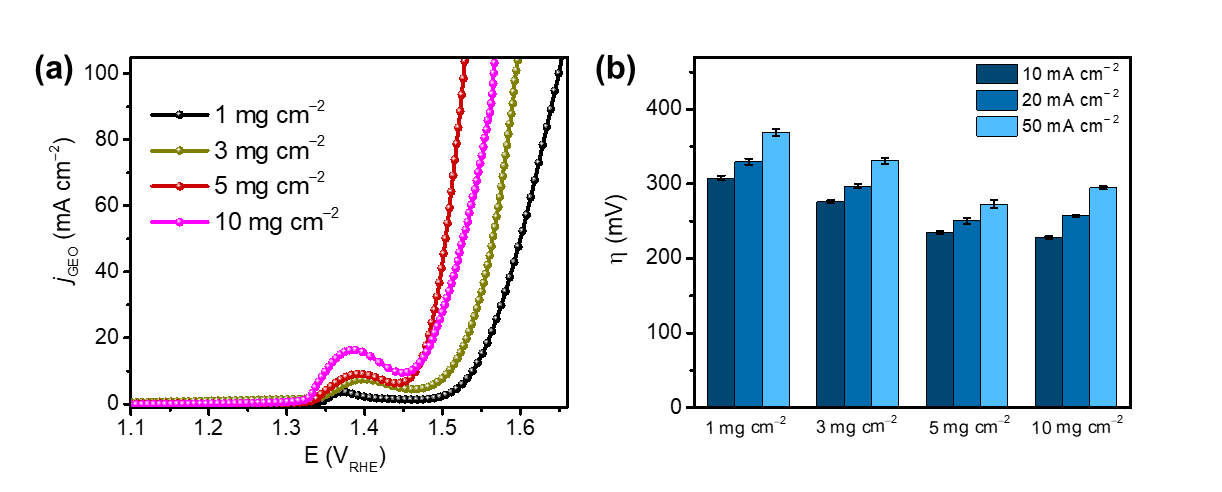


Fig. S11 OER activities of NiFe/ATNT with different NiFe catalyst loading amounts. a 100% *iR*-corrected OER polarization curves and b comparison of overpotential values at different current densities


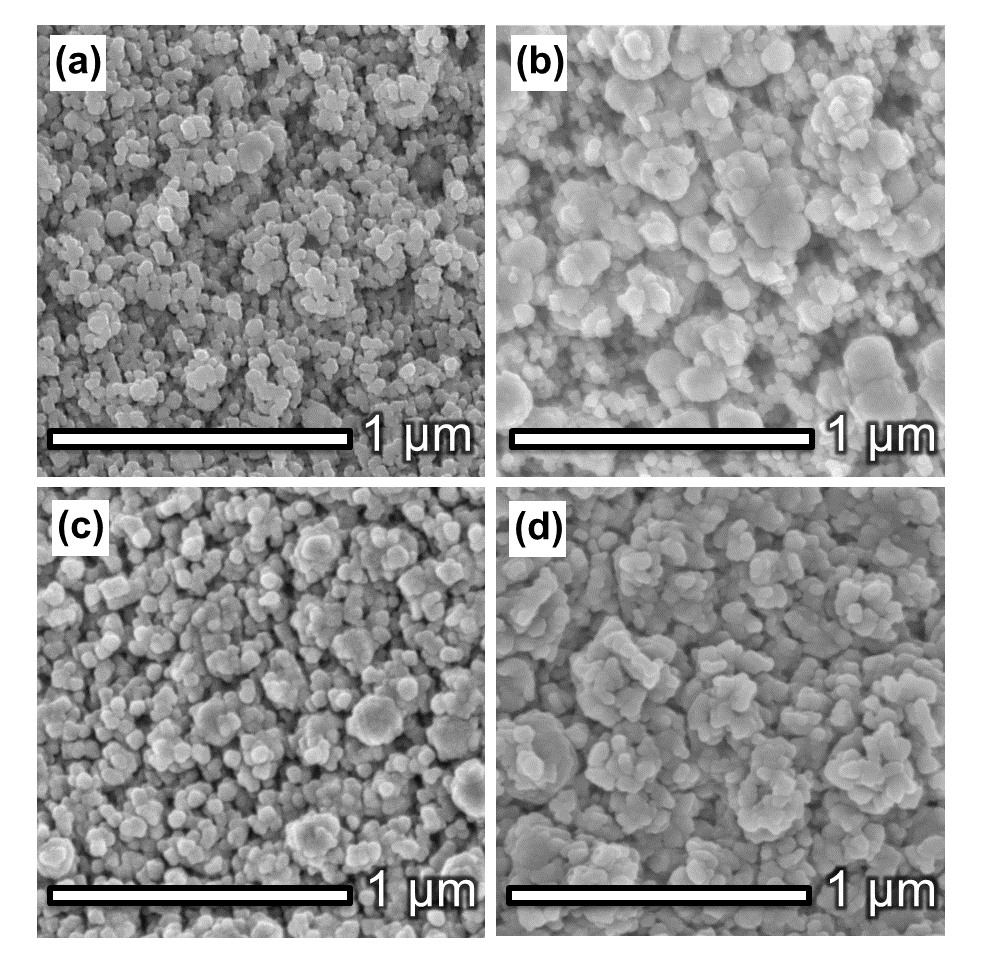


Fig. S12 FESEM images of NiFe/ATNT with various NiFe loading amounts. a 1 mg cm^−2^, b 3 mg cm^−2^, c 5 mg cm^−2^, and d 10 mg cm^−2^

*
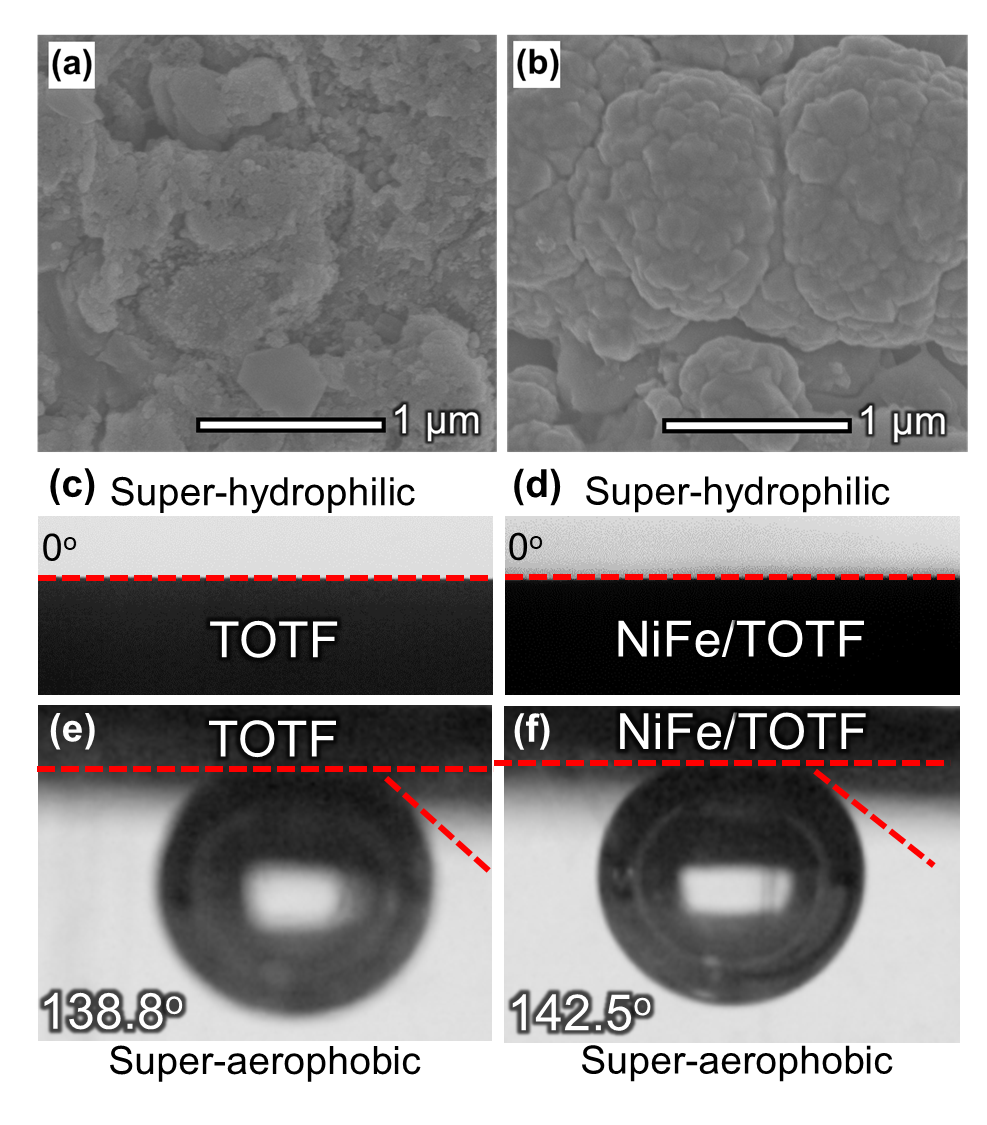
*

**Fig. S13** FESEM images of **a** TOTF and **b** NiFe/TOTF. Contact angles of a water droplet on **c** TOTF and **d** NiFe/TOTF. Contact angles of a gas bubble on **e** TOTF and **f** NiFe/TOTF

**

**

**Fig. S14** EIS analysis of the prepared electrodes


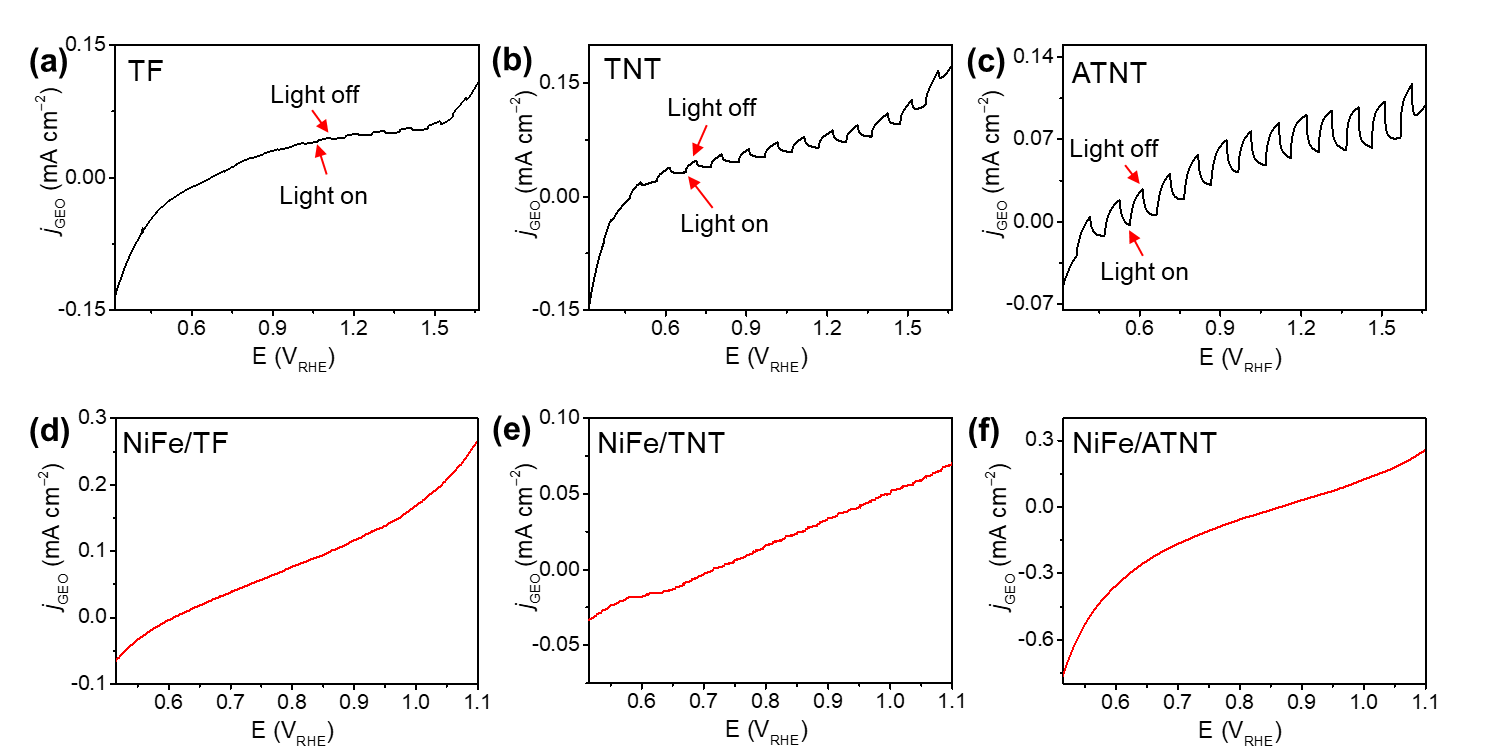


**Fig. S15** PEC characterization. Polarization curves of **a** TF, **b** TNT, **c** ATNT, **d** NiFe/TF, **e** NiFe/TNT, and **f** NiFe/ATNT in a 0.1 M KOH under simulated solar irradiation with a scan rate of 20 mV s^−1^ (light intensity: 100 mW cm^−2^)


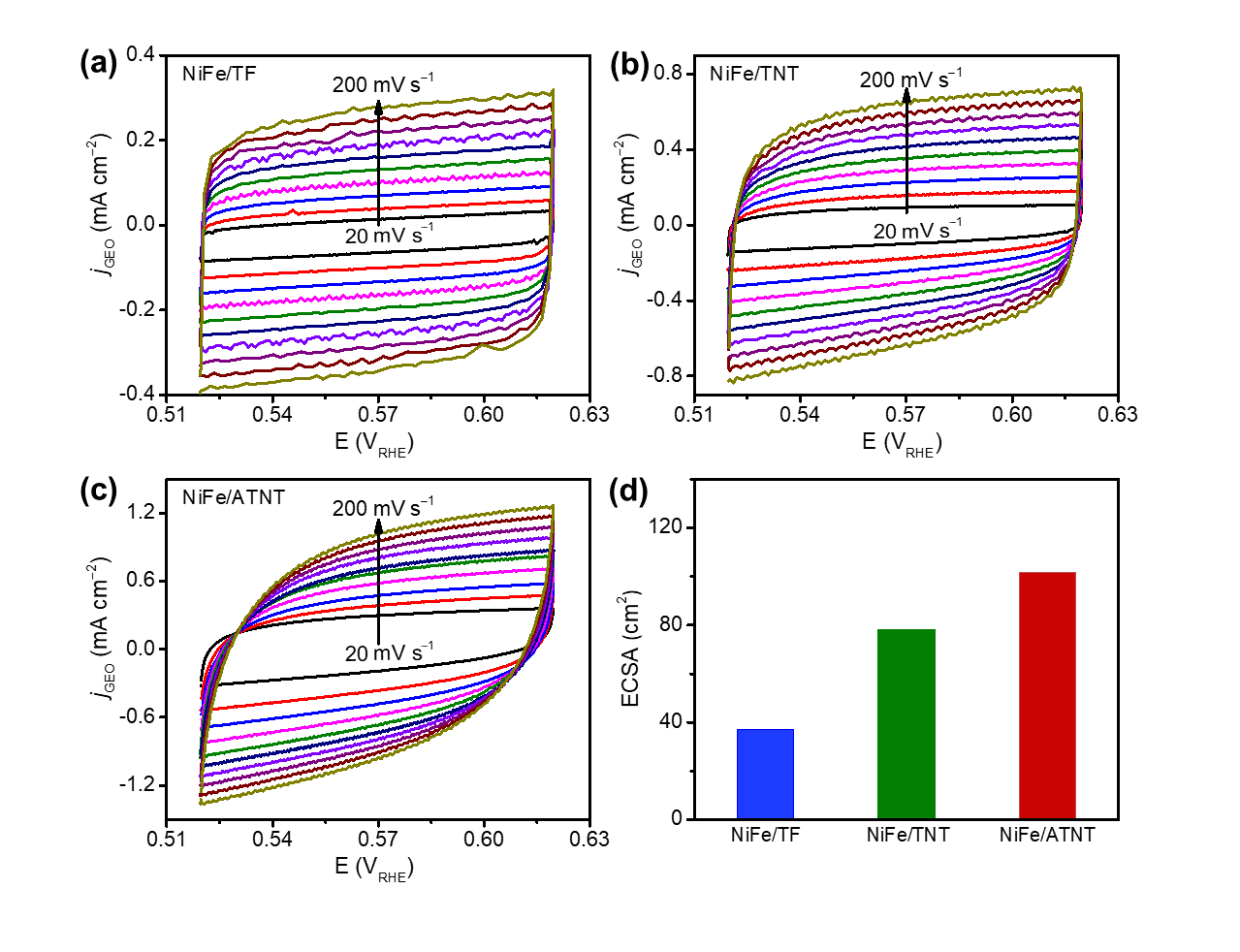


**Fig. S16** ECSA measurements by CV in the non-faradaic region: **a** NiFe/TF, **b** NiFe/TNT, and **c** NiFe/ATN. **d** calculated ECSA results


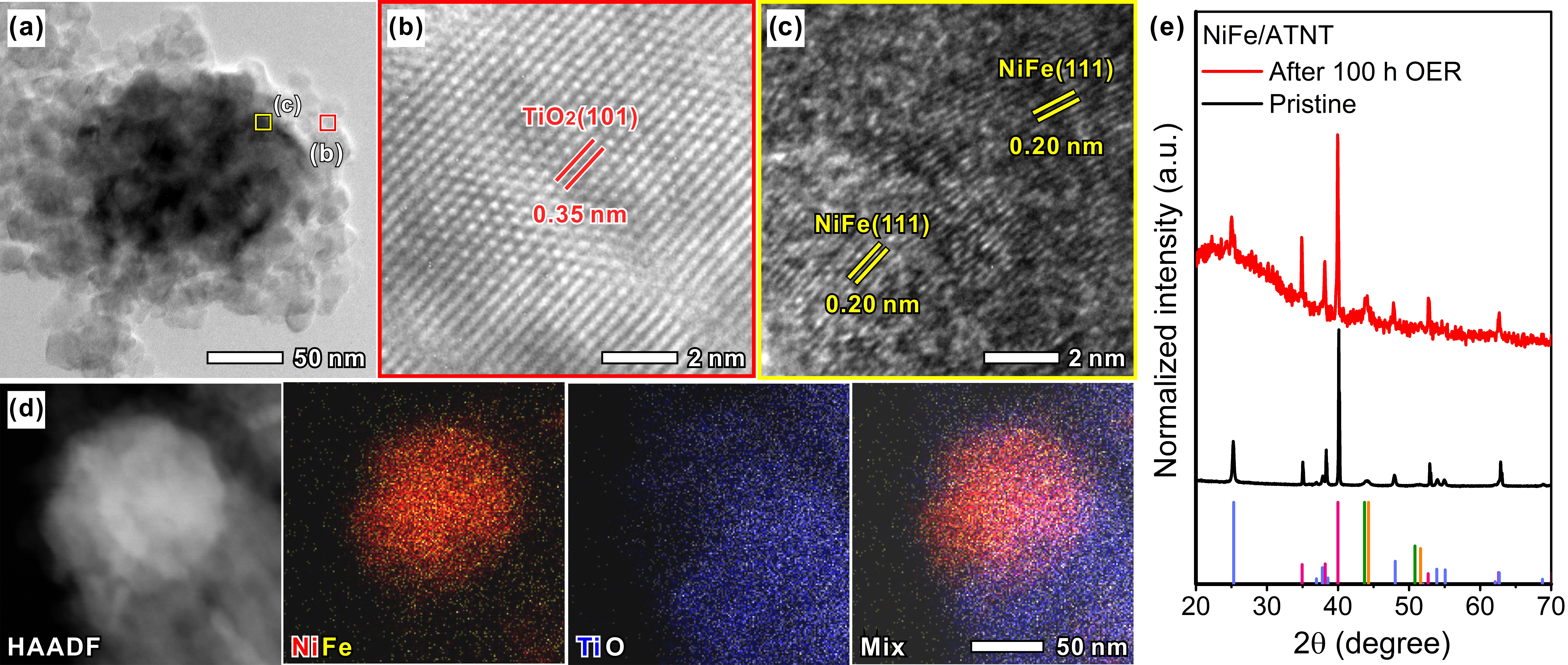


**Fig. S17** Material characterization of the NiFe/ATNT after 100 h OER stability test. **a** TEM image of NiFe on ATNT. **b, c** High-resolution viewing taken from the red and yellow boxes in a. **d** HAADF-STEM image and EDS mapping of a single NiFe particle. **e** XRD profiles of NiFe/ATNT before and after 100 h OER stability test. Pink bars: Ti (JCPDS# 00-044-1294), blue bars: TiO_2_ (JCPDS# 00-021-1272), orange bars: Ni (JCPDS# 01-071-4653), and green bars: Fe (JCPDS# 01-071-4407)


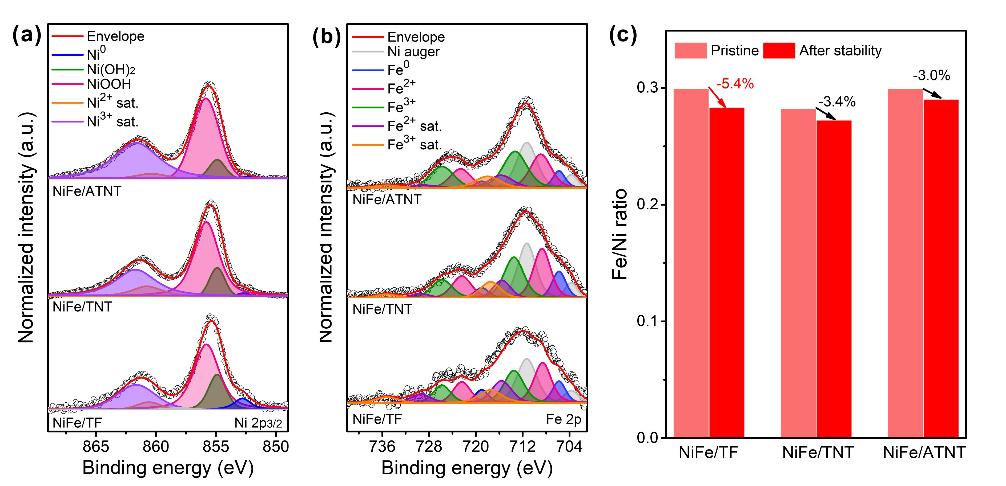


**Fig. S18** XPS results on **a** Ni 2p_3/2_ and **b** Fe 2p regions after 100 h OER stability test. **c** The atomic ratio of Fe to Ni calculated from XPS spectra


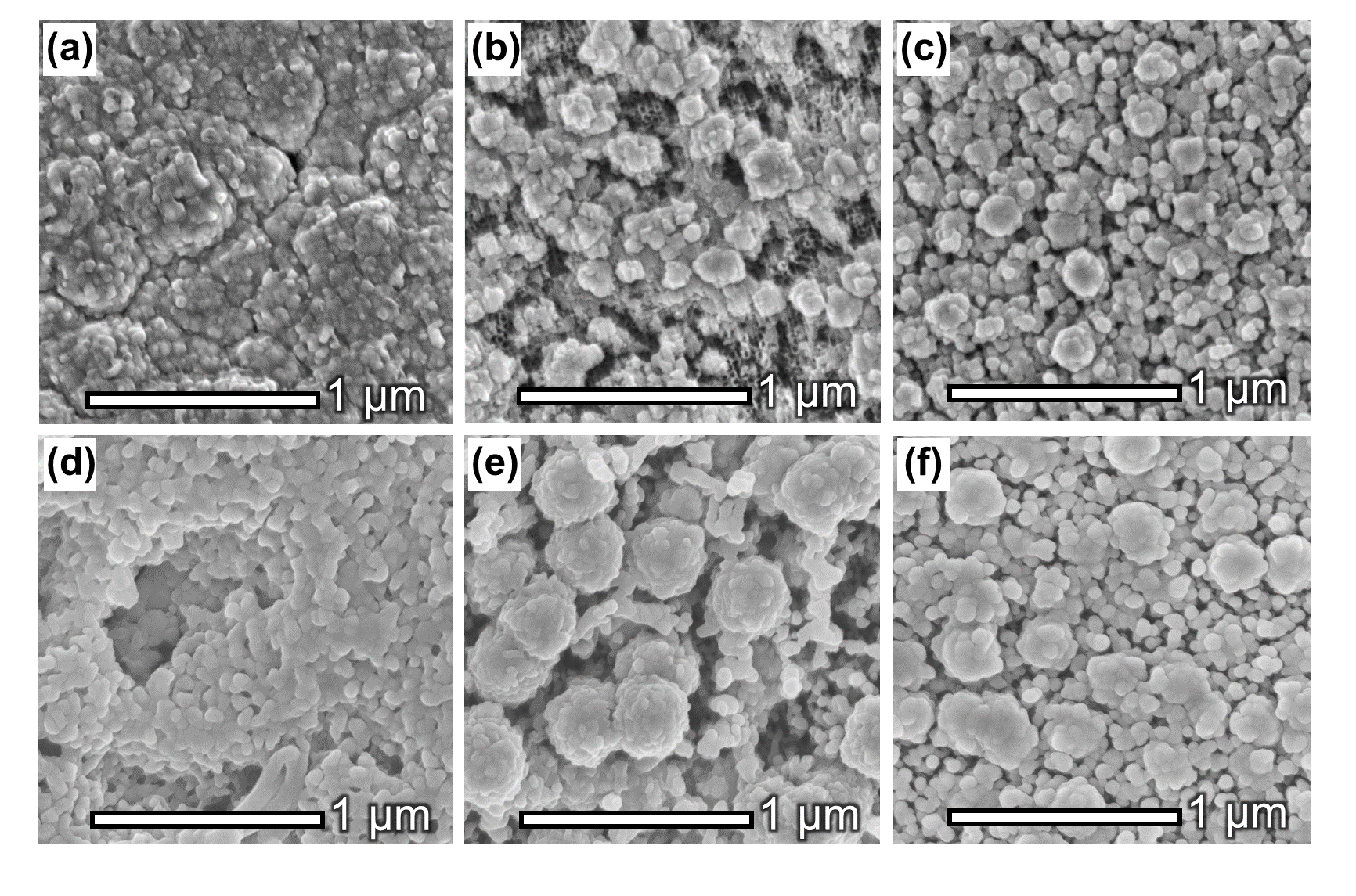


**Fig. S19** FESEM images of prepared electrodes before and after 100 h OER stability test in the half-cell: **a**, **d** NiFe/TF, **b**, **e** NiFe/TNT, and **c**, **f** NiFe/ATNT

**
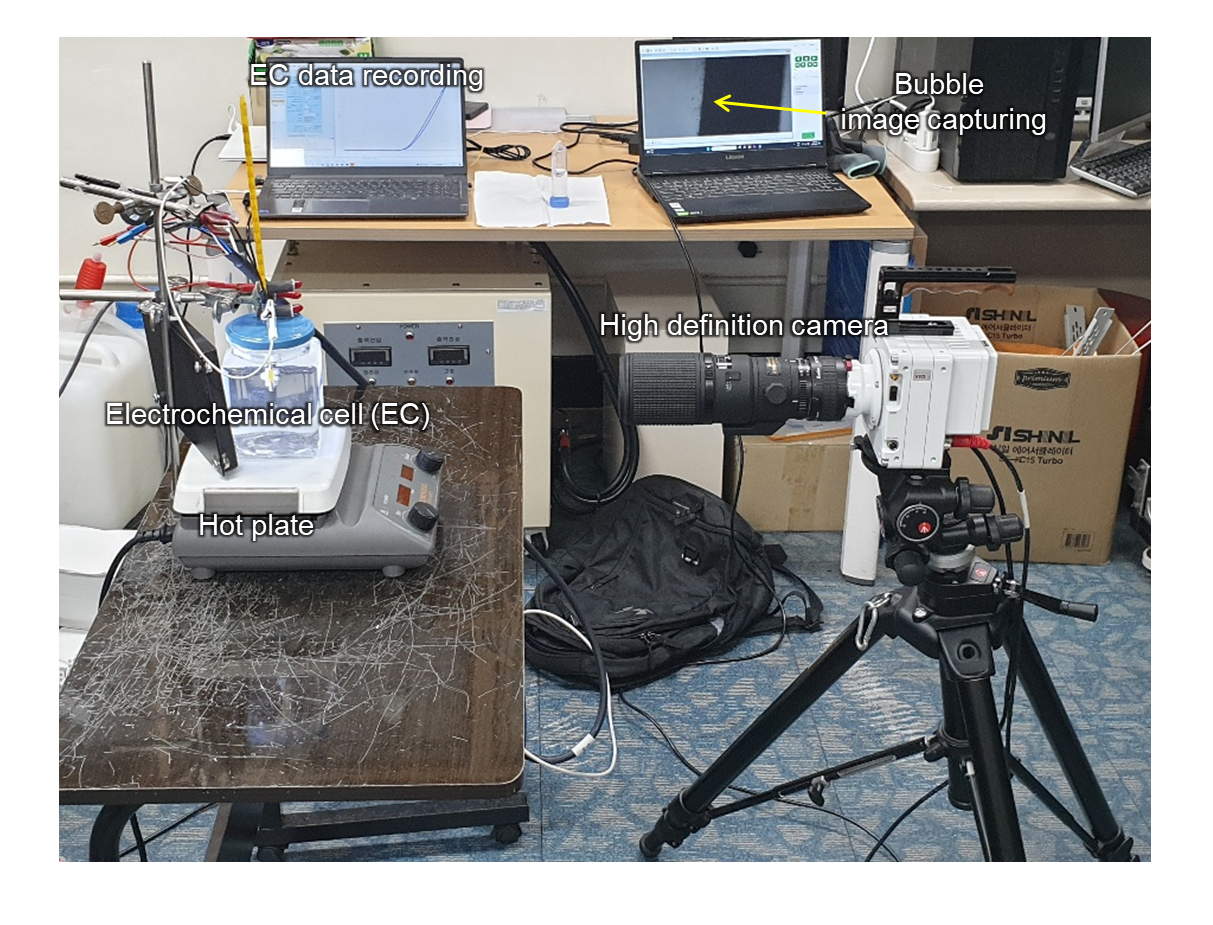
**

Fig. S20 The in-situ bubble capturing setup. In-situ bubble capturing setup employing a high-definition camera during OER measurements


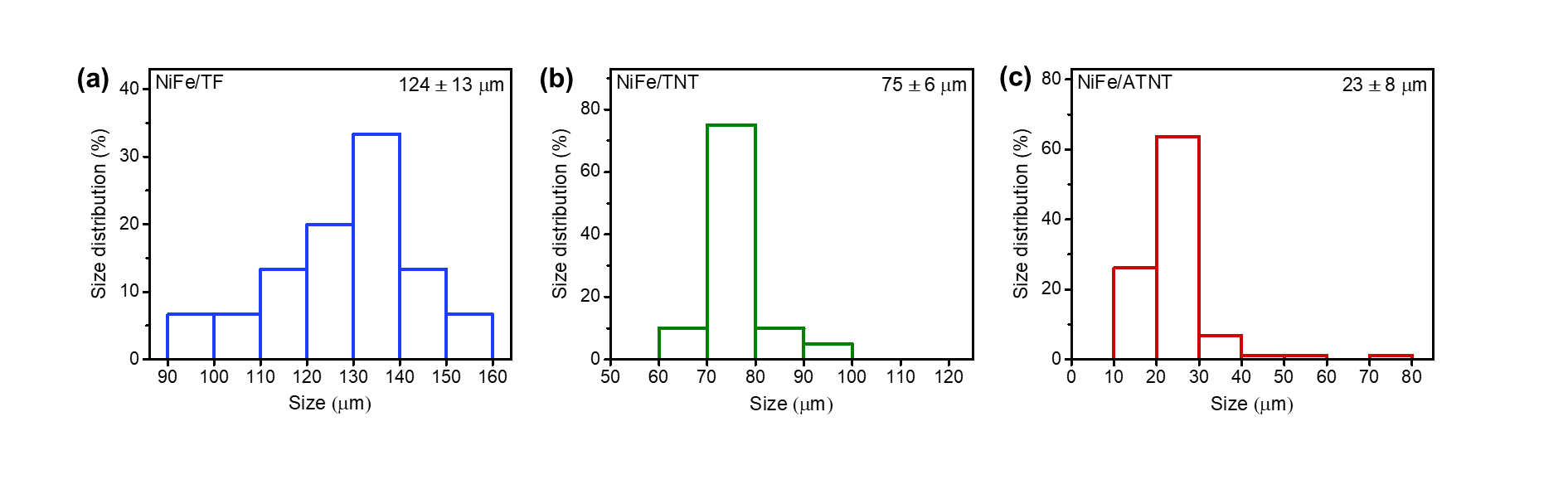


**Fig. S21** Size distribution of O_2_ bubbles. The diameters of O_2_ bubbles released from **a** NiFe/TF, **b** NiFe/TNT, and **c** NiFe/ATNT during OER at 80 ± 3 °C


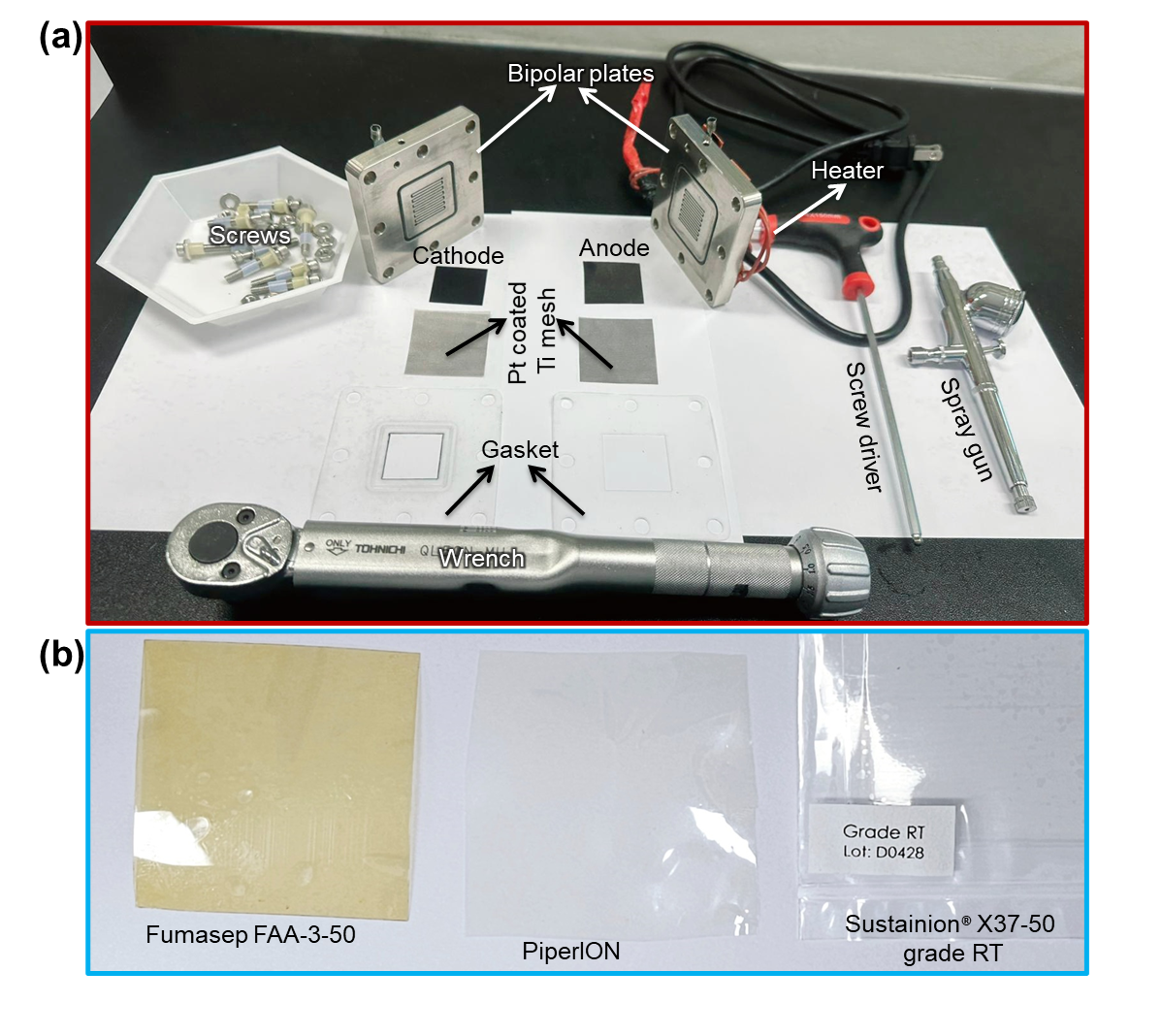


**Fig. S22** Images showcasing AEMWE cell and membranes used in this study. **a** AEMWE cell setup and **b** different types of AEM

**
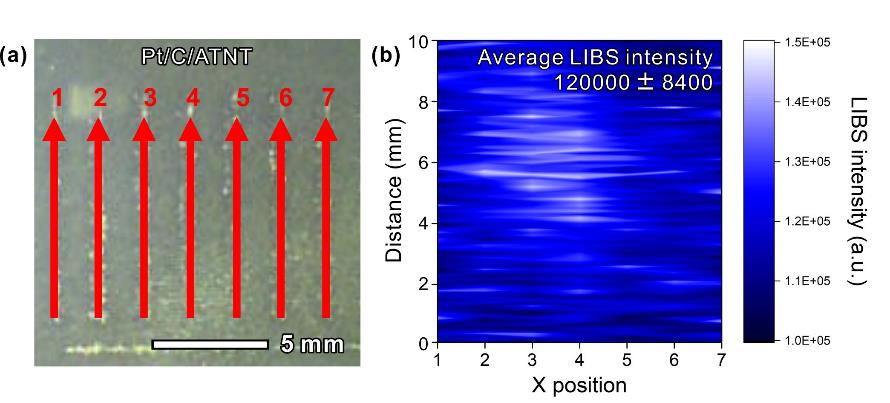
**

**Fig. S23** The spatial uniformity of Pt/C/ATNT electrode. **a** As-prepared Pt/C/ATNT and **b** corresponding 2D LIBS surface map measured from the red arrowed region in **a**


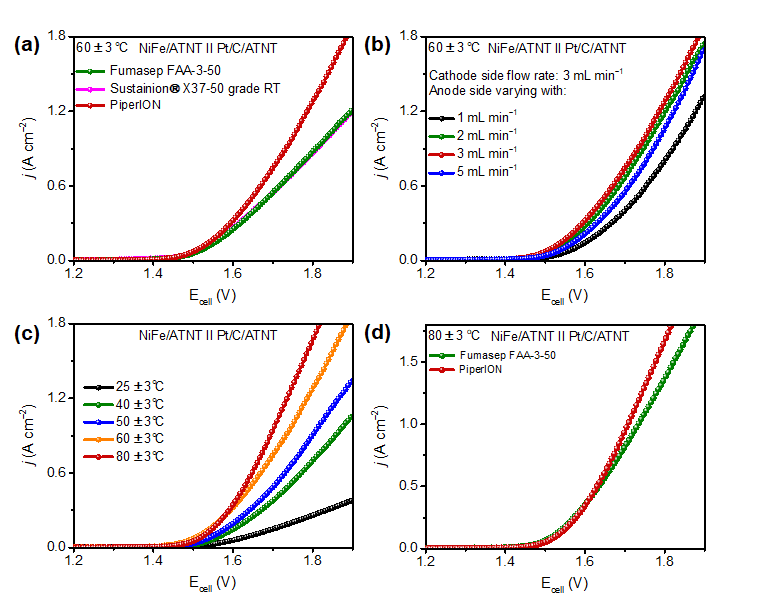


**Fig. S24** Comparison of AEMWE performance. LSV curves obtained **a** with different AEMs at a flow rate of 3 mL min^−1^, **b** at different flow rates of anolyte on anode side, and **c** at different operating temperatures with a flow rate of 3 mL min^−1^. **d** AEMWE performance of the NiFe/ATNT ‖ Pt/C/ATNT configuration using Fumasep FAA-3-50 and PiperION membranes at optimized conditions


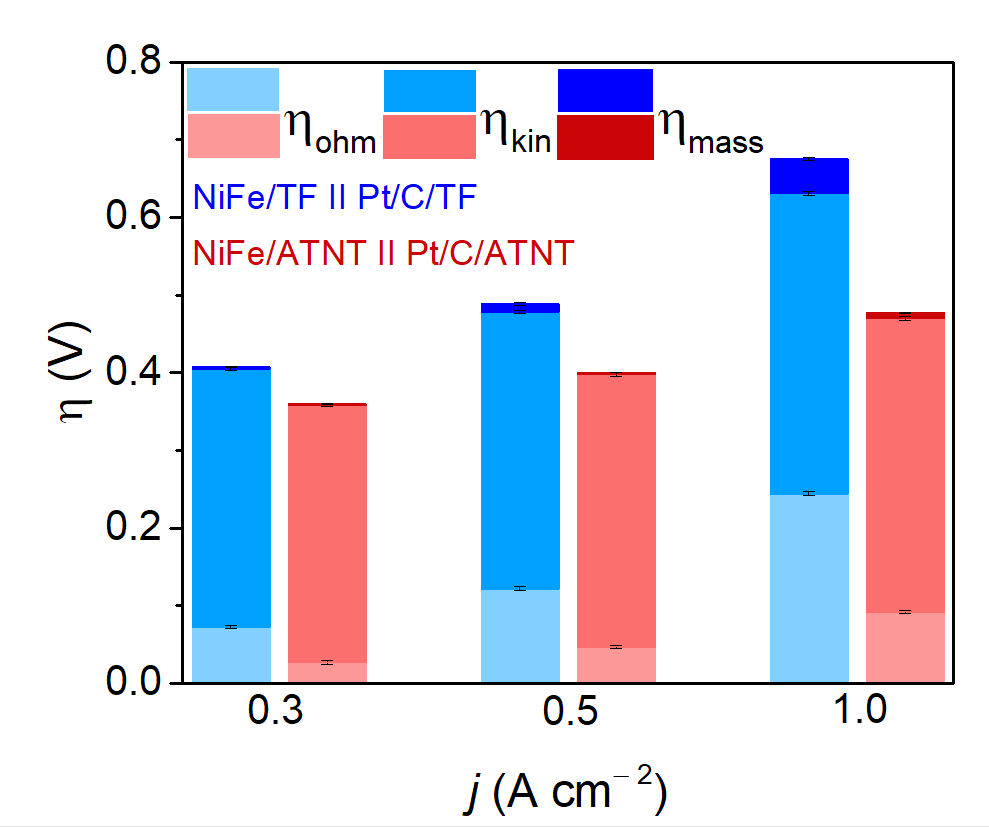


**Fig. S25** Cell overpotential breakdown in AEMWE. The total overpotential of AEMWE is divided into three parts: ohmic overpotential (η_ohm_), kinetic overpotential (η_kin_), and mass-transfer overpotential (η_mass_), at different current densities


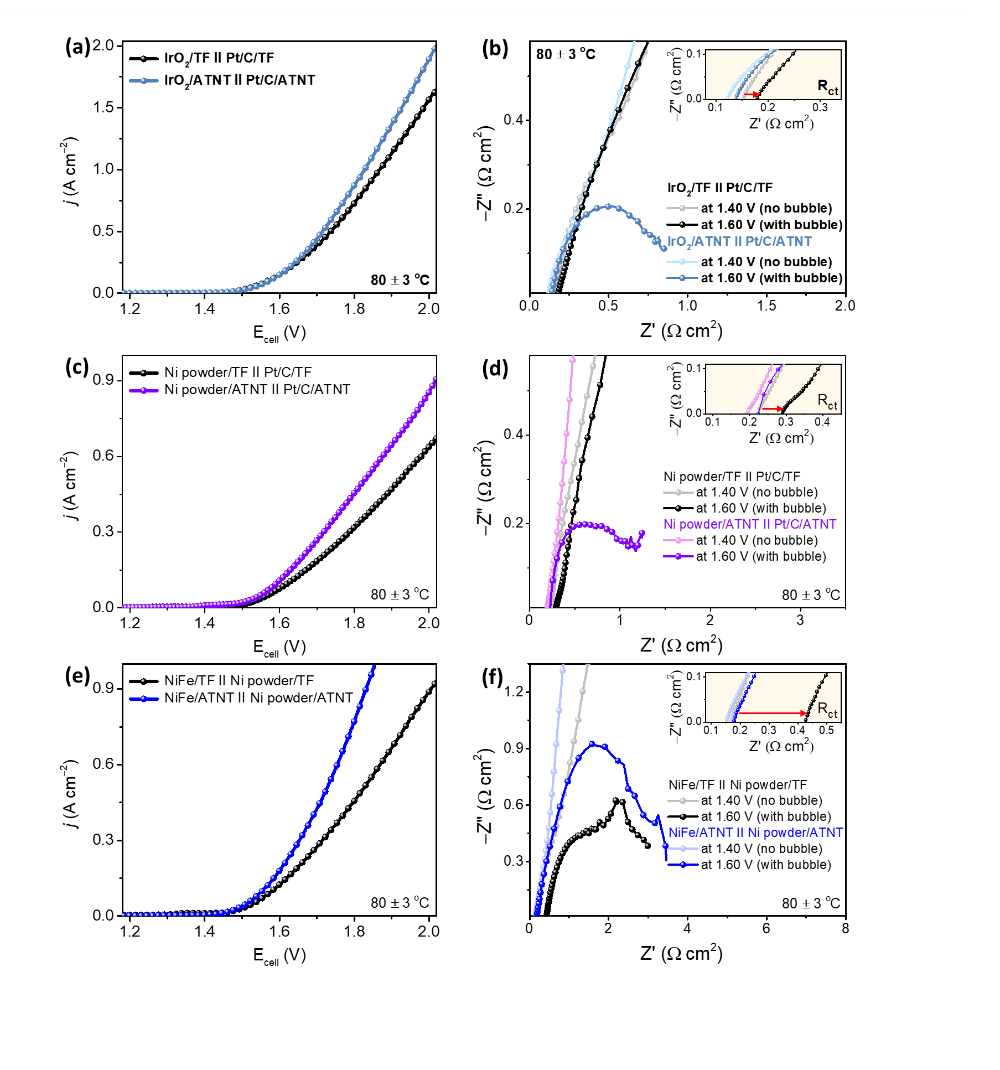


Fig. S26 AEMWE performance of IrO_2_ and Ni powder on different Ti-based substrates with flow rate of 3 mL min^−1^ at a scan rate of 5 mV s^−1^ and at 80 ± 3 °C. a LSV curves for the AEMWE with different setups of IrO_2_/TF ǁ Pt/C/TF and IrO_2_/ATNT ǁ Pt/C/ATNT. b EIS analysis of AEMWE performance with IrO_2_/TF ǁ Pt/C/TF and IrO_2_/ATNT ǁ Pt/C/ATNT setups: a comparative study at 1.40 V (no bubble generation) and 1.60 V (with bubble) at 80 ± 3 °C. c LSV curves for the AEMWE with different setups of Ni powder/TF ǁ Pt/C/TF and Ni powder/ATNT ǁ Pt/C/ATNT. d EIS analysis of AEMWE performance with Ni powder/TF ǁ Pt/C/TF and Ni powder/ATNT ǁ Pt/C/ATNT setups: a comparative study at 1.40 V (no bubble generation) and 1.60 V (with bubble) at 80 ± 3 °C. e LSV curves for the AEMWE with different setups of NiFe/TF ǁ Ni powder/TF and NiFe/ATNT ǁ Ni powder/ATNT. f EIS analysis of AEMWE performance with NiFe/TF ǁ Ni powder/TF and NiFe/ATNT ǁ Ni powder/ATNT setups: a comparative study at 1.40 V (no bubble generation) and 1.60 V (with bubble) at 80 ± 3 °C


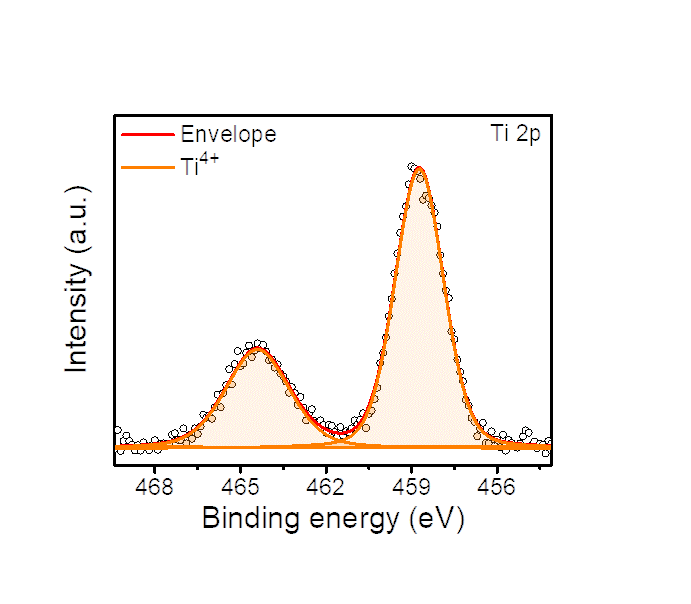


Fig. S27 Material characterization of NiFe/ATNT after 100 h OER stability. The XPS spectrum of NiFe/ATNT on Ti 2p region


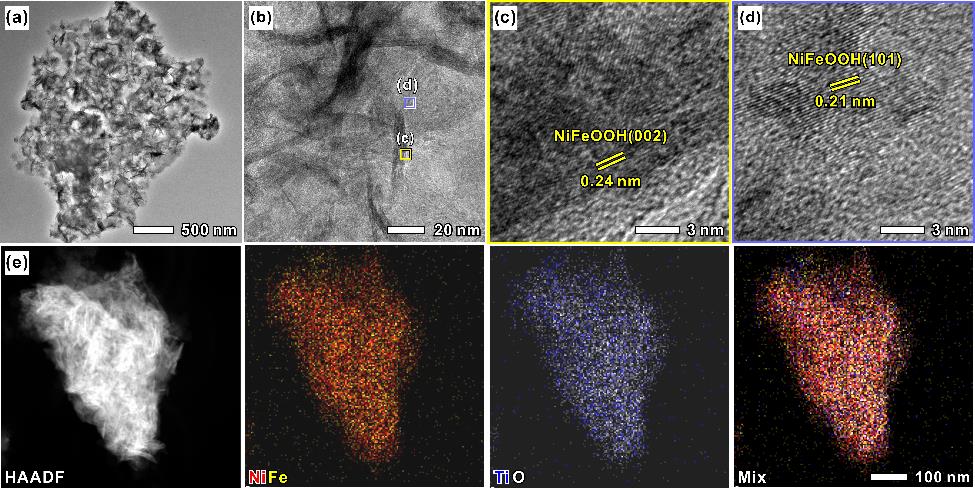


**Fig. S28** Material characterization of the NiFe/ATNT after AEMWE stability test. **a, b** HRTEM images of NiFe/ATNT. **c, d** Magnified viewing taken from the yellow and blue boxes in b. **e** HAADF-STEM image and EDS elemental mapping results


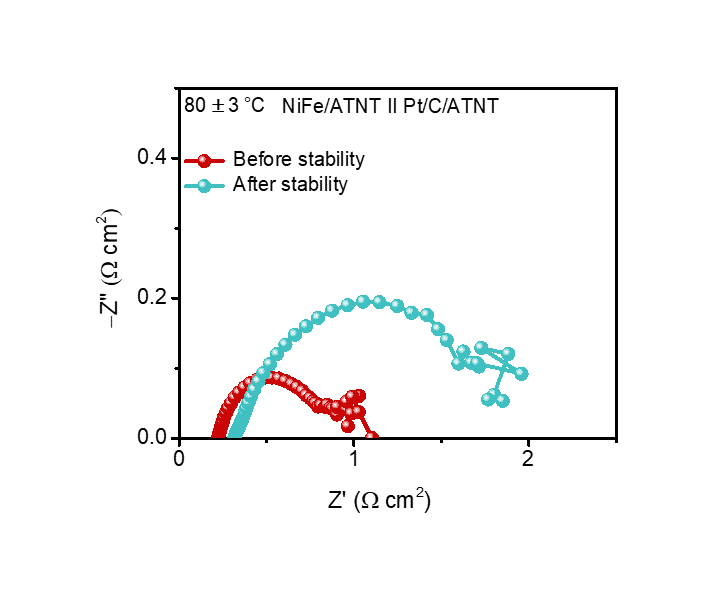


Fig. S29 EIS measurements. EIS of the NiFe/ATNT ǁ Pt/C/ATNT before and after 1500 h stability test at 1.60 V

**
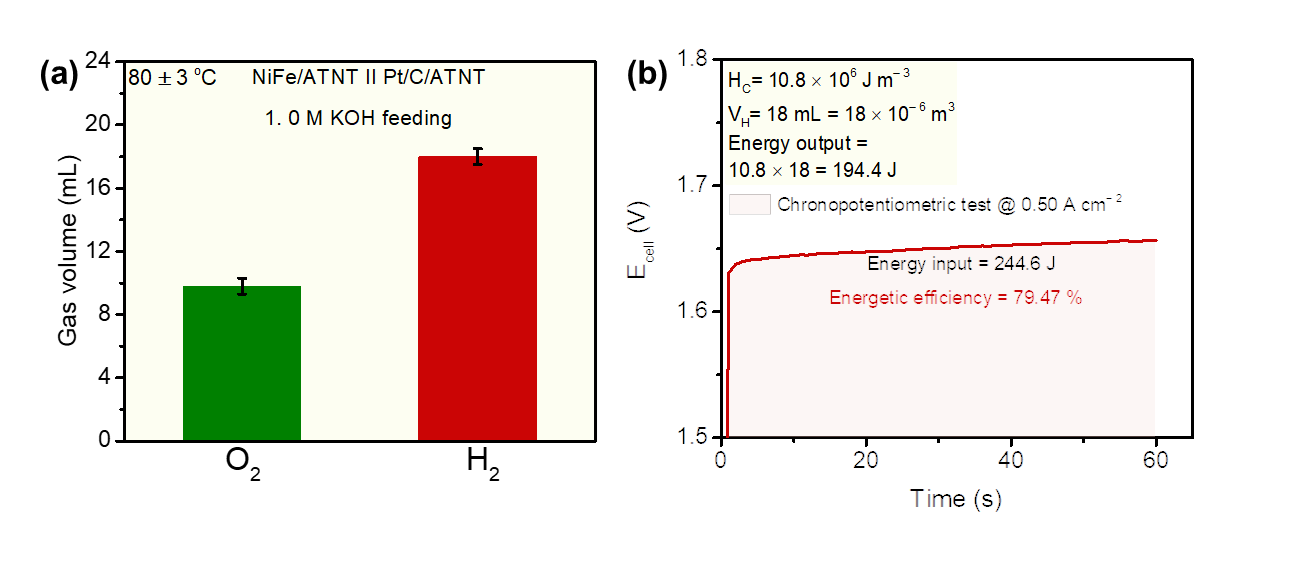
**

**Fig. S30** Cell efficiency of the NiFe/ATNT ‖ Pt/C/ATNT configuration with 1.0 M KOH at 1.80 V and 80 ± 3 °C. **a** The generated O_2_ and H_2_ gas volumes during **b** a chronopotentiometric test at a current density of 0.50 A cm⁻^2^ for 60 s with 1.0 M KOH. Energy output was calculated based on the calorific value of H_2_

**
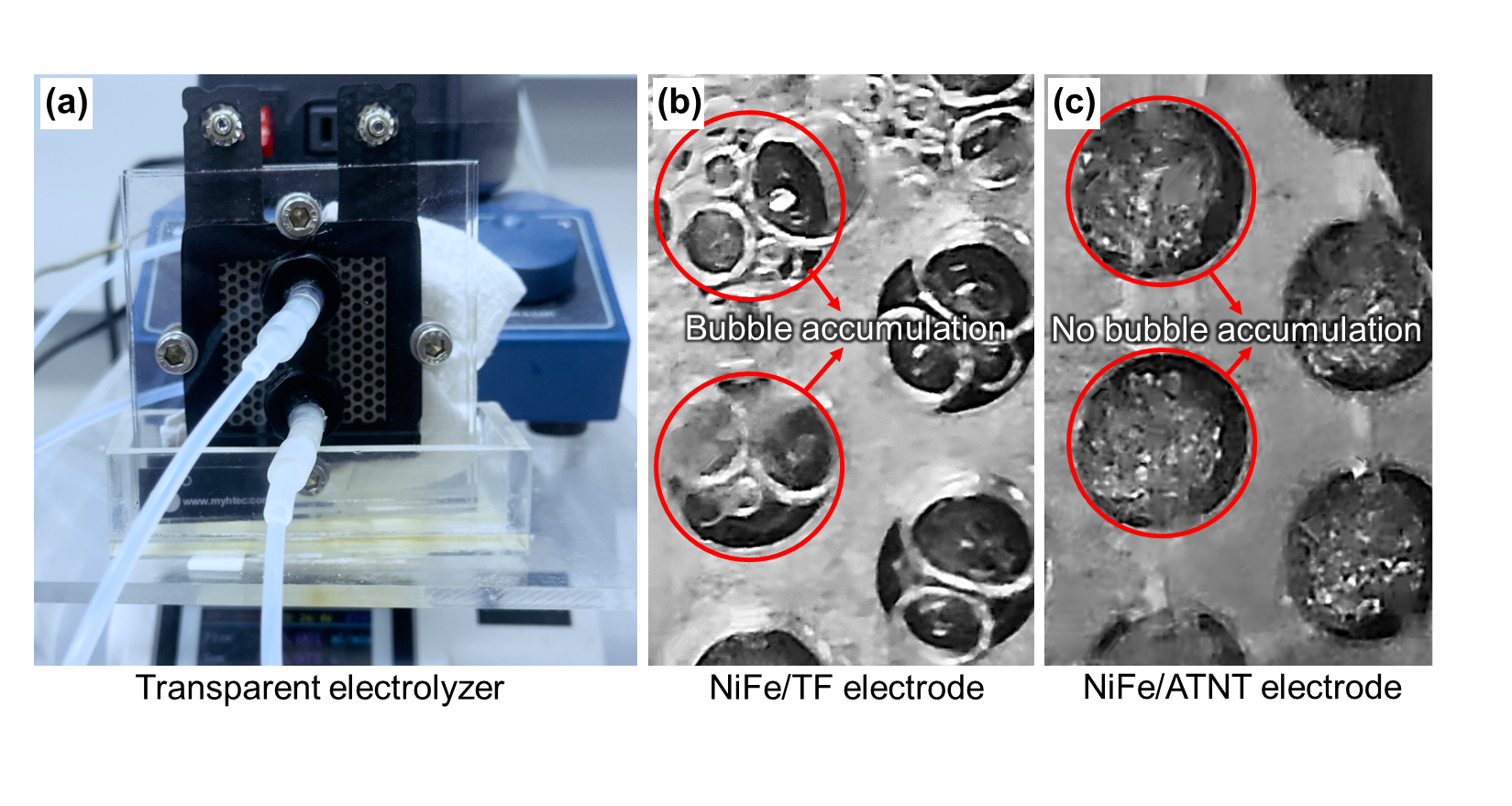
**

**Fig. S31 a** Transparent electrolyzer. Captured in-situ images of gas bubbles release pattern from the transparent electrolyzer with **b** the NiFe/TF electrode setup and **c** the NiFe/ATNT electrode setup


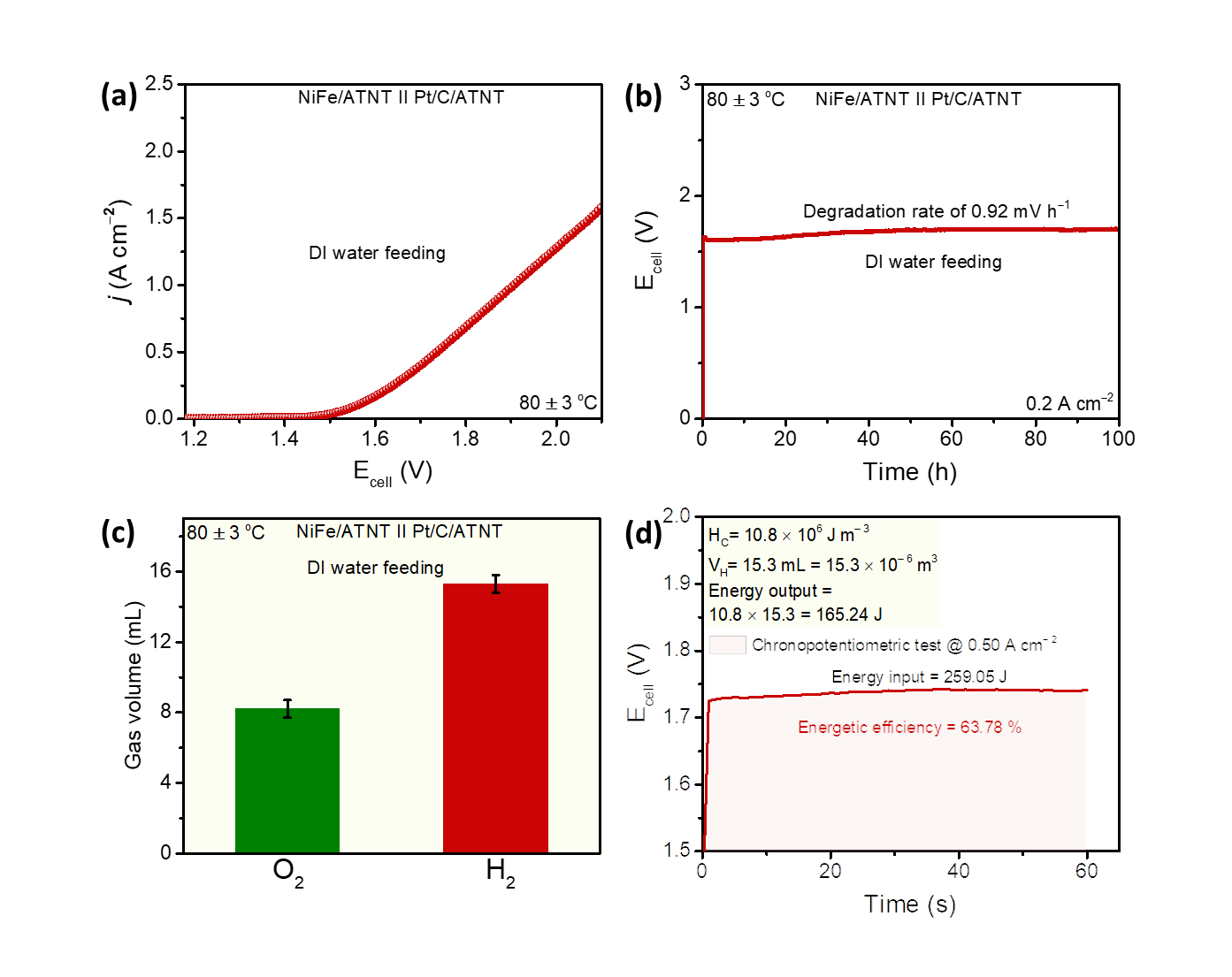


**Fig. S32** AEMWE performance of the NiFe/ATNT ‖ Pt/C/ATNT configuration with DI water feed (3 mL min⁻^1^) at 80 ± 3 °C. **a** LSV curve at a scan rate of 5 mV s⁻^1^. **b** Stability test at 0.2 A cm⁻^2^ for 100 h. **c** The generated O_2_ and H_2_ gas volumes during **d** a chronopotentiometric test at a current density of 0.50 A cm⁻^2^ for 60 s with DI water. Energy output was calculated based on the calorific value of H_2_


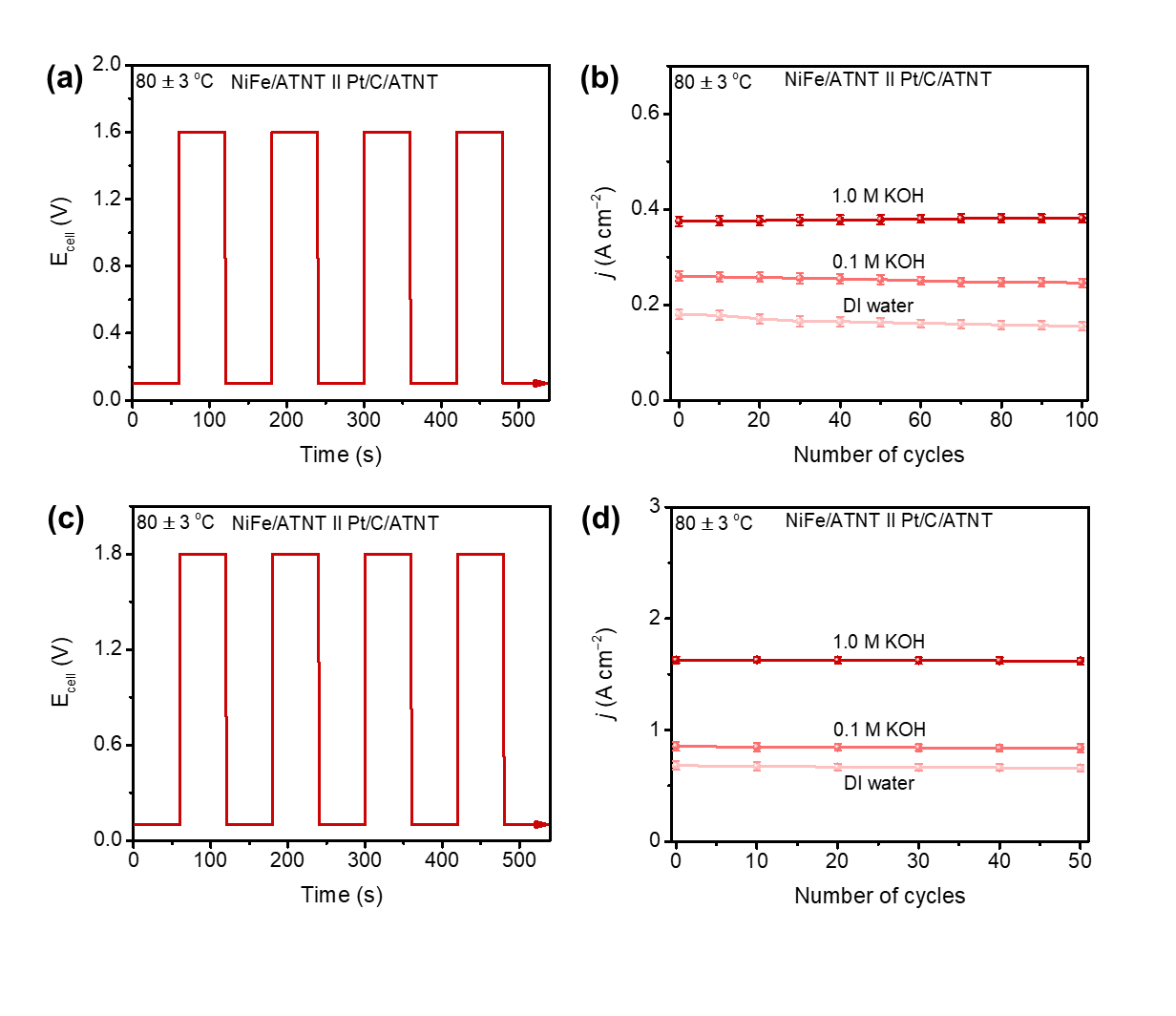


**Fig. S33** Dynamic operational durability of the AEMWE system with NiFe/ATNT ‖ Pt/C/ATNT configuration using different electrolytes. **a** Square-wave voltage cycling pattern (0.10 - 1.60 V) used for the start-stop cyclic durability test at 80 ± 3 °C. **b** Variation in current density at 1.60 V as a function of the number of start-stop cycles. **c** Square-wave voltage cycling pattern (0.10 - 1.80 V) used for the start-stop cyclic durability test at 80 ± 3 °C. **d** Variation in current density at 1.80 V as a function of the number of start-stop cycles

**Table S1** Optimization of bath composition for electrodeposition of NiFe catalysts with varying Ni:Fe ratios

| Ni:Fe  molar ratio | NiSO_4_·6H_2_O  (g) | FeSO_4_·7H_2_O  (g) | Current density  (mA cm⁻²) | Deposition  time (s) | Temperature  (^o^C) | pH |
| --- | --- | --- | --- | --- | --- | --- |
| 1:1 | 2.64 | 2.64 | 100 | 180 | 40 + 2 | 7 |
| 2:1 | 2.64 | 1.32 | 100 | 180 | 40 + 2 | 7 |
| 4:1 | 2.64 | 0.65 | 100 | 180 | 40 + 2 | 7 |
| 6:1 | 2.64 | 0.45 | 100 | 180 | 40 + 2 | 7 |

**Table S2** Optimization of deposition time for the bath to fabricate NiFe catalysts with varying loading amounts during electrodeposition

| **Metal loading**  **(mg/cm^2^)** | **NiSO_4_·6H_2_O**  **(g)** | **FeSO_4_·7H_2_O**  **(g)** | **Current density (mA cm⁻²)** | **Deposition time (s)** | **Temperature (^o^C)** | **pH** |
| --- | --- | --- | --- | --- | --- | --- |
| 1 | 2.64 | 0.65 | 100 | 40 | 40 + 2 | 7 |
| 3 | 2.64 | 0.65 | 100 | 100 | 40 + 2 | 7 |
| 5 | 2.64 | 0.65 | 100 | 180 | 40 + 2 | 7 |
| 10 | 2.64 | 0.65 | 100 | 300 | 40 + 2 | 7 |

**Table S3** Molar ratio of prepared electrodes

| **Sample** | **Fe** | **Ni** |
| --- | --- | --- |
| NiFe/TF | 1 | 4.013 |
| NiFe/TNT | 1 | 4.027 |
| NiFe/ATNT | 1 | 4.008 |

**Table S4** Electrical resistivity of as-prepared electrodes

| **Sample** | **Thickness (cm)** | **Electrical resistivity (mΩ cm)** |
| --- | --- | --- |
| TF | 0.052 | 0.54 |
| NiFe/TF | 0.052 | 0.50 |
| TNT | 0.052 | 1.37 |
| NiFe/TNT | 0.052 | 1.18 |
| ATNT | 0.052 | 1.35 |
| NiFe/ATNT | 0.052 | 1.22 |

**Table S5** Comparison of AEMWE performance in 1.0 M KOH

| **Anode** | **Cathode** | **Current density**  **(A cm^−2^) @**  **Cell Voltage (V)** | **Stability**  **(h)** | **Degradation rate**  **(mV h^−1^)** | **Refs.** |
| --- | --- | --- | --- | --- | --- |
| **NiFe** | **Pt/C** | **1.67 @ 1.80** | **1500** | **~0.20** | **This work** |
| NiFe_2_O_4_ | Pt/C | 1.43 @1.80 | 72 | ~7.4 mA h⁻^1^ | [S6] |
| CuCo-oxide | Pt/C | 1.39 @1.80 | 64 | ~1.25 | [S7] |
| Ni_0.75_Fe_2.25_O_4_ | Pt/C | 1.31 @1.80 | 21 | ~9.52 | [S8] |
| CuCo_2_O_4_ | Pt/C | ~1.00@1.80 | 12 | ~1.83 | [S9] |
| Cu_0.5_Co_2.5_O_4_ | Pt/C | 1.30 @1.80 | 100 | ~0.5 | [S10] |
| H-Co_0.9_Fe_0.1_-CNF | Pt/C | 0.79 @1.70 | 290 | ~0.173 | [S11] |
| CoSb_2_O_6_ | Pt/C | 1.00 @ 1.98 | - | - | [S12] |
| NiFe‐LDH | Pt/C | 1.00 @ 1.69 | 50 | ~3.8 | [S13] |
| (NiCo)_3_Se_4_ | Pt/C | 1.00 @ 1.75 | 95 | ~4 | [S14] |
| NiFe‐LDH | Pt/C | 1.00 @ 1.59 | 6 | ~3.3 | [S15] |
| Cu_0.81_Co_2.19_O_4_ | Co_3_S_4_ | 1.00 @ 2.20 | 12 | ~8.3 | [S16] |
| Fe-NiMo-NH_3_/H_2_ | NiMo-NH_3_/H_2_ | 1.00 @ 1.57 | 25 | ~1.6 | [S17] |
| Fe_0.2_Ni_0.8_-P_0.5_S_0.5_ | Fe_0.2_Ni_0.8_-P_0.5_S_0.5_ | 2.50 @ 2.00 | 300 | ~0.08 | [S18] |
| NiFe | NiFe | 1.60 @ 2.00 | 1000 | ~0 | [S19] |
| CoCrOx | Pt/C | 1.50 @ 2.10 | 120 | ~4.9 | [S20] |
| (FeCoMnZnMg)_3_O_4_ | Pt/C | 1.00 @ 1.86 | 40 | ~8.75 mA cm⁻^2^ h⁻^1^ | [S21] |
| Ni_2_P/Ni_7_S_6_ | Pt/C | 1.00 @ 1.88 | 140 | ~1.9 | [S22] |
| Ni_2_Fe_8_/Ni_3_S_2_/NF | Ni_4_Mo/MoO_2_/NF | 1.00 @ 1.65 | 100 | ~1.3 | [S23] |
| Ni_2_P @ FePO_x_H_y_ | MoNi_4_/MoO_2_ | 1.00 @ 1.84 | 72 | ~0 | [S23] |
| NA-LT-CA | NA-LT-CA | 0.50 @1.85 | 1000 | ~0 | [S25] |
| P-CoVO@NF | CoN/VN@NF | 0.50 @1.76 | 1000 | ~5 mA cm⁻^2^ h⁻^1^ | [S26] |
| Co_2.8_, W_3.8_-NiFe LDH | Pt/C | 1.00 @1.86 | 300 | ~0 | [S27] |
| FeNi_3_/FeNiO | FeNi_3_/FeNiO | 1.00 @1.94 | 150 | ~0.88 | [S28] |
| d-(Fe,Ni)OOH | NiMoN | 0.50 @1.80 | 96 | ~0.52 | [S29] |
| NiFeS@Ti_3_C_2_ MXene/NF | NiFeS@Ti_3_C_2_ MXene/NF | 0.40 @1.85 | 35 | ~0.4 mA h⁻^1^ | [S30] |
| S–NiFe LDH | Pt@S–NiFe LDH | 0.01 @1.62 | 200 | ~1.50 | [S31] |
| B-MOF-ZnCo | Pt/C | 1.00 @2.04 | 300 | ~0.2 | [S32] |

**Supplementary References**

[S1] S. S. Jeon, P. W. Kang, M. Klingenhof, H. Lee, F. Dionigi et al., Active surface area and intrinsic catalytic oxygen evolution reactivity of NiFe LDH at reactive electrode potentials using capacitances. ACS Catal. **13**, 1186 (2023). <https://doi.org/10.1021/acscatal.2c04452>

[S2] B. Kim, M.K. Kabiraz, J. Lee, C. Choi, H. Baik et al., Vertical-crystalline Fe-doped β-Ni oxyhydroxides for highly active and stable oxygen evolution reaction. Matter **4**, 3585 (2021). <https://doi.org/10.1016/j.matt.2021.09.003>

[S3] P. Haug, M. Koj, T. Turek, Influence of process conditions on gas purity in alkaline water electrolysis. Int. J. Hydrogen Energy **42**, 9406 (2017). <https://doi.org/10.1016/j.ijhydene.2016.12.111>

[S4] I. Vincent, E.-C. Lee, H.-M. Kim, Comprehensive impedance investigation of low-cost anion exchange membrane electrolysis for large-scale hydrogen production. Sci. Rep. **11**, 293 (2021). <https://doi.org/10.1038/s41598-020-80683-6>

[S5] Q. Xu, L. Zhang, J. Zhang, J. Wang, Y. Hu et al., Anion exchange membrane water electrolyzer: electrode design, lab-scaled testing system and performance evaluation. EnergyChem **4**, 100087 (2022). <https://doi.org/10.1016/j.enchem.2022.100087>

[S6] A. Martinez-Lazaro, A. Caprì, I. Gatto, J. Ledesma-García, N. Rey-Raap et al., NiFe_2_O_4_ hierarchical nanoparticles as electrocatalyst for anion exchange membrane water electrolysis. J. Power Sources **556**, 232417 (2023). <https://doi.org/10.1016/j.jpowsour.2022.232417>

[S7] Y.S. Park, J. Yang, J. Lee, M.J. Jang, J. Jeong et al., Superior performance of anion exchange membrane water electrolyzer: ensemble of producing oxygen vacancies and controlling mass transfer resistance. Appl. Catal. B Environ. **278**, 119276 (2020). <https://doi.org/10.1016/j.apcatb.2020.119276>

[S8] J. Lee, H. Jung, Y.S. Park, S. Woo, N. Kwon et al., Corrosion-engineered bimetallic oxide electrode as anode for high-efficiency anion exchange membrane water electrolyzer. Chem. Eng. J. **420**, 127670 (2021). <https://doi.org/10.1016/j.cej.2020.127670>

[S9] Y.S. Park, M.J. Jang, J. Jeong, S.M. Park, X. Wang et al., Hierarchical chestnut-burr like structure of copper cobalt oxide electrocatalyst directly grown on Ni foam for anion exchange membrane water electrolysis. ACS Sustain. Chem. Eng. **8**, 2344 (2020). <https://doi.org/10.1021/acssuschemeng.9b06767>

[S10] M.J. Jang, J. Yang, J. Lee, Y.S. Park, J. Jeong et al., Superior performance and stability of anion exchange membrane water electrolysis: pH-controlled copper cobalt oxide nanoparticles for the oxygen evolution reaction. J. Mater. Chem. A **8**, 4290 (2020). <https://doi.org/10.1039/C9TA13137J>

[S11] S. Kang, K. Ham, J. Lee, Moderate oxophilic CoFe in carbon nanofiber for the oxygen evolution reaction in anion exchange membrane water electrolysis. Electrochim. Acta **353**, 136521(2020). <https://doi.org/10.1016/j.electacta.2020.136521>

[S12] K. Ham, S. Hong, S. Kang, K. Cho, J. Lee, Extensive active-site formation in trirutile CoSb_2_O_6_ by oxygen vacancy for oxygen evolution reaction in anion exchange membrane water splitting. ACS Energy Lett. **6**, 364 (2021). <https://doi.org/10.1021/acsenergylett.0c02359>

[S13] S.S. Jeon, J. Lim, P.W. Kang, J.W. Lee, G. Kang et al., Design principles of NiFe-layered double hydroxide anode catalysts for anion exchange membrane water electrolyzers. ACS Appl. Mater. Interfaces **13**, 37179 (2021). <https://doi.org/10.1021/acsami.1c09606>

[S14] J. Abed, S. Ahmadi, L. Laverdure, A. Abdellah, C.P. O’Brien et al., In situ formation of nano Ni-Co oxyhydroxide enables water oxidation electrocatalysts durable at high current densities. Adv. Mater. **33**, 2103813 (2021). <https://doi.org/10.1002/adma.202103812>

[S15] H. Koshikawa, H. Murase, T. Hayashi, K. Nakajima, H. Mashiko et al., Single nanometer-sized NiFe-layered double hydroxides as anode catalyst in anion exchange membrane water electrolysis cell with energy conversion efficiency of 74.7% at 1.0 A cm^–2^. ACS Catal. **10**, 1886 (2020). <https://doi.org/10.1021/acscatal.9b04505>

[S16] Y.S. Park, J.H. Lee, M.J. Jang, J. Jeong, S.M. Park et al., Co_3_S_4_ nanosheets on Ni foam via electrodeposition with sulfurization as highly active electrocatalysts for anion exchange membrane electrolyzer. Int. J. Hydrogen Energy **45**, 36 (2020). <https://doi.org/10.1016/j.ijhydene.2019.10.169>

[S17] P. Chen, X. Hu, High-efficiency anion exchange membrane water electrolysis employing non-noble metal catalysts. Adv. Energy Mater. **10**, 2002285 (2020). <https://doi.org/10.1002/aenm.202002285>

[S18] L. Wan, Z. Xu, P. Wang, P.-F. Liu, Q. Xu et al., Dual regulation both intrinsic activity and mass transport for self-supported electrodes using in anion exchange membrane water electrolysis. Chem. Eng. J. **431**, 133942 (2022). <https://doi.org/10.1016/j.cej.2021.133942>

[S19] N. Chen, S.Y. Paek, J.Y. Lee, J.H. Park, S.Y. Lee et al., High-performance anion exchange membrane water electrolyzers with a current density of 7.68 A cm^−2^ and a durability of 1000 hours. Energy Environ. Sci. **14**, 6338 (2021). <https://doi.org/10.1039/D1EE02642A>

[S20] S. Li, T. Liu, W. Zhang, M. Wang, H. Zhang et al., Highly efficient anion exchange membrane water electrolyzers via chromium-doped amorphous electrocatalysts. Nat. Commun **15**, 3416 (2024). <https://doi.org/10.1038/s41467-024-47736-0>

[S21] S. C. Karthikeyan, S. Ramakrishnan, S. Prabhakaran, M. R. Subramaniam, M. Mamlouk et al., Low-cost self-reconstructed high entropy oxide as an ultra-durable OER electrocatalyst for anion exchange membrane water electrolyzer, Small **20**, 2402241 (2024). <https://doi.org/10.1002/smll.202402241>

[S22] F.-L. Wang, N. Xu, C.-J. Yu, J.-Y. Xie, B. Dong et al., Porous heterojunction of Ni_2_P/Ni_7_S_6_ with high crystalline phase and superior conductivity for industrial anion exchange membrane water electrolysis. Appl. Catal. B Environ. **330**, 122633 (2023). <https://doi.org/10.1016/j.apcatb.2023.122633>

[S23] G. Ding, H. Lee, Z. Li, J. Du, L. Wang et al., Highly efﬁcient and durable anion exchange membranewater electrolyzer enabled by a Fe–Ni_3_S_2_ anode catalyst, Adv. Energy Sustainability Res. **4**, 2200130 (2023). https://doi.org/10.1002/aesr.202200130

[S24] A. Meena, P. Thangavel, D. S. Jeong, A. N. Singh, A. Jana et al., Crystalline-amorphous interface of mesoporous Ni_2_P @ FePO_x_H_y_ for oxygen evolution at high current density in alkaline-anion-exchange-membrane water-electrolyzer. Appl. Catal. B-Environ. **306**, 121127 (2022). <https://doi.org/10.1016/j.apcatb.2022.121127>

[S25] X. Jiang , V. Kyriakou , B. Wang, S. Deng, S. Costil et al., Hierarchical microporous Ni-based electrodes enable “two birds with one stone” in highly efficient and robust anion exchange membrane water electrolysis (AEMWE). Chemical Engineering Journal **486** ,150180 (2024). <https://doi.org/10.1016/j.cej.2024.150180>

[S26] [Z. Liang](https://advanced.onlinelibrary.wiley.com/authored-by/Liang/Zhijian), [D. Shen](https://advanced.onlinelibrary.wiley.com/authored-by/Shen/Di), [Y. Wei](https://advanced.onlinelibrary.wiley.com/authored-by/Wei/Yao), [F. Sun](https://advanced.onlinelibrary.wiley.com/authored-by/Sun/Fanfei), [Y. Xie](https://advanced.onlinelibrary.wiley.com/authored-by/Xie/Ying) et al., Modulating the electronic structure of cobalt-vanadium bimetal catalysts for high-stable anion exchange membrane water electrolyzer. Adv. Mater. **36**, 2408634 (2024). <https://doi.org/10.1002/adma.202408634>

[S27] Y. Shi, L. Song, Y. Liu, T. Wang, C. Li et al., Dual cocatalytic sites synergize NiFe layered doublehydroxide to boost oxygen evolution reaction in anionexchange membrane water electrolyzer. Adv. Energy Mater. **14**, 2402046 (2024). <https://doi.org/10.1002/aenm.202402046>

[S28] F. Malaj, A. Tampucci, D. Lentini, L. Brogi, E. Berretti et al., One-pot synthesis of FeNi_3_/FeNiO_x_ nanoparticles for PGM-free anion exchange membrane water electrolysis. Electrochimica Acta **507**, 145109 (2024). <https://doi.org/10.1016/j.electacta.2024.145109>

[S29] L. Wu, M. Ning, X. Xing, Y. Wang, F. Zhang et al., Boosting oxygen evolution reaction of (Fe,Ni)OOH via defect engineering for anion exchange membrane water electrolysis under industrial conditions. Adv. Mater. **35**, 2306097 (2023). <https://doi.org/10.1002/adma.202306097>

[S30] D. Chanda, K. Kannan, J. Gautam, M. M. Meshesha, S. G. Jang et al., Effect of the interfacial electronic coupling of nickel-iron sulfide nanosheets with layer Ti_3_C_2_ MXenes as efficient bifunctional electrocatalysts for anion-exchange membrane water electrolysis, Applied Catalysis B: Environmental **321**, 122039 (2023). <https://doi.org/10.1016/j.apcatb.2022.122039>.

[S31] H. Lei, Q. Wan, S. Tan, Z. Wang, W. Mai, Pt-quantum-dot-modified sulfur-doped NiFe layered double hydroxide for high-current-density alkaline water splitting at industrial temperature. Adv. Mater., **35**, 2208209 (2023). <https://doi.org/10.1002/adma.202208209>

[S32] X. Lin, X. Li, L. Shi, F. Ye, F. Liu et al., In situ electrochemical restructuring B-doped metal–organic frameworks as eﬃcient OER electrocatalysts for stable anion exchange membrane water electrolysis. Small **20**, 2308517 (2024). <https://doi.org/10.1002/smll.202308517>
